# MEP Pathway: First-Synthesized IspH-Directed Prodrugs with Potent Antimycobacterial Activity

**DOI:** 10.3390/microorganisms14010215

**Published:** 2026-01-17

**Authors:** Alizée Allamand, Ludovik Noël-Duchesneau, Cédric Ettelbruck, Edgar De Luna, Didier Lièvremont, Catherine Grosdemange-Billiard

**Affiliations:** 1 Laboratoire Chimie et Biochimie de Molécules Bioactives, Université de Strasbourg/CNRS, UMR 7177, Institut Le Bel, 4 rue Blaise Pascal, 67081 Strasbourg, Francenoelduchesneau@unistra.fr (L.N.-D.); edelunagomez@udallas.edu (E.D.L.); didier.lievremont@unistra.fr (D.L.); 2 Unité Molécules de Communication et Adaptation des Micro-organismes (UMR 7245), Sorbonne Université, Muséum National d’Histoire Naturelle, CNRS, 75005 Paris, France; 3 Dallas University, 1845 E. North Gate Dr., Irving, TX 75062, USA

**Keywords:** isoprenoid biosynthesis, 4-hydroxy-3-methylbut-2-enyl diphosphate reductase, *cyclo*Saligenyl prodrug, *Mycobacterium smegmatis*, antimycobacterial, isoniazid

## Abstract

We report the first synthesis of IspH-directed prodrugs targeting the terminal enzyme of the 2-*C*-methyl-D-erythritol 4-phosphate (MEP) pathway, (*E*)-4-hydroxy-3-methylbut-2-enyl diphosphate reductase (IspH or LytB). A series of alkyne and pyridine monophosphate *cyclo*Saligenyl (*cyclo*Sal) prodrugs were prepared to enhance membrane permeability by masking the phosphate group. The effects of electron-withdrawing (Cl, CF_3_) and electron-donating (OCH_3_, NH_2_) substituents were examined, together with amino acid-functionalized and mutual prodrug analogs. Among the synthesized compounds, chlorine-substituted derivatives **5c** and **6c** displayed the strongest antimycobacterial activity against *Mycobacterium smegmatis*, surpassing isoniazid in agar diffusion assays. These results indicate that electron-withdrawing substituents accelerate prodrug hydrolysis and facilitate intracellular release of the active inhibitor. This work provides the first experimental evidence of an IspH-targeted prodrug approach, highlighting the *cyclo*Sal strategy as a valuable tool for delivering phosphorylated inhibitors and developing novel antimycobacterial agents acting through the MEP pathway.

## 1. Introduction

Antimicrobial resistance (AMR), caused mainly by the misuse and overuse of antimicrobial agents in humans, animals and agriculture, has emerged as one of the major public health threats of the 21st century. In 2019, bacterial AMR was directly responsible for an estimated 1.27 million deaths globally and associated with a further 5 million deaths, highlighting its severe impact on health worldwide [1].

The ESKAPEE pathogens (*Enterococcus faecium*, *Staphylococcus aureus*, *Klebsiella pneumoniae*, *Acinetobacter baumannii*, *Pseudomonas aeruginosa*, *Enterobacter* spp. and *Escherichia coli)* along with *Mycobacterium tuberculosis* (*Mtb*) are particularly notorious for their high levels of antibiotic resistance [2].

The multidrug-resistant (MDR) and extensively drug-resistant strains of *Mtb* are prime examples. Globally, 73% of people who tested positive for tuberculosis (TB) in 2022 developed multidrug-resistant tuberculosis, as the strains of *Mtb* do not respond to isoniazid (INH) and rifampicin, the two most effective first-line antitubercular drugs discovered over 60 years ago [3]. Despite 90 years of vaccination, 70 years of antibacterial chemotherapy and significant progress in developing a TB drug pipeline, TB remains one of the top ten causes of death worldwide [4]. Therefore, there is a critical need to discover and develop new antitubercular compounds with novel modes of action that can cross the robust waxy cell wall of *Mtb*.

The enzymes of the 2-*C*-methyl-D-erythritol 4-phosphate (MEP) pathway for the biosynthesis of isoprenoids represent attractive targets for the development of a novel class of antimicrobials, given their absence in humans and notable presence in six pathogens belonging to the ESKAPEE group as well as in *Mtb* [5]. However, the design of drugs must account for the high hydrophobicity of catalytic sites hosting the charged mono- and diphosphates moieties of the substrates of the MEP pathway enzymes.

To date, the most extensively studied therapeutic target of the MEP pathway is the second enzyme, 1-deoxy-D-xylulose 5-phosphate reductoisomerase (DXR or IspC). A significant number of publications reported in the literature aimed to improve the efficiency and bioavailability of fosmidomycin, a natural and potent DXR inhibitor, as well as its natural and synthetic analogs [6]. Within this framework, we recently reported for the first time that the *cyclo*Saligenyl prodrug strategy increased the bioavailability of fosmidomycin phosphate analogs in mycobacteria by preventing the growth of *M. smegmatis*, a non-pathogenic model of *Mtb* [7].

**Scheme 1:**
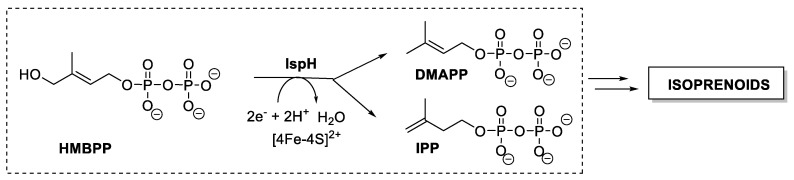
Last enzyme of the 2-*C*-methyl-D-erythritol 4-phosphate (MEP) pathway.

In this work, we focused in applying this strategy to inhibitors of the last enzyme of the MEP pathway, IspH (LytB), a [4Fe-4S] cluster-dependent reductase that catalyzes the reduction of (*E*)-4-hydroxy-3-methyl-but-2-enyl pyrophosphate (HMBPP) to a mixture of the two universal isoprenoid precursors, isopentenyl diphosphate (IPP) and dimethylallyl diphosphate (DMAPP) in a ratio of approximately 6:1 (Scheme 1) [8].

**Scheme 2:**
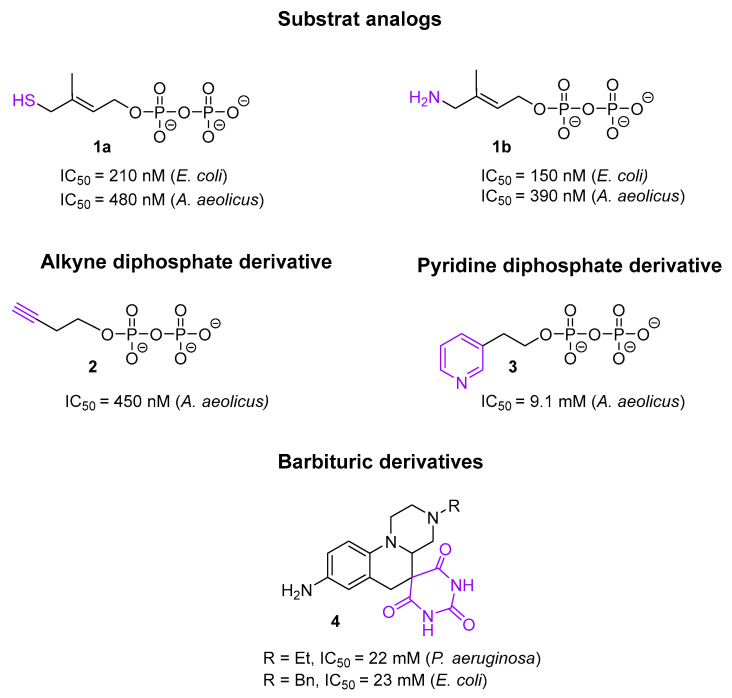
Examples of (*E*)-4-hydroxy-3-methylbut-2-enyl diphosphate reductase (IspH) inhibitors.

In contrast to DXR, the literature on inhibitors of the oxygen-sensitive metalloenzyme IspH is limited, which can be explained by the recent elucidation of its catalytic mechanism only a decade ago. Most of the known inhibitors interact with the apical iron of the [4Fe-4S] cluster and can be classified into four categories: substrate analogues **1** [9], alkyne derivatives **2** [10], pyridine diphosphate derivatives **3** and non-diphosphate compounds with a barbituric core **4** selected via in silico approach (Scheme 2) [11]. They are efficient inhibitors with inhibitory concentrations ranging from low micromolar to high nanomolar against the IspH enzymes of *E. coli* and *Aquifex aeolicus*, an unusual hyperthermophilic and microaerophilic bacterium.

However, none of these inhibitors have yet been tested for bacterial growth inhibition. In fact, although the diphosphate group in its polyanionic form at physiological pH is essential for biological activity, it restricts passive diffusion across the cell membrane. Another concern is the fast hydrolysis of the diphosphate group by enzymes such as phosphatases. To increase the bioavailability and to circumvent the lack of cellular uptake, the prodrug strategy has been developed by masking the charged phosphate moiety. Prodrugs of diphosphates have been used almost exclusively in the context of nucleotide antiviral and anticancer drugs [12]. Since the limiting step in activating nucleoside analogs is often the initial phosphorylation, various prodrugs of the corresponding monophosphates have been explored using different masking groups, e.g., phosphoramidate and *cyclo*Saligenyl (*cyclo*Sal), to facilitate the delivery of neutral nucleotides into the cell (pronucleotide or ProTide approach) [13,14].

Once inside the cell, the chemical or enzymatic cleavage occurs, releasing the monophosphate nucleotide and allowing further phosphorylations. This ProTide strategy could be implemented to evaluate in vivo the biological activity of IspH diphosphate inhibitors on a panel of bacterial strains. The choice of the phosphate masking group is based on our previous works on the DXR prodrug inhibitors. The aryl phosphoramidate moiety, widely used for the delivery of monophosphorylated nucleoside analogs to treat viral infections and cancer [15], might not be appropriate to deliver non-nucleotide phosphate compounds. Indeed, phosphoramidate prodrugs of DXR inhibitors are ineffective in inhibiting the growth of bacteria such as *E. coli* or *M. smegmatis* [16]. Futhermore, the main drawback of this approach is its reliance on one or more intracellular enzymes (esterases, carboxypeptidases and phosphoramidases) to release the inhibitors within the cell.

In contrast, the *cyclo*Sal strategy developed by C. Meier [15,16,17], in which the phosphate is masked with a salicylic alcohol moiety, improves the permeability of monophosphate nucleosides. It also facilitates intracellular nucleotide delivery via an exclusively pH-dependent hydrolysis, after which the released monophosphate can be further phosphorylated. We implemented this technique to DXR phosphate inhibitors and it proved effective to prevent the growth of *M. smegmatis* [7].

**Scheme 3:**
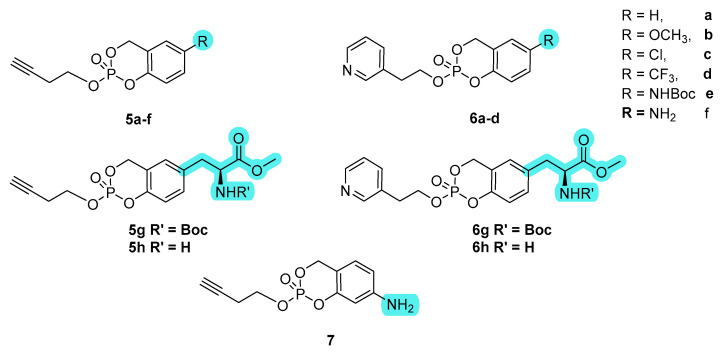
Targeted *cyclo*Saligenyl (*cyclo*Sal) prodrugs **5**–**7**.

Here, we report the synthesis of a series of alkyne and pyridine monophosphate *cyclo*Sal prodrugs **5** and **6** and evaluate their growth inhibition potency against a panel of bacteria including the non-pathogenic, fast-growing *M. smegmatis* (Scheme 3). Once inside the cell, the released monophosphate derivative is expected to undergo further phosphorylation by bacterial kinases, yielding the corresponding diphosphate form. This diphosphate metabolite then acts as the active inhibitor of IspH (LytB), the terminal enzyme of the MEP pathway. Since the kinetic hydrolysis is modulated by the substituent on the aromatic ring at C-5, we investigated the influence of electron-withdrawing (Cl, CF_3_) and electron-donating (OCH_3,_ NH_2_) substituents on the bacterial growth inhibition activity of these *cycl*oSaligenyl prodrugs.

**Scheme 4:**
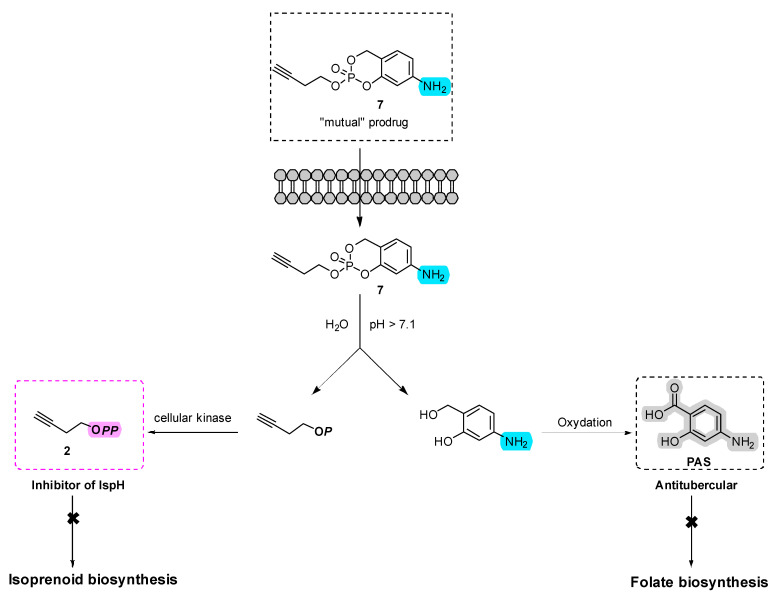
Mutual prodrug strategy.

To expand the chemical versatility of this *cyclo*Sal scaffold, we envisaged to synthesize the compounds **5h** and **6h**, functionalized with an amino acid residue. This modification provides a chemical for tethering an additional bioactive agent through the amino acid side chain. In this design, the aryl ring of the *cyclo*Sal structural motif acts as a multifunctional platform, enabling the construction of dual-action prodrugs, also known as mutual prodrugs, which target multiple metabolic pathways. Considering this mutual prodrug approach further, we chose to synthesize compound **7** in which the phosphate group of an IspH inhibitor core is masked by *p*-aminosalicylic alcohol (Scheme 3). Once inside the bacteria, the mutual prodrug is expected to be hydrolyzed, thereby simultaneously releasing (*i*) the IspH monophosphate inhibitor derivative which might be subsequently phosphorylated by cellular kinases to generate the active diphosphate form, thus inhibiting the biosynthesis of isoprenoids in the mycobacteria, and (*ii*) a reduced derivative of *p*-aminosalicylic acid (PAS), one of the earliest antitubercular drugs introduced into clinical use which remains part of multidrug-resistant TB treatment regimens (Scheme 4) [15]. Upon subsequent oxidation of the primary alcohol group, PAS may be regenerated in its active form. PAS acts by inhibiting dihydropteroate synthase (DHPS), a key enzyme in folate biosynthesis, thus blocking the production of tetrahydrofolate cofactors which are essential for mycobacterial growth [18].

## 2. Experimental Section

### 2.1. Chemistry

#### General Methods

All non-aqueous reactions were run under argon atmosphere, using dry solvents. Commercial grade reagents were purchased from Sigma-Aldrich (St. Louis, MO, USA), TCI, BLDPharm or Acros Organics and used without further purification. Petroleum ether (PE) 40–60 °C (Sigma-Aldrich) was used for purification.

Flash chromatography was performed on silica gel 60 (230–400 mesh) from Merck (Burlington, MA, USA) with the solvent system as indicated. Automated flash chromatography was performed on silica gel (30 or 50 μm) on a Puriflash 215 (Interchim, Montluçon, France).

TLC plates were revealed under UV light (254 or 366 nm) and/or by spraying with a cerium molybdate solution or an ethanolic solution of potassium permanganate followed by heating.

The NMR spectra were recorded on a Bruker Avance 300 (^1^H-NMR: 300 MHz; ^31^P-NMR: 121.5 MHz; ^19^F-NMR: 282.4 MHz) or a Bruker Avance 400 (^1^H-NMR: 400 MHz; ^31^P-NMR: 161.9 MHz; ^19^F-NMR: 376.4 MHz) or a Bruker Avance 500 (^1^H-NMR: 500 MHz; ^31^P-NMR: 202.4 MHz; ^13^C-NMR: 125.8 MHz) spectrometer. Chemical shifts are expressed in parts per million (ppm) and were calibrated to deuterated or residual non-deuterated solvent peaks for ^1^H and ^13^C spectra. s, bs, d, t, bt, q, p and m are abbreviations for multiplicity corresponding to singlet or sextuplet, broad singlet, doublet, triplet, broad triplet, quadruplet or quintuplet, pentuplet and multiplet. *J*-couplings are expressed in Hz.

Negative or positive mode electrospray MS were performed on a Bruker Daltonics microTOF spectrometer (Bruker Daltonik GmgH, Bremen, Germany) equipped with an orthogonal electrospray (ESI) interface. Calibration was performed using a solution of 10 mM sodium formate. Sample solutions were introduced into the spectrometer source with a syringe pump (Harvard type 55 1111: Harvard Apparatus Inc., South Natick, MA, USA) with a flow rate of 5 µL·min^−1^.

### 2.2. General Procedures

**A/General procedure for the synthesis of *cyclo*Sal phosphochloridate**

A solution of phosphorous oxichloride (1.1 eq) in THF (1.3 mL/mmol) was cooled down to −78 °C. A solution of saligenol (1 eq) and triethylamine (2.1 eq) in THF (2 mL/mmol) was added dropwise and the mixture was allowed to slowly warm up to reach approximately 10 °C overnight. After completion, the colorless solid was filtered under argon and the solvent evaporated under reduced pressure. The crude was directly engaged in the next step.

**B/General procedure for the synthesis of *cyclo*Sal phosphotriester**

A solution of DMAP (0.5 eq), alcohol (1 eq) and triethylamine (1.1 eq) in DCM (3 mL/mmol) was cooled down to −40 °C, and a solution of *cyclo*Sal phosphochloridate (3 eq) in DCM (0.8 mL/mmol) was added dropwise. The reaction mixture was allowed to warm up to room temperature and stirred overnight.

Workup 1:

A saturated solution of NH_4_Cl was added, and the aqueous layer was extracted with DCM. The combined organic layers were dried over anhydrous sodium sulfate (Na_2_SO_4_), and the solvent was evaporated under reduced pressure.

Workup 2:

A saturated solution of NaCl was added. Then, the aqueous layer was extracted with DCM, basified with a saturated NaHCO_3_ solution to a pH around 6 and extracted again with DCM. These last combined organic layers were dried over Na_2_SO_4,_ and the solvent was evaporated under reduced pressure.

***tert*-Butyl (4-hydroxy-3-(hydroxymethyl)phenyl)carbamate (8e)**

To a solution of methyl 2-hydroxy-5-nitrobenzoate (5.0 g, 25.3 mmol) in a mixture of ethyl acetate (150 mL) and methanol (15 mL), a Pd/C catalyst (0.5 g, 10 wt%) was added under argon. The reaction mixture was carefully evacuated from argon, filled with hydrogen and then stirred overnight at room temperature. After completion, the reaction mixture was filtered on a pad of Celite, and the solvent was evaporated under reduced pressure to afford methyl 5-amino-2-hydroxybenzoate (cas: 42753-75-3) as brownish solid (3.86 g, 91%). The ^1^H NMR spectrum is in agreement with previously reported data [19].

**^1^H NMR (300 MHz, chloroform-d):** δ 3.37 (bs, 2H, NH_2_), 3.93 (s, 3H, OCH_3_), 6.83 (dd, *J* = 8.8, 0.6 Hz, 1H, CH_Ar_), 6.88 (dd, *J* = 8.8, 2.8 Hz, 1H, CH_Ar_), 7.16 (dd, *J* = 2.8, 0.6 Hz, 1H, CH_Ar_), 10.19 (s, 1H, OH).

To the crude methyl 5-amino-2-hydroxybenzoate (1 eq, 3.86 g, 23.1 mmol), neat Boc_2_O (1.2 eq, 6.37 mL, 27.7 mmol) was added, and the reaction mixture was stirred at rt for 1 h leading to a brown solid which was dissolved in DCM. The organic layer was washed with water and a saturated solution of NaHCO_3_ prior to being dried over Na_2_SO_4_ and evaporated to give methyl 5-((*tert*-butoxycarbonyl)amino)-2-hydroxybenzoate (cas: 942404-97-9) (6.74 g, quant. yield).

**^1^H NMR (300 MHz, chloroform-d):** δ 1.51 (s, 9H, C(CH_3_)_3_), 3.94 (s, 3H, OCH_3_), 6.35 (br. s, 1H, NH), 6.92 (d, *J* = 8.9 Hz, 1H, CH_Ar_), 7.36 (dd, *J* = 8.9, 2.8 Hz, 1H, CH_Ar_), 7.92 (d, *J* = 2.5 Hz, 1H, CH_Ar_), 10.53 (s, 1H, OH).

To a solution of methyl 5-((*tert*-butoxycarbonyl)amino)-2-hydroxybenzoate (1 eq, 1.4 g, 5.2 mmol) in dry THF (50 mL) cooled at −80 °C, LiAlH_4_ was added dropwise (2.4 eq, 12 mL; 1 M solution in THF, 12 mmol). The mixture was stirred and allowed to warm to room temperature overnight. The reaction mixture was quenched by a careful addition of *ca*. 100 mL of seignette salt’s saturated solution. The aqueous phase was acidified to pH = 6 by addition of 2 M HCl and then extracted three times with Et_2_O. Organic phases were combined, dried over Na_2_SO_4_, filtered and evaporated under reduced pressure. The crude was purified by automated flash chromatography (from 95:5 PE/EtOAc to 70:30 PE/EtOAc) to afford compound **8e** (858 mg, 70%) as a beige solid. The ^1^H NMR spectrum is in agreement with previously reported data [20].

**^1^H NMR (300 MHz, chloroform-*d*):** δ 1.50 (s, 9H, C(CH_3_)_3_), 4.82 (s, 2H, OCH_2_), 6.30 (s, 1H, NH), 6.81 (d, *J* = 8.6 Hz, 1H, CH_Ar_), 7.01 (dd, *J* = 8.6, 2.7 Hz, 1H, CH_Ar_), 7.14–7.23 (m, 1 H, CH_Ar_).

***tert*-Butyl (2-chloro-2-oxido-4*H*-benzo[d]** **[1,3,2]** **dioxaphosphinin-6-yl)carbamate (11e)**

The general procedure **A** was applied to synthetize compound **11e** from saligenol **8e** (1 eq, 858 mg, 3.6 mmol). A ^31^P-NMR confirmed the presence of the product obtained as a yellow oil (proportion: 81%).

**^31^P NMR (161.9 MHz, chloroform-*d*):** δ −6.4.

**2-(But-3-yn-1-yloxy)-4H-benzo[*d*]** **[1,3,2]** **dioxaphosphinine 2-oxide (5a) (Supplementary Materials Figures S2 and S3)**

The general procedure with workup 1 was applied to synthetize compound **5a** from 3-butyn-1-ol **9** (1 eq, 0.5 mL, 6.4 mmol). The crude was purified by automated flash chromatography (from 70:30 PE/EtOAc to 50:50 PE/EtOAc). The desired product was afforded as a colorless solid (0.337 g, 22%).

Rf = 0.31 (60:40 PE/EtOAc).

**^1^H NMR (500 MHz, chloroform-*d*):** δ 1.96 (t, ^4^*J* = 2.7 Hz, 1H, CH), 2.61 (td, ^3^*J* = 6.8 Hz, ^4^*J* = 2.6 Hz, 2H, CCH_2_), 4.22–4.36 (m, 2H, CCH_2_C*H*_2_), 5.29–5.46 (m, 2H, CH_2_OP), 7.04–7.10 (m, 2H, CH_Ar_), 7.13 (td, *J* = 7.6 Hz, *J* = 1.9 Hz, 1H, CH_Ar_), 7.29–7.34 (m, 1H, CH_Ar_).

**^13^C NMR (125.8 MHz, chloroform-*d*):** δ_c_ 20.7 (d, ^3^*J*_C-P_ = 6.7 Hz, *C*H_2_CH_2_OP), 66.0 (d, ^2^*J*_C-P_ = 5.6 Hz, CH_2_*C*H_2_OP), 68.7 (d, ^2^*J*_C-P_ = 6.9 Hz, *C*H_2_OP), 70.6 (CH), 79.0 (*C*CH), 118.8 (d, *J*_C-P_ = 9.1 Hz, CH_Ar_), 120.6 (d, ^3^*J*_C-P_ = 9.9 Hz, C_Ar_), 124.3 (CH_Ar_), 125.3 (d, *J*_C-P_ = 1.1 Hz, CH_Ar_), 129.8 (d, *J*_C-P_ = 1.7 Hz, CH_Ar_), 150.2 (d, ^2^*J*_C-P_ = 7.0 Hz, OC_Ar_).

**^31^P NMR (121.5 MHz, chloroform-*d*):** δ −9.9.

**HRMS (EI)^+^:**  *m*/*z* calculated for C_11_H_12_O_4_P [M+H]^+^ 239.0468, found 239.0486.

**2-(But-3-yn-1-yloxy)-6-methoxy-4H-benzo[*d*]** **[1,3,2]** **dioxaphosphinine 2-oxide (5b) (Supplementary Materials Figures S3 and S4)**

The general procedure **B** with workup 1 was applied to synthetize compound **5b** from 3-butyn-1-ol **9** (1 eq, 0.1 mL, 1.34 mmol). The crude was purified by automated flash chromatography (from 60:40 PE/EtOAc to 50:50 PE/EtOAc). The desired product was afforded as a colorless oil (0.225 g, 62%). Rf = 0.41 (50:50 PE/EtOAc).

**^1^H NMR (300 MHz, chloroform-*d*):** δ 1.97 (t, ^4^*J* = 2.7 Hz, 1H, CH), 2.60 (td, ^3^*J* = 6.8 Hz, ^4^*J* = 2.7 Hz, 2H, CCH_2_), 3.77 (s, 3H, OCH_3_), 4.19–4.34 (m, 2H, CCH_2_C*H*_2_), 5.24–5.41 (m, 2H, CH_2_OP), 6.57 (d, *J* = 3.0 Hz, 1H, CH_Ar_), 6.79–6.87 (m, 1H, CH_Ar_), 6.99 (d, *J* = 8.9 Hz, 1H, CH_Ar_).

**^13^C NMR (125.8 MHz, chloroform-*d*):** δ_c_ 20.7 (d, ^3^*J*_C-P_ = 6.7 Hz, *C*H_2_CH_2_OP), 55.7 (OCH_3_), 65.9 (d, ^2^*J*_C-P_ = 5.6 Hz, CH_2_*C*H_2_OP), 68.7 (d, ^2^*J*_C-P_ = 7.0 Hz, *C*H_2_OP), 70.5 (CH), 79.0 (*C*CH), 110.1 (bs, CH_Ar_), 115.0 (d, *J*_C-P_ = 1.6 Hz, CH_Ar_), 119.6 (d, *J*_C-P_ = 9.1 Hz, CH_Ar_), 121.2 (d, ^3^*J*_C-P_ = 10.0 Hz, C_Ar_), 143.8 (d, ^2^*J*_C-P_ = 6.9 Hz, OC_Ar_), 156.0 (*C_Ar_*OCH_3_).

**^31^P NMR (121.5 MHz, chloroform-*d*):** δ −9.6.

**HRMS (EI)^+^:**  *m*/*z* calculated for C_12_H_14_O_5_P [M+H]^+^ 269.0573, found 269.0593.

**2-(But-3-yn-1-yloxy)-6-chloro-4H-benzo[*d*]** **[1,3,2]** **dioxaphosphinine 2-oxide (5c) (Supplementary Materials Figures S5 and S6)**

The general procedure **B** with workup 1 was applied to synthetize compound **5c** from 3-butyn-1-ol **9** (1 eq, 0.17 mL, 2.18 mmol). The crude was purified by automated flash chromatography (from 70:30 PE/EtOAc to 60:40 PE/EtOAc). The desired product was afforded as a colorless solid (0.439 g, 77%).

Rf = 0.24 (60:40 PE/EtOAc).

**^1^H NMR (300 MHz, chloroform-*d*):** δ 1.97 (t, ^4^*J* = 2.7 Hz, 1H, CH), 2.61 (td, ^3^*J* = 6.7 Hz, ^4^*J* = 2.7 Hz, 2H, CCH_2_), 4.20–4.37 (m, 2H, CCH_2_C*H*_2_), 5.22–5.45 (m, 2H, CH_2_OP), 7.00 (d, *J* = 8.9 Hz, 1H, CH_Ar_), 7.06–7.10 (m, 1H, CH_Ar_), 7.25–7.30 (m, 1H, CH_Ar_).

**^13^C NMR (125.8 MHz, chloroform-*d*):** δ_c_ 20.7 (d, ^3^*J*_C-P_ = 6.8 Hz, *C*H_2_CH_2_OP), 66.3 (d, ^2^*J*_C-P_ = 5.6 Hz, CH_2_*C*H_2_OP), 68.1 (d, ^2^*J*_C-P_ = 7.1 Hz, *C*H_2_OP), 70.6 (CH), 78.9 (*C*CH), 120.2 (d, *J*_C-P_ = 9.4 Hz, CH_Ar_), 119.6 (d, ^3^*J*_C-P_ = 9.9 Hz, C_Ar_), 125.2 (d, *J*_C-P_ = 1.1 Hz, CH_Ar_), 129.5 (C_Ar_), 129.8 (d, *J*_C-P_ = 1.5 Hz, CH_Ar_), 148.7 (d, ^2^*J*_C-P_ = 6.9 Hz, OC_Ar_).

**^31^P NMR (121.5 MHz, chloroform-*d*):** δ −10.3.

**HRMS (EI)^+^:**  *m*/*z* calculated for C_11_H_10_ClKO_4_P [M+K]^+^ 310.9637, found 310.9647.

**2-(But-3-yn-1-yloxy)-6-(trifluoromethyl)-4H-benzo[*d*]** **[1,3,2]** **dioxaphosphinine 2-oxide (5d) (Supplementary Materials Figures S6–S8)**

The general procedure **B** with workup 1 was applied to synthetize compound **5d** from 3-butyn-1-ol **9** (1 eq, 0.013 mL, 0.17 mmol). The crude was purified by automated flash chromatography (from 70:30 PE/EtOAc to 60:40 PE/EtOAc). The desired product was afforded as a colorless oil (0.047 g, 90%).

Rf = 0.38 (70:30 PE/EtOAc).

**^1^H NMR (300 MHz, chloroform-*d*):** δ 1.96 (t, ^4^*J* = 2.6 Hz, 1H, CH), 2.62 (td, ^3^*J* = 6.7 Hz, ^4^*J* = 2.6 Hz, 2H, CCH_2_), 4.42–4.19 (m, 2H, CCH_2_C*H*_2_), 5.54–5.29 (m, 2H, CH_2_OP), 7.16 (d, *J* = 8.6 Hz, 1H, CH_Ar_), 7.41–7.34 (m, 1H, CH_Ar_), 7.59 (d, *J* = 8.5 Hz, 1H, CH_Ar_).

**^13^C NMR (125.8 MHz, chloroform-*d*):** δ_c_ 20.7 (d, ^3^*J*_C-P_ = 6.7 Hz, *C*H_2_CH_2_OP), 66.5 (d, ^2^*J*_C-P_ = 5.7 Hz, CH_2_*C*H_2_OP), 68.2 (d, ^2^*J*_C-P_ = 7.1 Hz, *C*H_2_OP), 70.7 (CH), 78.8 (*C*CH), 119.4 (d, *J*_C-P_ = 9.3 Hz, CH_Ar_), 121.1 (d, ^3^*J*_C-P_ = 10.1 Hz, C_Ar_), 122.9 (q, ^3^*J*_C-F_ = 3.7 Hz, CH_Ar_), 123.5 (q, ^1^*J*_C-F_ = 271.9 Hz, CF_3_), 126.8 (q, ^2^*J*_C-F_ = 33.7 Hz, *C_Ar_*CF_3_), 127.0–127.2 (m, CH_Ar_), 152.6 (bdd, ^2^*J*_C-P_ = 6.7, ^5^*J*_C-F_ = 1.5 Hz, OC_Ar_).

**^31^P NMR (121.5 MHz, chloroform-*d*):** δ −10.7.

**^19^F NMR (282.4 MHz, chloroform-*d*):** δ_F_ −62.2.

**HRMS (EI)^+^:**  *m*/*z* calculated for C_12_H_11_F_3_O_4_P [M+H]^+^ 307.0342, found 307.0368.

***tert*-Butyl-(2-(but-3-yn-1-yloxy)-2-oxido-4*H*-benzo[*d*][1,3,2]dioxaphosphinin-6-yl)carbamate (5e) (** **Supplementary Materials Figures S8 and S9** **)**

The general procedure **B** with workup 1 was applied to synthetize compound **5e** from 3-butyn-1-ol **9** (1 eq, 75 µL, 0.98 mmol). The crude was purified by automated flash chromatography (from 80:20 PE/EtOAc to 50:50 PE/EtOAc). The desired product was afforded as a colorless oil (213 mg, 61%).

**^1^H NMR (500 MHz, chloroform-*d*):** δ = 1.51 (s, 9H, C(C*H*_3_)_3_), 1.97 (t, *J* = 2.7 Hz, 1H, C*H*), 2.60 (td, *J* = 6.8, 2.7 Hz, 2H, CC*H*_2_), 4.19–4.34 (m, 2H CCH_2_C*H*_2_), 5.25–5.42 (m, 2H, C*H*_2_OP), 6.50 (s, 1H, N*H*), 6.97 (d, *J* = 8.8 Hz, 1H, CH_Ar_), 7.02–7.07 (m, 1H, CH_Ar_), 7.41 (s, 1H, CH_Ar_).

**^13^C NMR (125.8 MHz, chloroform-*d*):** δ = 20.8 (d, *J* = 6.8 Hz, *C*H_2_CH_2_OP), 28.4 (C(*C*H_3_)_3_), 66.1 (d, *J* = 5.6 Hz, CH_2_*C*H_2_OP), 68.9 (d, *J* = 7.0 Hz, CH_2_OP), 70.7 (C*C*H), 79.1 (*C*CH), 81.2 (*C*(CH_3_)_3_), 115.2 (CH_Ar_), 119.2 (d, *J* = 9.2 Hz, CH_Ar_), 119.8 (CH_Ar_), 121.1 (d, *J* = 9.9 Hz, CH_2_-*C*_Ar_), 134.9 (N-C_Ar_), 145.6 (d, *J* = 6.9 Hz, O-C_Ar_), 152.9 (CO).

**^31^P NMR (202 MHz, chloroform-*d*):** δ −9.7.

**HRMS (EI)^+^:**  *m*/*z* calculated for C_16_H_20_NaNO_6_P [M+Na]^+^ 376.0920, found 376.0917.

**2-(But-3-yn-1-yloxy)-4*H*-benzo[d][1,3,2]dioxaphosphinin-6-aminium-2-oxide trifluoroacetate (5f) (** **Supplementary Materials Figures S10 and S11** **)**

To a solution of compound **5e** (86 mg, 0.24 mmol) in dry DCM (2.5 mL) at 0 °C was added trifluoroacetic acid (0.5 mL). The resulting reaction mixture was stirred at the same temperature for 1.5h. After reaction completion, volatiles were removed under vacuo to give the desired compound as a brownish oil (88 mg, quant. yield).

**^1^H NMR (500 MHz, methanol-*d*_4_):** δ = 2.31 (t, *J* = 2.7 Hz, 1H, C*H*), 2.61 (dddd, J = 6.6, 6.2, 2.7, 0.9 Hz, 2H, CC*H*_2_), 4.20–4.33 (m, 2H, CCH_2_C*H*_2_), 5.42–5.56 (m, 2H, C*H*_2_OP), 7.12–7.16 (m, 1H, CH_Ar_), 7.18–7.22 (m, 1H, CH_Ar_), 7.24–7.28 (m, 1H, CH_Ar_).

**^13^C NMR (125.8 MHz, methanol-*d*_4_):** δ 21.2 (d, *J* = 6.9 Hz, *C*H_2_CH_2_OP), 68.1 (d, *J* = 5.9 Hz, CH_2_*C*H_2_OP), 69.8 (d, *J* = 7.0 Hz, CH_2_OP), 71.7 (C*C*H), 80.1 (*C*CH), 117.6 (q, *J* = 290.1 Hz, CF_3_*C*OO^−^), 119.9 (CH_Ar_), 121.1 (d, *J* = 9.2 Hz, CH_Ar_), 123.9 (d, *J* = 9.9 Hz C*H*_2_-*C*_Ar_), 124.0 (CH_Ar_), 132.3 (N-C_Ar_), 149.5 (d, *J* = 6.5 Hz, O-C_Ar_), 162.0 (q, *J* = 35.2 Hz, CF_3_*C*OO^−^).

**^31^P NMR (202 MHz, methanol-*d*_4_):** δ −9.7.

**^19^F NMR (470 MHz, methanol-*d*_4_):** δ −77.2.

**HRMS (EI)^+^:**  *m*/*z* calculated for C_11_H_13_NO_4_P [M+H]^+^ 254.0577, found 254.0578.

**2-(Pyridin-3-yl)ethan-1-ol (10)**

To a solution of ethyl 2-(pyridin-3-yl)acetate (1 eq, 0.46 mL, 3.0 mmol) in THF (6 mL/mmol, 18 mL) cooled down to 0 °C, a 1 M solution of LiAlH_4_ in THF (1.1 eq, 3.3 mL) was added dropwise and the reaction mixture was then stirred at room temperature for 3h. After completion, the reaction mixture was cooled down to 0 °C, quenched with a saturated solution of Seignette’s salt and allowed to warm to room temperature for 30 min. Then, Et_2_O (10 mL) was added, and after separation of the layers, the aqueous one was extracted with EtOAc (3 × 10 mL). The combined organic layers were dried over Na_2_SO_4_ and evaporated. The crude was purified by automated flash chromatography (from 100% EtOAc to 90:10 EtOAc/MeOH). The desired product was afforded as a yellowish oil (0.327 g, 88%).

Rf = 0.24 (90:10 EtOAc/MeOH).

**^1^H NMR (300 MHz, chloroform-*d*):** δ 2.67 (bs, 1H, OH), 2.85 (t, ^3^*J* = 6.5 Hz, 2H, C*H*_2_CH_2_OH), 3.87 (t, ^3^*J* = 6.5 Hz, 2H, C*H*_2_OH), 7.21 (dd, ^3^*J* = 7.7 Hz, ^3^*J* = 5.0 Hz, 1H, CH_Ar_), 7.56 (d-*pseudo*-t, ^3^*J* = 7.8 Hz, ^4^*J* = 1.9 Hz, 1H, CH_Ar_), 8.32–8.47 (m, 2H, CH_Ar_).

**^13^C NMR (125.8 MHz, chloroform-*d*):** δ_c_ 36.4 (*C*H_2_CH_2_OH), 62.9 (CH_2_OH), 123.4 (CH_Ar_), 134.7 (C_Ar_), 136.8 (CH_Ar_), 147.5 (CH_Ar_), 150.1 (CH_Ar_).

**2-(2-(Pyridin-3-yl)ethoxy)-4H-benzo[*d*]** **[1,3,2]** **dioxaphosphinine 2-oxide (6a) (Supplementary Materials Figures S16 and S17)**

The general procedure **B** with workup 2 was applied to synthetize compound **6a** from 2-(pyridin-3-yl)ethan-1-ol **10** (1 eq, 0.205 g, 1.66 mmol). The crude was purified by automated flash chromatography (from 98:2 EtOAc/MeOH to 95:5 EtOAc/MeOH). The desired product was afforded as a colorless oil (0.107 g, 22%).

Rf = 0.29 (95:5 EtOAc/MeOH).

**^1^H NMR (300 MHz, chloroform-*d*):** δ 3.02 (t, ^3^*J* = 6.5 Hz, 2H, CH_2_pyr), 4.32–4.49 (m, 2H, C*H*_2_CH_2_pyr), 5.12–5.38 (m, 2H, CH_2_OP), 6.96–7.06 (m, 2H, CH_Ar-*cyclo*Sal_), 7.11 (*pseudo*-td, ^3^*J* = 7.4 Hz, ^4^*J* = 1.1 Hz, 1H, CH_Ar-*cyclo*Sal_), 7.17 (ddd, *J* = 7.8, 4.8, 0.8 Hz, 1H, CH_Ar-pyr_), 7.26–7.34 (m, 1H, CH_Ar-*cyclo*Sal_), 7.46–7.54 (m, 1H, CH_Ar-pyr_), 8.42 (d, *J* = 2.2 Hz, 1H, CH_Ar-pyr_), 8.46 (dd, *J* = 4.8 Hz, *J* = 2.2 Hz, 1H, CH_Ar-pyr_).

**^13^C NMR (125.8 MHz, chloroform-*d*):** δ_c_ 33.9 (d, ^3^*J*_C-P_ = 6.5 Hz, CH_2_pyr), 68.2 (d, ^2^*J*_C-P_ = 5.9 Hz, CH_2_OP), 68.6 (d, ^2^*J*_C-P_ = 7.1 Hz, *C*H_2_CH_2_pyr), 118.7 (d, *J*_C-P_ = 8.9 Hz, CH_Ar-*cyclo*Sal_), 120.5 (d, ^3^*J*_C-P_ = 10.0 Hz, C_Ar-*cyclo*Sal_), 123.4 (CH_Ar-pyr_), 124.3 (CH_Ar-pyr_), 125.3 (d, *J*_C-P_ = 1.0 Hz, CH_Ar-*cyclo*Sal_), 129.8 (d, *J*_C-P_ = 1.8 Hz, CH_Ar-*cyclo*Sal_), 132.4 (C_Ar-pyr_), 136.4 (CH_Ar-pyr_), 148.4 (CH_Ar-pyr_), 150.0 (d, ^2^*J*_C-P_ = 6.9 Hz, OC_Ar-*cyclo*Sal_), 153.3 (CH_Ar-*cyclo*Sal_).

**^31^P NMR (121.5 MHz, chloroform-*d*):** δ −9.8.

**HRMS (EI)^+^:**  *m*/*z* calculated for C_14_H_15_NO_4_P [M+H]^+^ 292.0733, found 292.0735.

**6-Methoxy-2-(2-(pyridin-3-yl)ethoxy)-4H-benzo[d]** **[1,3,2]** **dioxaphosphinine 2-oxide (6b) (Supplementary Materials Figures S17 and S18)**

The general procedure **B** with workup 2 was applied to synthetize compound **6b** from 2-(pyridin-3-yl)ethan-1-ol **10** (1 eq, 0.084 g, 0.68 mmol). The crude was purified by automated flash chromatography (from 100% EtOAc to 95:5 EtOAc/MeOH). The desired product was afforded as a colorless oil (0.057 g, 23%).

Rf = 0.25 (95:5 EtOAc/MeOH).

**^1^H NMR (500 MHz, chloroform-*d*):** δ 2.99 (t, ^3^*J* = 6.5 Hz, 2H, CH_2_pyr), 3.75 (s, 3H, OCH_3_), 4.28–4.46 (m, 2H, C*H*_2_CH_2_pyr), 5.06–5.30 (m, 2H, CH_2_OP), 6.51 (d, *J* = 2.9 Hz, 1H, CH_Ar-*cyclo*Sal_), 6.76–6.82 (m, 1H, CH_Ar-*cyclo*Sal_), 6.87–6.93 (m, 1H, CH_Ar-pyr_), 7.13–7.20 (m, 2H, CH_Ar-*cyclo*Sal_), 7.48 (dt, ^3^*J* = 7.8 Hz, ^4^*J* = 1.9 Hz, 1H, CH_Ar-pyr_), 8.41 (d, *J* = 2.1 Hz, 1H, CH_Ar-pyr_), 8.45 (dd, *J* = 4.9 Hz, *J* = 1.5 Hz, 1H, CH_Ar-pyr_).

**^13^C NMR (125.8 MHz, chloroform-*d*):** δ_c_ 33.9 (d, ^3^*J*_C-P_ = 6.4 Hz, CH_2_pyr), 55.6 (OCH_3_), 68.1 (d, ^2^*J*_C-P_ = 6.9 Hz, CH_2_OP), 68.6 (d, ^2^*J*_C-P_ = 7.1 Hz, *C*H_2_CH_2_pyr), 110.1 (bs, CH_Ar-*cyclo*Sal_), 115.0 (d, *J*_C-P_ = 1.6 Hz, CH_Ar-*cyclo*Sal_), 119.5 (d, *J*_C-P_ = 9.1 Hz, CH_Ar-*cyclo*Sal_), 121.1 (d, ^3^*J*_C-P_ = 10.0 Hz, C_Ar-*cyclo*Sal_), 123.4 (CH_Ar-pyr_), 132.4 (C_Ar-pyr_), 136.4 (CH_Ar-pyr_), 143.6 (d, ^2^*J*_C-P_ = 7.0 Hz, OC_Ar-*cyclo*Sal_), 148.3 (CH_Ar-pyr_), 150.3 (CH_Ar-pyr_), 156.0 (C_Ar-*cyclo*Sal_OCH_3_).

**^31^P NMR (121.5 MHz, chloroform-*d*):** δ −9.6.

**HRMS (EI)^+^:**  *m*/*z* calculated for C_15_H_17_NO_5_P [M+H]^+^ 322.0838, found 322.0845.

**6-Chloro-2-(2-(pyridin-3-yl)ethoxy)-4H-benzo[*d*]** **[1,3,2]** **dioxaphosphinine 2-oxide (6c) (Supplementary Materials Figures S19 and S20)**

The general procedure **B** with workup 2 was applied to synthetize compound **6c** from 2-(pyridin-3-yl)ethan-1-ol **10** (1 eq, 0.170 g, 1.38 mmol). The crude was purified by automated flash chromatography (from 100% EtOAc to 95:5 EtOAc/MeOH). The desired product was afforded as a colorless oil (0.162 g, 36%).

Rf = 0.59 (95:5 EtOAc/MeOH).

**^1^H NMR (300 MHz, chloroform-*d*):** δ 3.01 (t, ^3^*J* = 6.5 Hz, 2H, CH_2_pyr), 4.31–4.49 (m, 2H, C*H*_2_CH_2_pyr), 5.10–5.30 (m, 2H, CH_2_OP), 6.91 (d, ^3^*J* = 8.8 Hz, 1H, CH_Ar-*cyclo*Sal_), 7.00 (d, ^4^*J* = 2.5 Hz, 1H, CH_Ar-pyr_), 7.14–7.26 (m, 2H, CH_Ar-*cyclo*Sal_), 7.49 (dt, ^3^*J* = 7.8 Hz, ^4^*J* = 1.9 Hz, 1H, CH_Ar-pyr_), 8.42 (d, *J* = 2.2 Hz, 1H, CH_Ar-pyr_), 8.46 (dd, *J* = 4.9 Hz, *J* = 1.6 Hz, 1H, CH_Ar-pyr_).

**^13^C NMR (125.8 MHz, chloroform-*d*):** δ_c_ 33.8 (d, ^3^*J*_C-P_ = 6.6 Hz, CH_2_pyr), 68.0 (d, ^2^*J*_C-P_ = 7.0 Hz, CH_2_OP), 68.4 (d, ^2^*J*_C-P_ = 5.9 Hz, *C*H_2_CH_2_pyr), 120.1 (d, *J*_C-P_ = 9.2 Hz, CH_Ar-*cyclo*Sal_), 121.9 (d, ^3^*J*_C-P_ = 10.1 Hz, C_Ar-*cyclo*Sal_), 123.5 (CH_Ar-pyr_), 125.3 (d, *J*_C-P_ = 1.0 Hz, CH_Ar-*cyclo*Sal_), 129.6 (C_Ar-*cyclo*Sal_), 129.8 (d, *J*_C-P_ = 1.5 Hz, CH_Ar-*cyclo*Sal_), 132.3 (C_Ar-pyr_), 136.4 (CH_Ar-pyr_), 148.3 (CH_Ar-pyr_), 148.5 (d, ^2^*J*_C-P_ = 6.9 Hz, OC_Ar-*cyclo*Sal_), 150.2 (CH_Ar-pyr_).

**^31^P NMR (121.5 MHz, chloroform-*d*):** δ −10.4.

**HRMS (EI)^+^:**  *m*/*z* calculated for C_14_H_14_ClNO_4_P [M+H]^+^ 326.0343, found 326.0348.

**2-(2-(Pyridin-3-yl)ethoxy)-6-(trifluoromethyl)-4*H*-benzo[*d*]** **[1,3,2]** **dioxaphosphinine 2-oxide (6d) (Supplementary Materials Figures S20–S22)**

The general procedure **B** with workup 2 was applied to synthetize compound **6d** from 2-(pyridin-3-yl)ethan-1-ol **10** (1 eq, 90 mg, 0.73 mmol). The crude was purified by automated flash chromatography (from 100% EtOAc to 95:5 EtOAc/MeOH). The desired product was afforded as a colorless oil (10.9 mg, 4%).

**^1^H NMR (400 MHz, chloroform-*d*):** δ 3.04 (t, *J* = 6.5 Hz, 2H, CH_2_-C_pyr_), 4.36–4.56 (m, 2H, C*H*_2_CH_2_-C_pyr_), 5.18–5.38 (m, 2H, CH_2_OP), 7.09 (d, *J* = 8.6 Hz, 1H, CH_Ar-*cyclo*Sal_), 7.18 (dd, *J* = 7.8, 4.8 Hz, 1H, CH_Ar-pyr_), 7.32 (s, 1H, CH_Ar-*cyclo*Sal_), 7.48–7.54 (m, 1H), 7.57 (d, *J* = 8.7 Hz, 1H, CH_Ar-*cyclo*Sal_), 8.42–8.49 (m, 2H, CH_Ar-pyr_).

**^13^C NMR (125.8 MHz, chloroform-*d*):** δ 34.0 (d, *J* = 6.5 Hz, *C*H_2_-C_pyr_), 68.2 (d, *J* = 7.1 Hz, CH_2_OP), 68.7 (d, *J* = 5.8 Hz, *C*H_2_CH_2_-Cpyr), 119.5 (d, *J* = 9.2 Hz, CH_Ar-*cyclo*Sal_), 121.1 (d, *J* = 10.1 Hz, C_Ar-*cyclo*Sal_), 123.0 (qd, *J* = 3.7, 1.0 Hz, CH_Ar-*cyclo*Sal_), 123.55 (q, *J* = 272.0 Hz, CF_3_), 123.59 (CH_Ar-pyr_), 127.0 (q, *J* = 33.6 Hz, *C*_Ar-*cyclo*Sal_-CF3), 127.2–127.4 (m, CH_Ar-*cyclo*Sal_), 132.3 (C_Ar-pyr_), 136.5 (CH_Ar-pyr_), 148.5 (CH_Ar-pyr_), 150.3 (CH_Ar-pyr_), 152.5 (dq, *J* = 6.9, 1.2 Hz, OC_Ar-*cyclo*Sal_).

**^31^P NMR (162 MHz, chloroform-*d*):** δ −10.65.

**^19^F NMR (376 MHz, chloroform-*d*):** δ −62.2.

**HRMS (EI)^+^:**  *m*/*z* calculated for C_15_H_14_F_3_NO_4_P [M+H]^+^ 360.0607, found 360.0606.

**Methyl (2*S*)-2-((*tert*-butoxycarbonyl)amino)-3-(2-oxido-2-(2-(pyridin-3-yl)ethoxy)-4*H*-benzo[*d*][1,3,2]dioxaphosphinin-6-yl)propanoate (6g) (** **Supplementary Materials Figures S22–S24** **)**

The general procedure **B** with workup 2 was applied to synthetize compound **6d** from 2-(pyridin-3-yl)ethan-1-ol **10** (1 eq, 92 mg, 0.75 mmol). The crude was purified by flash chromatography (95:5 EtOAc/MeOH). The desired product was afforded as a colorless oil (83.2 mg, 22%). Two diastereoisomers were detected by NMR spectroscopy with a diastereomeric ratio of 66:33. Unambiguous assignment of the signals to each individual diastereomer has not been achieved.

**^1^H NMR (500 MHz, chloroform-*d*):** δ = 1.41 (s, 2.96H, C(CH_3_)_3_, minor diastereoisomer), 1.42 (s, 6.02H, C(CH_3_)_3_, major diastereoisomer), 2.94–3.14 (m, 4H, CH_2_-C_pyr_ and C*H*_2_CH), 3.718 (s, 1.9H, OCH_3_, minor diastereoisomer), 3.722 (s, 1.1H, OCH_3_, major diastereoisomer), 4.34–4.46 (m, 2H, C*H*_2_CH_2_-C_pyr_), 4.51–4.63 (m, 1H, NCH), 4.99–5.30 (m, 2H, CH_2_OP), 5.36 (d, *J* = 8.3 Hz, 0.36H, NH, minor diastereoisomer), 5.57 (d, *J* = 8.3 Hz, 0,69H, NH, major diastereoisomer), 6.77–6.83 (m, 1H, CH_Ar-*cyclo*Sal_), 6.87 (d, *J* = 8.4 Hz, 0.35H, CH_Ar-*cyclo*Sal_), 6.91 (d, *J* = 8.4 Hz, 0.68H, CH_Ar-*cyclo*Sal_), 7.00–7.07 (m, 1H, CH_Ar-*cyclo*Sal_), 7.20 (dd, *J* = 7.8, 4.8 Hz, 1H, CH_Ar-pyr_), 7.48–7.54 (m, 1H, CH_Ar-pyr_), 8.26 (d, *J* = 2.3 Hz, 0.66H, CH_Ar-pyr_), 8.33 (d, *J* = 2.3 Hz, 0.33H, CH_Ar-pyr_), 8.45–8.51 (m, 1H, CH_Ar-pyr_).

**^13^C NMR (125.8 MHz, chloroform-*d*):** 28.4 (C(*C*H_3_)_3_, minor diastereoisomer), 28.5 (C(*C*H_3_)_3_, major diastereoisomer), 33.96 (d, *J* = 6.4 Hz, *C*H_2_-C_pyr_, major diastereoisomer), 33.98 (d, *J* = 6.4 Hz, *C*H_2_-C_pyr_, minor diastereoisomer), 37.66 (*C*H_2_CHN, minor diastereoisomer), 37.70 (*C*H_2_CHN, major diastereoisomer), 52.47 (OCH_3_, major diastereoisomer), 52.50 (OCH_3_, minor diastereoisomer), 54.48 (CHN, minor diastereoisomer), 54.53 (CHN, major diastereoisomer), 68.2 (d, *J* = 5.8 Hz, *C*H_2_OP, minor diastereoisomer), 68.4 (d, *J* = 5.8 Hz, *C*H_2_OP, major diastereoisomer), 68.7 (d, *J* = 7.0 Hz, *C*H_2_CH_2_-Cpyr, minor diastereoisomer), 68.71 (d, *J* = 7.0 Hz, *C*H_2_CH_2_-Cpyr, major diastereoisomer), 80.17 (*C*(CH_3_)_3_, major diastereoisomer), 80.21 (C(CH_3_)_3_, minor diastereoisomer), 118.82 (d, *J* = 8.8 Hz, CH_Ar-*cyclo*Sal_, minor diastereomer), 118.85 (d, *J* = 9.0 Hz, CH_Ar-*cyclo*Sal_, major diastereoisomer), 120.49 (d, *J* = 10.1 Hz C_Ar-*cyclo*Sal_, major diastereomer), 120.55 (d, *J* = 10.0 Hz, C_Ar-*cyclo*Sal_, minor diastereomer), 123.5 (CH_Ar-pyr_, major diastereomer), 123.6 (CH_Ar-pyr_, minor diastereomer), 126.0 (CH_Ar-*cyclo*Sal_, minor diastereomer), 126.2 (CH_Ar-*cyclo*Sal_, major diastereomer), 130.7 (d, *J* = 1.7 Hz, CH_Ar-*cyclo*Sal_, major diastereomer), 130.9 (d, *J* = 1.6 Hz, CH_Ar-*cyclo*Sal_, minor diastereomer), 132.56 (C_Ar-pyr_, major diastereomer), 132.57 (C_Ar-pyr_, minor diastereomer), 132.7 (C_Ar-*cyclo*Sal_, minor diastereomer), 132.8 (C_Ar-*cyclo*Sal_, major diastereomer), 136.49 (CH_Ar-pyr_, major diastereomer), 136.50 (CH_Ar-pyr_, minor diastereomer), 148.4 (CH_Ar-pyr_), 149.1 (d, *J* = 6.9 Hz, O-C_Ar,_ major diastereomer), 149.1 (d, *J* = 7.0 Hz, O-C_Ar,_ minor diastereomer), 150.26 (CH_Ar-pyr_, major diastereomer), 150.27 (CH_Ar-pyr_, minor diastereomer), 155.26 (NCO, minor diastereomer), 155.33 (NCO, major diastereomer), 172.12 (CO, major diastereomer), 172.14 (CO, minor diastereomer).

**^31^P NMR (162 MHz, chloroform-*d*):** δ −9.9 (minor diastereoisomer), −10.1 (major diastereoisomer).

**HRMS (ESI, positive mode)** *m*/*z* calculated for C_23_H_30_O_8_N_2_P [M+H]^+^ 493.1734, found 493.1738.

**(2*S*)-1-methoxy-3-(2-oxido-2-(2-(pyridin-3-yl)ethoxy)-4H-benzo[*d*][1,3,2]dioxaphosphinin-6-yl)-1-oxopropan-2-aminium 2,2,2-trifluoroacetate (6h) (** **Supplementary Materials Figures S24–S26** **)**

To a solution of compound **6g** (41.8 mg, 0.085 mmol) in dry DCM (1 mL) at 0 °C, trifluoroacetic acid (0.5 mL) was added. The resulting reaction mixture was stirred at the same temperature for 1h. After reaction completion, volatiles were removed under vacuo to give the desired compound as a light-brown paste (46.9 mg, 90%). Two diastereoisomers were detected by NMR spectroscopy with a diastereomeric ratio of 66:33. Unambiguous assignment of the signals to each individual diastereoisomer has not been achieved.

**^1^H NMR (400 MHz, methanol-*d*_4_)**: δ 3.10–3.31 (m, 4H, CH_2_-C_pyr_ and C*H*_2_CH), 3.82 (s, 3H, OCH_3_), 4.29–4.37 (m, 1H, NCH), 4.45–4.62 (m, 2H, C*H*_2_CH_2_-C_pyr_), 5.37–5.44 (m, 2H, CH_2_OP), 6.99–7.08 (m, 1H, CH_Ar-*cyclo*Sal_), 7.08–7.15 (m, 1H, CH_Ar-*cyclo*Sal_), 7.21–7.30 (m, 1H, CH_Ar-*cyclo*Sal_), 7.99 (dd, *J* = 8.2, 5.8 Hz, 1H, CH_Ar-pyr_), 8.48–8.55 (m, 1H, CH_Ar-pyr_), 8.73 (d, *J* = 5.7 Hz, 1H, CH_Ar-pyr_), 8.78 (d, *J* = 2.2 Hz, 1H, CH_Ar-pyr_).

**^13^C NMR (125.8 MHz, methanol-*d*_4_):** δ 34.3 (d, *J* = 6.6 Hz, *C*H_2_-C_pyr_), 36.51 (*C*H_2_CHN, minor diastereoisomer), 36.53 (CH_2_CHN, major diastereoisomer), 53.8 (OCH_3_), 55.0 (CHN), 69.1 (d, *J* = 5.8 Hz, *C*H_2_CH_2_-Cpyr), 70.1 (d, *J* = 6.9 Hz, *C*H_2_OP), 120.1 (d, *J* = 9.0 Hz, CH_Ar-*cyclo*Sal_, minor diastereoisomer), 120.2 (d, *J* = 9.0 Hz, CH_Ar-*cyclo*Sal_, major diastereoisomer), 122.7 (d, *J* = 10.0 Hz, C_Ar-*cyclo*Sal_, major diastereomer), 122.8 (d, *J* = 10.0 Hz, C_Ar-*cyclo*Sal_, minor diastereomer), 127.97 (CH_Ar-pyr_), 128.01 (d, *J* = 1.1 Hz, CH_Ar-*cyclo*Sal_, minor diastereomer), 128.1 (d, *J* = 1.1 Hz, CH_Ar-*cyclo*Sal_, major diastereomer), 132.16 (d, *J* = 1.7 Hz, CH_Ar-*cyclo*Sal_, major diastereomer), 132.22 (d, *J* = 1.7 Hz, CH_Ar-*cyclo*Sal_, minor diastereomer), 132.3 (C_Ar-*cyclo*Sal_, minor diastereomer), 132.4 (C_Ar-*cyclo*Sal_, minor diastereomer), 139.3 (C_Ar-pyr_), 142.4 (CH_Ar-pyr_), 144.3 (CH_Ar-pyr_), 147.3 (CH_Ar-pyr_), 150.7 (d, *J* = 6.7 Hz, O-C_Ar_), 170.29 (CO, minor diastereomer), 170.31 (CO, major diastereomer).

**^31^P NMR (161.9 MHz, methanol-*d*_4_)**: δ −9.49 (major diastereoisomer), −9.52 (minor diastereoisomer).

**HRMS (ESI, positive mode)** *m*/*z* calculated for C_18_H_22_O_6_N_2_P [M+H]^+^ 393.1210, found 393.1203.

**2-((*tert*-Butoxycarbonyl)amino)-3-(4-hydroxy-3-(hydroxymethyl)phenyl)propanoic acid (13)**

To a solution of *N*-(*tert*-butoxycarbonyl)-L-tyrosine methyl ester (1 eq, 2.200 g, 7.45 mmol) and sodium tetraborate decahydrate (borax) (2 eq, 5.721 g, 14.9 mmol) in water (2.4 mL/mmol, 18 mL) was added a 1M solution of sodium hydroxide (2.3 mL/mmol, 17 mL). The reaction mixture was stirred at room temperature for 30 min, then aqueous formaldehyde (4 eq, 2.23 mL of a 37% solution, 29.8 mmol) was added and the reaction was stirred at 40 °C for 5 days. After completion, the reaction mixture was allowed to cool down to room temperature and acidified with a 10% HCl solution to pH 2. The suspension was extracted with EtOAc (3 × 20 mL), and the combined organic layers were washed with water (20 mL), brine (20 mL), dried over Na_2_SO_4_ and evaporated. The desired product (1.942 g) was used in the next step without further purification.

**^1^H NMR (300 MHz, DMSO-*d*_6_):** δ 1.33 (s, 9H, C(CH_3_)_3_), 2.70 (dd, ^3^*J* = 13.7 Hz, 10.2 Hz, 1H, C*H*_2_CH), 2.88 (dd, ^3^*J* = 13.9 Hz, 4.5 Hz, 1H, C*H*_2_CH), 3.96–4.05 (m, 1H, CH), 4.44 (s, 2H, C*H*_2_OH), 4.94 (bt, ^3^*J* = 5.3 Hz, 1H, NH), 6.64 (d, ^3^*J* = 8.1 Hz, 1H, CH_Ar_), 6.90 (d, ^3^*J* = 8.2 Hz, 2.2 Hz, 1H, CH_Ar_), 6.97 (d, ^3^*J* = 8.2 Hz, 1H, CH_Ar_), 7.15 (s, 1H, PhOH).

**Methyl 2-((*tert*-butoxycarbonyl)amino)-3-(4-hydroxy-3-(hydroxymethyl)phenyl) propanoate (14)**

A solution of NaHCO_3_ (2 eq, 1.017 g, 12 mmol) and carboxylic acid **13** (1 eq, 1.879 g, 6.0 mmol) in DMF (3 mL/mmol, 18 mL) was stirred at room temperature for 30 min prior to dropwise addition of methyl iodide (2 eq, 0.75 mL, 12 mmol), and the reaction mixture was stirred overnight at room temperature. After completion, water (30 mL) was added, and the reaction mixture was extracted with EtOAc (3 × 15 mL). The combined organic layers were washed with water (2 × 15 mL), brine (2 × 15 mL), dried over Na_2_SO_4_ and evaporated. The crude was purified by automated flash chromatography (from 60:40 PE/EtOAc to 50:50 PE/EtOAc). The desired product was afforded as a colorless solid (1.413 g, 69%).

Rf = 0.22 (60:40 PE/EtOAc).

**^1^H NMR (300 MHz, chloroform-*d*)**: δ 1.41 (s, 9H, C(CH_3_)_3_), 2.77–3.06 (m, 3H, CH and C*H*_2_CH), 3.71 (s, 3H, OCH_3_), 3.96–4.05 (m, 1H, CH), 4.49 (*pseudo*-q, ^3^*J* = 7.4 Hz, 1H, CH_2_O*H*), 4.75 (bs, 2H, C*H*_2_OH), 5.01 (d, ^3^*J* = 8.2 Hz, NH), 6.72–6.83 (m, 2H, CH_Ar_), 6.87–6.95 (m, 1H, CH_Ar_), 7.54 (bs, 1H, PhOH).

**^13^C NMR (125.8 MHz, chloroform-*d*)**: δ_c_ 28.3 (C(*C*H_3_)_3_), 37.5 (*C*H_2_CHN), 52.3 (OCH_3_), 54.7 (CH), 64.1 (*C*H_2_OH), 80.2 (*C*(CH_3_)_3_), 116.5 (CH_Ar_), 125.2 (C_Ar_), 127.3 (C_Ar_), 128.7 (CH_Ar_), 130.0 (CH_Ar_), 155.1 (C_Ar_), 155.3 (NCO), 172.6 (CO).

**Methyl 2-((*tert*-butoxycarbonyl)amino)-3-(2-chloro-2-oxido-4H-benzo[d]** **[1,3,2]** **dioxaphosphinin-6-yl)propanoate (15)**

The general procedure **A** was applied to synthetize compound **15** from saligenol **14** (1 eq, 0.939 g, 2.9 mmol). A ^31^P-NMR confirmed the presence of the product obtained as a colorless solid (proportion: 71%).

Rf = 0.72 (30:70 PE/EtOAc).

**^31^P NMR (161.9 MHz, chloroform-*d*):** δ −6.0.

**Methyl 3-(2-(but-3-yn-1-yloxy)-2-oxido-4H-benzo[d]** **[1,3,2]** **dioxaphosphinin-6-yl)-2-((*tert*-butoxycarbonyl)amino)propanoate (5g) (Supplementary Materials Figures S12 and S13)**

The general procedure with workup 1 was applied to synthetize compound **5g** from 3-butyn-1-ol **9** (1 eq, 0.1 mL, 1.34 mmol). The crude was purified by automated flash chromatography (from 60:40 PE/EtOAc to 40:60 PE/EtOAc). The desired product was afforded as a colorless oil (0.265 g, 45%).

Rf = 0.34 (50:50 PE/EtOAc).

**^1^H NMR (300 MHz, chloroform-*d*):** δ 1.41 (s, 9H, C(CH_3_)_3_), 1.97 (t, ^4^*J* = 2.7 Hz, 1H, CCH), 2.62 (td, ^3^*J* = 6.8 Hz, ^4^*J* = 2.7 Hz, 1H, CCH_2_), 2.97 (dd, ^3^*J* = 14.0 Hz, ^3^*J* = 6.5 Hz, 1H, C*H*_2_CH), 3.10 (dd, ^3^*J* = 13.9 Hz, ^3^*J* = 5.7 Hz, 1H, C*H*_2_CH), 3.72 (s, 3H, OCH_3_), 4.19–4.38 (m, 2H, CCH_2_C*H*_2_), 4.49 (*pseudo*-q, ^3^*J* = 6.8 Hz, 1H, NCH), 4.98 (d, ^3^*J* = 8.3 Hz, 1H, NH), 5.22–5.45 (m, 2H, C*H*_2_OP), 6.81–6.88 (m, 1H, CH_Ar_), 6.98 (dd, ^3^*J* = 8.3 Hz, ^4^*J* = 0.9 Hz, 1H, CH_Ar_), 7.02–7.10 (m, 1H, CH_Ar_).

**^13^C NMR (125.8 MHz, chloroform-*d*):** δ_c_ 20.7 (d, ^3^*J*_C-P_ = 6.7 Hz, *C*H_2_CH_2_OP), 28.3 (C(*C*H_3_)_3_), 37.7 (*C*H_2_CHN), 52.4 (OCH_3_), 54.3 (CHN), 66.0 (d, ^2^*J*_C-P_ = 5.8 Hz, CH_2_*C*H_2_OP), 68.6 (d, ^2^*J*_C-P_ = 7.1 Hz, *C*H_2_OP), 70.6 (C*C*H), 79.0 (*C*CH), 118.8 (d, *J*_C-P_ = 8.7 Hz, CH_Ar_), 120.5 (d, ^3^*J*_C-P_ = 10.4 Hz, C_Ar_), 125.9 (d, *J*_C-P_ = 5.6 Hz, CH_Ar_), 130.6 (d, *J*_C-P_ = 5.7 Hz, CH_Ar_), 132.5 (C_Ar_), 149.2 (d, ^2^*J*_C-P_ = 7.3 Hz, POC_Ar_), 155.0 (NCO), 172.0 (CO).

**^31^P NMR (121.5 MHz, chloroform-*d*):** δ −9.9.

**HRMS (EI)^+^:**  *m*/*z* calculated for C_20_H_26_KNO_8_P [M+K]^+^ 478.1028, found 478.1039.

**(2*S*)-3-(2-(but-3-yn-1-yloxy)-2-oxido-4*H*-benzo[*d*][1,3,2]dioxaphosphinin-6-yl)-1-methoxy-1-oxopropan-2-aminium trifluoroacetate (5h) (** **Supplementary Materials Figures S13 and S14** **)**

To a solution of compound **5g** (31.7 mg, 0.072 mmol) in dry DCM (1 mL) at 0 °C, trifluoroacetic acid (1 mL) was added. The resulting reaction mixture was stirred at the same temperature for 1h. After reaction completion, volatiles were removed under vacuo to give the desired compound as a colorless oil (32.8 mg, quant. yield). Two diastereoisomers were detected by NMR spectroscopy with a diastereomeric ratio of 60:40. Unambiguous assignment of the signals to each individual diastereoisomer has not been achieved.

**^1^H NMR (500 MHz, methanol-*d*_4_):** δ = 2.329 (t, *J* = 2.70 Hz 0.6 H, CCH, major diastereoisomer), 2.331 t, *J* = 2.70 Hz 0.4H, CCH, minor diastereoisomer), 2.57–2.64 (m, 2H, CCH_2_), 3.12–3.20 (m, 1H, C*H*_2_CH), 3.22–3.29 (m, 1H, C*H*_2_CH), 3.82 (s, 3H, OCH_3_), 4.20–4.30 (m, 2H, CCH_2_C*H*_2_), 4.31–4.35 (m, 1H, NCH), 5.33–5.63 (m, 2H, C*H*_2_OP), 7.09–7.16 (m, 2H, CH_Ar_), 7.23–7.31 (m, 1H, CH_Ar_).

**^13^C NMR (125.8 MHz, methanol-*d*_4_):** δ 21.2 (d, *J* = 7.0 Hz, *C*H_2_CH_2_OP), 36.49 (*C*H_2_CHN, major diastereoisomer), 36.50 (*C*H_2_CHN, minor diastereoisomer), 53.7 (OCH_3_), 54.97 (CHN, major diastereoisomer), 54.98 (CHN, minor diastereoisomer), 67.98 (d, *J* = 5.8 Hz, CH_2_*C*H_2_OP, major diastereoisomer), 68.00 (d, *J* = 5.8 Hz, CH_2_*C*H_2_OP, minor diastereoisomer), 70.0 (d, *J* = 6.9 Hz, *C*H_2_OP), 71.7 (C*C*H), 80.12 (*C*CH, major diastereoisomer), 80.13 (*C*CH, minor diastereoisomer), 118.1 (q, *J* = 292.2 Hz, *C*F_3_COO^−^), 120.18 (d, *J* = 9.1 Hz, CH_Ar_, major diastereoisomer), 120.21 (d, *J* = 9.1 Hz, CH_Ar_, minor diastereoisomer), 122.81 (d, *J* = 9.8 Hz, C_Ar_, minor diastereoisomer), 122.85 (d, *J* = 9.8 Hz, C_Ar_, major diastereoisomer), 127.88 (d, *J* = 1.0 Hz, CH_Ar_, major diastereoisomer), 127.91 (d, *J* = 1.0 Hz, CH_Ar_, minor diastereoisomer), 132.02 (d, *J* = 1.7 Hz, CH_Ar_, major diastereoisomer), 132.06 (d, *J* = 1.7 Hz, CH_Ar_, minor diastereoisomer), 132.07 (C_Ar_, major diastereoisomer), 132.09 (C_Ar_, minor diastereoisomer), 150.9 (d, *J* = 6.7 Hz, O-C_Ar_), 162.7 (q, *J* = 35.2 Hz, CF_3_*C*OO^−^), 170.256 (CO, major diastereoisomer), 170.262 (CO, minor diastereoisomer).

**^31^P NMR (121.5 MHz, methanol-*d*_4_):** δ −9.58 (minor diastereoisomer), −9.59 (major diastereoisomer).

**^19^F NMR (282.4 MHz, methanol-*d*_4_):** δ −77.0.

**HRMS (ESI, positive mode)** calculated (*m*/*z*) for C_15_H_19_O_6_NP [M+H]^+^ 340.0945, found 340.0945.

**Methyl 4-((tert-butoxycarbonyl)amino)-2-hydroxybenzoate (17)**

Methyl 4-Amino-2-hydroxybenzoate **16** (2.5 g, 15 mmol) and Boc_2_O (3.75 mL, 16.3 mmol, 1.1 eq.) were heated at 70 °C for 3 days. The volatiles were removed under reduced pressure. Then, ethyl acetate and distilled water were added to the resulting residue, and the two layers were separated. The organic layer was washed with 2 M HCl (3 × 25 mL), brine (3 × 25 mL), dried over Na_2_SO_4_ and filtered. After removal of the volatiles under reduced pressure, the crude product was purified by flash chromatography (SiO_2_, 1:9 EtOAc/Petroleum ether) to afford an off-white solid (3.85g, 96%). The characterization data were in agreements to those previously reported in the literature [21].

**^1^H NMR (500 MHz, chloroform-*d*):** δ = 1.52 (s, 9H, C(C*H*_3_)_3_), 3.91 (s, 3H, O-C*H*_3_), 6.60 (s, 1H, N*H*), 6.93 (dd, *J* = 8.7, 2.2 Hz, 1H, C*H*_Ar_), 6.98 (d, *J* = 2.2 Hz, 1H, C*H*_Ar_), 7.73 (d, *J* = 8.7 Hz, 1H, C*H*_Ar_), 10.83 (s, 1H, O*H*).

***tert*-Butyl (3-hydroxy-4-(hydroxymethyl)phenyl)carbamate (18) (** **Supplementary Materials Figure S27** **)**

To a solution of compound **17** (2.12 g, 7.9 mmol) in distilled THF (70 mL) at −78 °C was added dropwise LiAlH_4_ (1.6 eq., 1 M solution in THF, 13 mL, 13 mmol). The reaction mixture was left overnight in the acetone/dry ice bath and allowed to warm to room temperature. The reaction mixture was quenched by dropwise addition of water. Thereafter, saturated Rochelle salt solution was added. M HCl was added dropwise until the resulting solution reached a pH of 5. The resulting mixture was extracted three times with ethyl acetate. Organic layers were combined, washed with brine, dried over Na_2_SO_4_, filtered and concentrated under reduced pressure to give the crude product as an orange residue. After purification by automated flash chromatography (from 80:20 PE/EtOAc to 50:60 PE/EtOAc), the desired product was obtained as yellow solid (1.71 g, 90%).

**^1^H NMR (500 MHz, chloroform-*d*):** δ = 1.50 (s, 9H, C(C*H*_3_)_3_), 4.72 (s, 2H, C*H*_2_), 6.53 (s, 1H, N*H*), 6.78 (dd, *J* = 8.1, 2.1 Hz, 1H, C*H_Ar_*), 6.90 (s, 1H, C*H_Ar_*), 6.93 (d, *J* = 8.2 Hz, 1H, C*H_Ar_*).

**^13^C NMR (125.8 MHz, chloroform-*d*):** δ = 28.5 (C(*C*H_3_)_3_), 63.7 (*C*H_2_), 81.0 (*C*(CH_3_)_3_), 107.0 (*C*H_Ar_), 110.4 (*C*H_Ar_), 120.5 (*C_Ar_*), 128.7 (*C*H_Ar_), 139.3 (*C_Ar_*), 153.1 (*C*O), 156.4 (*C_Ar_*).

**HRMS (ESI, positive mode)** calculated (*m*/*z*) for C_12_H_17_NNaO_2_ [M+Na]^+^ 262.1050, found 262.1049.

***tert*-Butyl (2-chloro-2-oxido-4*H*-benzo[*d*][1,3,2]dioxaphosphinin-7-yl)carbamate (19)**

The general procedure **A** was applied to synthetize compound **19** from saligenol **18** (1 eq, 990 mg, 4.2 mmol). A ^31^P-NMR confirmed the presence of the product obtained as a yellow solid (proportion: 50%).

**^31^P NMR (161.9 MHz, chloroform-*d*):** δ = −6.2.

***tert*-Butyl (2-(but-3-yn-1-yloxy)-2-oxido-4*H*-benzo[*d*][1,3,2]dioxaphosphinin-7-yl)carbamate (20) (** **Supplementary Materials Figures S28 and S29** **)**

The general procedure **B** with workup 1 was applied to synthetize compound **20** from 3-butyn-1-ol **9** (1 eq, 50 µL, 0.66 mmol). The crude was purified by automated flash chromatography (from 70:30 PE/EtOAc to 60:40 PE/EtOAc). The desired product was afforded as a light-yellow oil (59.1 mg, 25%).

**^1^H NMR (500 MHz, chloroform-*d*):** δ = 1.52 (s, 9H, C(C*H*_3_)_3_), 1.98 (t, *J* = 2.7 Hz, 1H, C*H*), 2.61 (tdt, *J* = 6.8, 2.7, 0.6 Hz, 2H, C*H*_2_CH_2_OP), 4.19–4.35 (m, 2H, CH_2_C*H*_2_OP), 5.24–5.39 (m, 2H, C*H*_2_OP), 6.56 (bs, 1H, N*H*), 6.95–6.98 (m, 1H, C*H_Ar_*), 7.10 (dd, *J* = 8.3, 2.1 Hz, 1H, C*H_Ar_*), 7.19 (d, *J* = 2.1 Hz, 1H, C*H_Ar_*).

**^13^C{^1^H} NMR (125.8 MHz, chloroform-*d*):** δ = 20.8 (d, ^2^*J*_C-P_ = 6.8 Hz, *C*H_2_CH_2_OP), 28.4 (C(*C*H_3_)_3_), 66.2 (d, *J* = 5.7 Hz, CH_2_*C*H_2_OP), 68.7 (d, *J* = 6.8 Hz, *C*H_2_OP), 70.7 (*C*H), 79.1 (*C*CH), 81.4 (*C*(CH_3_)_3_), 108.7 (d, *J* = 9.9 Hz, *C*H_Ar_), 114.2 (*C*H_Ar_), 114.8 (d, *J* = 10.0 Hz, *C*_Ar_), 125.7 (d, *J* = 1.1 Hz, *C*H_Ar_), 140.1 (d, *J* = 2.5 Hz, *C*_Ar_), 150.7 (d, *J* = 6.5 Hz, *C*_Ar_), 152.4 (*C*O).

**^31^P{^1^H} NMR (161.9 MHz, chloroform-*d*):** δ = −9.9.

**HRMS (ESI, positive mode)** calculated (*m*/*z*) for C_16_H_20_NNaO_6_P [M+Na]^+^ 376.0920, found 376.0923.

### 2.3. Biological Activity Evaluation

#### 2.3.1. Micro-Organisms and Culture Conditions

*Escherichia coli* XL1 Blue, *Klebsiella pneumoniae*, *Bacillus subtilis* and *Pseudomonas aeruginosa* were grown at 37 °C either in Luria–Bertani medium or in Mueller-Hinton medium with agitation. On Petri dishes, they were plated on Luria–Bertani medium containing 1.5% agar.

*Mycobacterium smegmatis* DSM43756 (ATCC 19420) was obtained from the Deutsche Sammlung von Microorganismen und Zellkulturen GmbH (DSMZ, Braunschweig, Germany). Bacteria were grown aerobically at 37 °C in a liquid medium containing 0.4% yeast extract, 1% malt extract, 0.2% CaCO_3_ and 0.4% D-glucose. They were plated on Petri dishes containing the same medium with 1.5% agar.

#### 2.3.2. Antimicrobial Activity of Prodrugs

The antimicrobial activity against *M. smegmatis*, *E. coli*, *K. pneumoniae*, *B. subtilis* and *P. aeruginosa* was determined using the paper disc diffusion method in Petri plates or liquid medium in 96-well microplates.

*Paper disc diffusion method:*

One or two bacterial colonies picked from a freshly streaked Petri plate (24 h) are inoculated in LB broth and grown until the exponential phase. The culture was then five-fold diluted and 150 µL of the suspension were used to inoculate the Petri plates with pre-sterilized glass beads (3–4 mm). The discs are then deposited with forceps on the surface of the appropriate medium and impregnated with the compounds to be tested and control molecules (fosmidomycin or isoniazid).

Prodrugs, not soluble in water, were dissolved in DMSO (4 mM and 100 mM). Fosmidomycin and isoniazid were dissolved in water (1 mM and 5 mM,, respectively) and were used as controls for *E. coli* and *M. smegmatis* respectively. DMSO was used as negative control. Paper discs (diameter 6 mm) were impregnated with the prodrug solutions (8 µL), fosmidomycin (2 µL) or isoniazid (6 µL) and placed on Petri dishes. Bacterial growth inhibition was examined after a 24 h incubation at 37 °C for *E. coli*, and after a 48 h-incubation at 30 °C for the mycobacteria. The diameter (in mm) of the circular zone of inhibition around each disc was measured. All tests were performed in three independent replicates.

*Antimicrobial activity of prodrugs in liquid medium:*

A direct Mueller–Hinton broth suspension (4–5 mL) of isolated bacterial colonies selected from an 18- to 24 h LB agar plate was prepared. The broth culture was incubated overnight at 37 °C. The cell density of the culture was then adjusted to approximately 5·10^5^ cells/mL in the same medium.

In a 96-well sterile microplate, the compounds to be tested (in DMSO) and the bacterial suspension in Mueller–Hinton broth (5·10^5^ cells/mL) were dispensed in a final volume of 200 µL. Bacterial suspension was added within 15 min after being adjusted at 5·10^5^ cells/mL. Final concentrations in the microwells of antimicrobial compounds were 100 µM or 2.5 mM, and volumes did not exceed 3.75 µL/microwell. Positive growth control microwell (bacteria in MH broth), negative growth control (bacteria in MH broth with fosmidomycin), growth control microwell (bacteria in MH broth with DMSO) and sterility control microwell (only MH broth) were included in each test. The microplates were parafilmed and incubated at 37 °C for 24 h. Bacterial growth or absence of bacterial growth was detected by the naked eye. All tests were performed in three independent replicates.

MIC determinations against *M. smegmatis* were performed using the same protocol in sterile 96-well microplates, with Middlebrook 7H9 as the culture medium. From a stock solution of each compound in DMSO, two-fold serial dilutions were prepared. The lowest concentration that completely inhibits bacterial growth was detected by the naked eye. All tests were performed in three independent replicates.

## 3. Results and Discussion

### 3.1. Chemistry

**Scheme 5:**
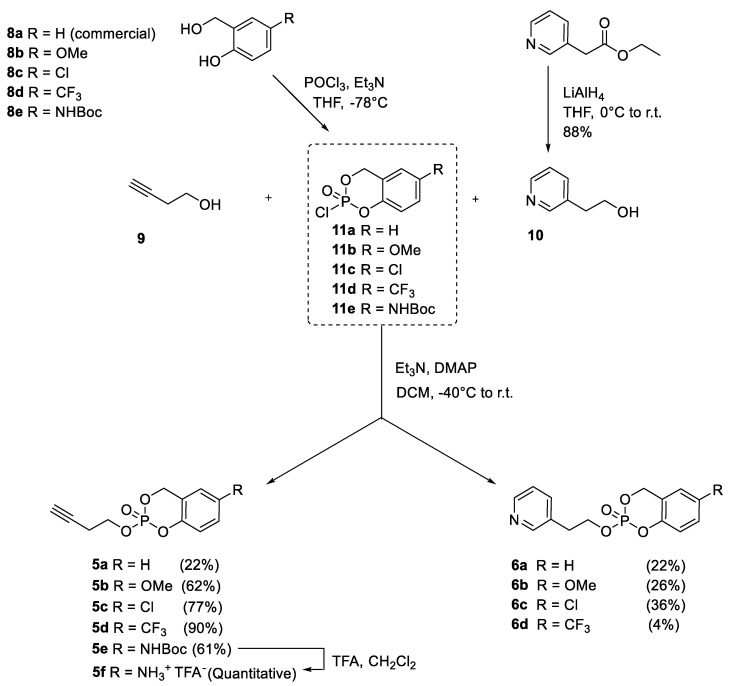
Synthesis of the monophosphate prodrugs **5a**–**f** and **6a**–**d**.

**Scheme 6:**
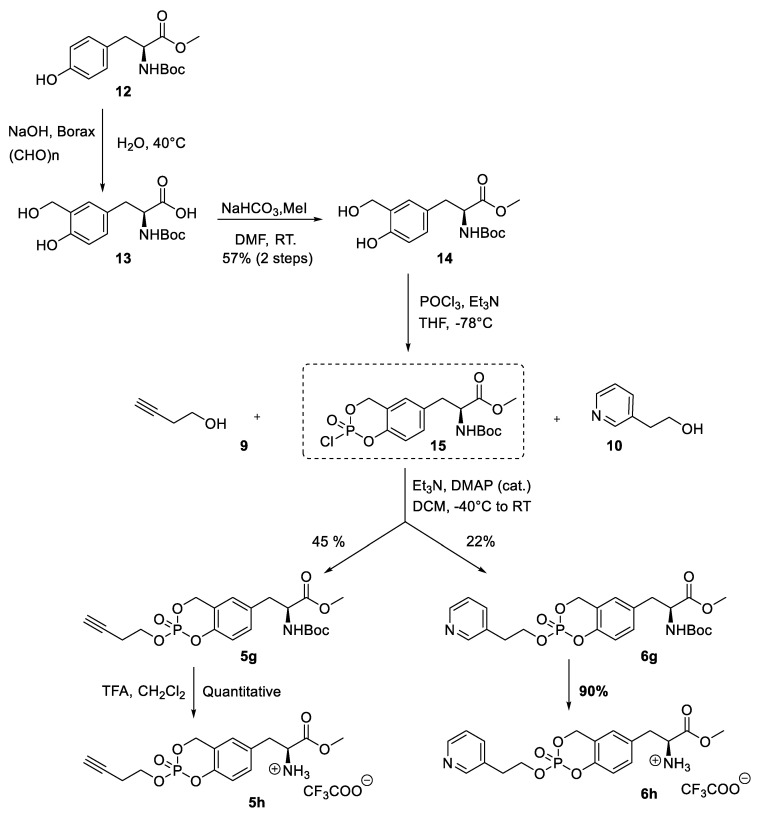
Synthesis of the monophosphate prodrugs **5h** and **6h**.

**Scheme 7:**
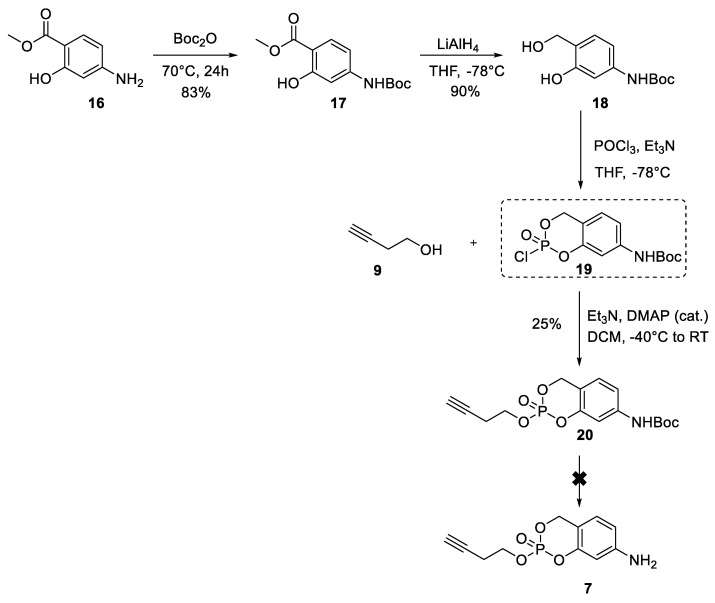
Synthesis of the monophosphate prodrugs **7**.

The key step to synthesize the *cyclo*Sal prodrugs **5**–**7** is the addition–elimination of the commercially available butyn-1-ol **9** and the 2-(pyridin-3-yl)ethan-1-ol **10**, obtained by reduction of the commercial ethyl 2-(pyridin-3-yl)acetate in 88% yield, on the *cyclo*Sal phosphochloridate derivatives **11** (Scheme 5), **15** (Scheme 6) and **19** (Scheme 7) obtained by phosphorylation of the differently substituted salicyl alcohols using phosphorus (V) chemistry as previously described [7].

Thus, a series of substituted salicyl alcohols was first required to synthesize the substituted *cyclo*Sal prodrugs **5a**–**f** and **6a**–**d**. Since salicylic alcohol **8a** is the only commercially available compound, the others **8b**–**d** were obtained by reducing their corresponding carboxylic acids, as previously described [7]. Compound **8e** was synthesized in three steps from methyl 2-hydroxy-5-nitrobenzoate by reduction of the nitro group, followed by Boc protection of the resulting amino group [19], and finally by reduction of the methyl ester to the corresponding benzyl alcohol with LiAlH_4_. Thus, the alkyne monophosphate inhibitor prodrugs **5a**–**f** could be isolated in yields ranging from 22 to 90% (Scheme 5). Concerning the pyridine series, the prodrugs **6a**–**d** were obtained in yields of between 4 and 36% for the coupling step. These lower yields compared to the alkyne inhibitor could be explained by the treatment of the crude mixture. As the pH of the reaction medium is very acidic (around 1–2), the prodrugs **6a**–**d** are in pyridinium form, and most of them pass into the aqueous layer while washing the organic one. The aqueous layer must then be sufficiently basified (at least pH 6) to extract the product but not exceed a pH of 7 to avoid deprotection of the prodrug. Even so, some prodrugs may have remained in the aqueous layer or undergone deprotection during the process. In particular, the poor yield of compound **6d** could be due to its substitution with the strongly electron-withdrawing group CF_3_ that could induce a very short half-life for this prodrug.

In parallel, we have focused on the mutual prodrug strategy and the synthesis of the amino acid-functionalized *cyclo*Sal prodrugs **5g** and **6g** where the carboxylic acid was protected by a methyl ester and the amine by a Boc moiety to ensure that these prodrugs enter the cell. Then the Boc deprotection could lead to desired prodrugs **5h** and **6h**.

The synthesis of *cyclo*Sal phosphochloridate **15** begins with the regioselective introduction of a benzyl alcohol group at the *ortho* position of commercially available protected tyrosine **12** (Scheme 6). This transformation was achieved via an electrophilic aromatic substitution using formaldehyde in aqueous medium, in the presence of sodium hydroxide and sodium tetraborate decahydrate (borax), following the method reported by Huang [21]. The crude reaction mixture was analyzed by ^1^H NMR, showing a characteristic benzylic CH_2_ signal at 4.44 ppm, confirming the successful incorporation of the benzyl alcohol moiety. However, the expected singlet corresponding to the methyl ester group was no longer detected, indicating hydrolysis under the basic aqueous conditions. To restore the ester functionality, the crude product was subjected to a selective methylation step using iodomethane and sodium hydrogen carbonate in DMF. This two-step sequence afforded the salicyl alcohol derivative **14** in 57% overall yield. Its structure was confirmed by NMR spectroscopy, with a singlet at 3.71 ppm in the ^1^H NMR spectrum and a signal at 52.3 ppm in the ^13^C NMR spectrum, corresponding to the methyl ester group. Subsequent phosphorylation of saligenol **14** using POCl_3_ afforded the *cyclo*Saligenyl chlorophosphate **15**, as indicated by a major signal at −6.0 ppm in the crude ^31^P NMR spectrum. The latter was then directly involved in the key coupling step with either butyn-1-ol **9** or 2-(pyridin-3-yl)ethan-1-ol **10**.

In the case of the alkyne inhibitor, the coupling reaction between commercially available 3-butyn-1-ol **9** and *cyclo*Sal chlorophosphate **15** afforded the desired prodrug **5g**, which was isolated in 45% yield (Scheme 6). The Boc deprotection using TFA provided the expected prodrug **5h** as its ammonium triflate salt in quantitative yield.

Concerning the pyridine-based inhibitor, the coupling reaction between the previously synthesized 2-(pyridin-3-yl)ethan-1-ol **10** and *cyclo*Sal chlorophosphate **15** led to **6g** with 22% yield. After deprotection of the carbamate protecting group with TFA, **6h** was obtained as a triflate ammonium salt with 90% yield.

The synthesis of the mutual prodrug **7** started with the protection of the amino group of the commercially available methyl 4-amino-2-hydroxybenzoate **16** as previously described in the literature [22], followed by the reduction of the methyl ester to produce the *cyclo*Saligenol derivative **18** in 90% yield in two steps. Subsequent conversion to corresponding *cyclo*Saligenyl chlorophosphite **19** and condensation with alkyne **9** under previously described conditions led to the prodrug **20** in 25%. Attempted deprotection of the Boc group under acidic conditions (using TFA or HCl) resulted in the degradation of the compound, which prevented isolation of the desired free amine prodrug **7** (Scheme 7).

### 3.2. Biological Evaluation

The ability of *cyclo*Sal prodrugs to inhibit the growth of *E. coli*, *K. pneumoniae*, *B. subtilis*, *P. aeruginosa* and *M. smegmatis* was assessed by two complementary methods: agar diffusion in Petri plates (Kirby–Bauer disk diffusion method) and microdilution in microwell plates for minimum inhibitory concentrations (MICs) determination.

#### 3.2.1. I-Bacterial Growth Inhibition Assessment Using the Agar Diffusion Method

We tested the *cyclo*Sal prodrugs by agar diffusion method on *E. coli*, *K. pneumoniae*, *B. subtilis* and *P. aeruginosa* bacterial lawn loading 800 nmol of product on the cellulose discs (6 mm). No growth inhibition was observed. These results are consistent with observations made when testing *cyclo*Sal prodrugs of DXR inhibitors, which were also unable to inhibit *E. coli* growth compared to the reference fosmydomycin.

**Figure 1:**
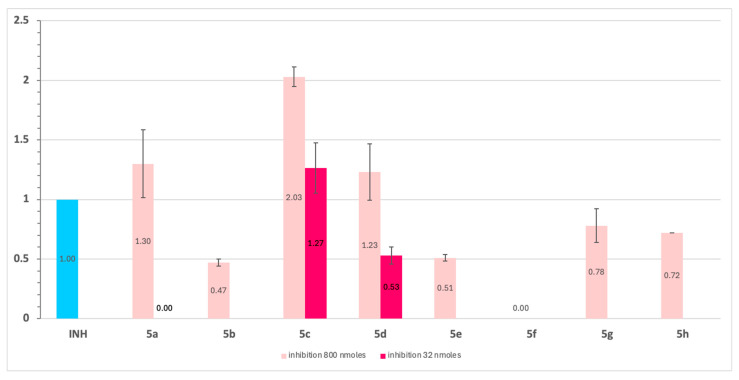
*M. smegmatis* growth inhibition of compounds **5a**–**h** compared to reference isoniazid (INH). The inhibitory activity of the compounds was quantified by expressing their inhibition zone diameters as ratios relative to that of INH, which was set as the reference value of 1 (shown in cyan). In light pink: ratios of compounds at 800 nmol. In deep pink: ratios of compounds at 32 nmol. **5a** and **5f** values (32 nmol) have been tested and equal 0. Data represent mean ± standard error (SE) from three independent biological replicates.

**Figure 2:**
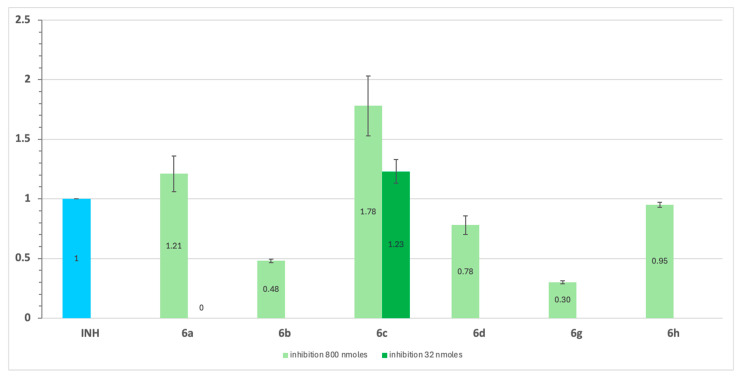
*M. smegmatis* growth inhibition of compounds **6a**–**d** and **6g**–**h** compared to reference INH. The inhibitory activity of the compounds was quantified by expressing their inhibition zone diameters as ratios relative to that of INH, which was set as the reference value of 1 (shown in cyan). In light green: ratios of compounds at 800 nmol. In deep green: ratios of compounds at 32 nmol. **6a** value (32 nmol) has been tested and equal 0. Data represent mean ± standard error (SE) from three independent biological replicates.

We then evaluated the *cyclo*Sal prodrugs (800 and 32 nmol) under the same experimental conditions on a *M. smegmatis* bacterial lawn and compared their inhibitory activity to that of 30 nmol of isoniazid (Figure 1 and Figure 2).

When 800 nmol of product were loaded on the cellulose discs, *cyclo*Sal prodrugs **5c** and **6c** exhibited significantly stronger growth inhibition than INH with ratios of 1.98 and 1.73, respectively, while compound **5d** showed a growth inhibitory activity slightly higher to that of INH (ratio of 1.18). Notably, all three *cyclo*Sal moieties in these compounds, **5c**,**d** and **6c**, were substituted by an electron-withdrawing group (Cl or CF_3_). These results suggest that the prodrug enters the cell via passive diffusion and releases the monophosphate, which is then phosphorylated to form the active IspH diphosphate inhibitors **2** and **3** responsible for the observed growth inhibition. In fact, within the active site, the diphosphate is inserted into a polar pocket remote from the [3Fe-4S] cluster, in which an inorganic diphosphate was identified during the first crystallizations of the enzyme [23]. Therefore, although no monophosphate inhibitors have been experimentally evaluated against IspH, it can be inferred that such compounds possess a reduced affinity for the enzyme’s active site and exhibit minimal or no inhibitory activity. These findings support the hypothesis that phosphorylation occurs in vivo, yielding the corresponding biologically active diphosphate derivatives. Enzymatic inhibition tests complemented by crystallographic studies with monophosphate analogs of IspH inhibitors would ensure that the inhibitory activity is entirely due to diphosphates after an in vivo phosphorylation step. However, it cannot be excluded that monophosphate compounds may inhibit another metabolic pathway essential to the bacteria.

Moreover, the best *M. smegmatis* growth inhibition was observed with *cyclo*Sal **5c** and **6c** substituted by a Cl atom, whereas unsubstituted *cyclo*Sal **5a**, **6a** were the least effective. Nevertheless, compounds **5a** and **6a** exhibited inhibitory activity against *M. smegmatis* that was slightly higher than that of the reference compound, isoniazid, with ratio of 1.30 and 1.21 (Figure 1 and Figure 2), respectively, thereby raising questions about the possible role of saligenol. Although its potential contribution cannot be entirely excluded, it is considered unlikely. Indeed, once inside the cell, saligenol would be rapidly oxidized into salicylic acid, which in turn would be immediately utilized as a precursor in the biosynthesis of mycobactins, a class of siderophore molecule which contain a salicylic acid-derived moiety [24]. It is therefore reasonable to assume that the observed inhibitory activity does not result from saligenol itself, but rather from the release of the inhibitory moiety of the compound. In contrast, prodrugs **5b** (ratio 0.47) and **6b** (ratio 0.48) bearing a methoxy electron-donating group exhibited markedly reduced inhibitory effects compared to INH (Figure 1 and Figure 2). The lack of activity may be attributed to their slower hydrolysis kinetics, resulting in lower intracellular concentration of inhibitor. Indeed, if the release of the active molecule is too slow, the prodrug could (i) exit the bacterial cell, either by passive diffusion or through efflux pumps, or (ii) be degraded by enzymes such as phosphatases.

These results suggest that the prodrugs’ efficacy correlates with the release kinetics of the active compound. Indeed, the deprotection kinetics of antivirals *cyclo*Sal prodrugs strongly depend on the para-substituents of the phenol ester: electron-withdrawing groups (Cl or NO_2_) accelerate hydrolysis, whereas electron-donating groups will have the opposite effect. In addition, the slightly higher electronegativity of the CF_3_ group compared to chlorine [25] suggests that a CF_3_-substituted *cyclo*Sal prodrug hydrolyzes more rapidly than a Cl-substituted prodrug, resulting in the inhibitor being released before entering the bacterium. The lower efficacy observed for compounds **5d**, **6d** compared with **5c**, **6c** is indeed in line with this hypothesis. The earlier explanation of saligenol potential inhibitory activity can likewise be used to exclude inhibition by chlorosaligenol and trifluoromethylsaligenol for these compounds.

The alkyne prodrug **5e**, in which the amine is protected with a Boc group, exhibited approximately half the inhibitory activity of isoniazid against *M. smegmatis*. This lower activity is consistent with the reduced electron-donating character and increased steric demand of the Boc-protected amine, which are expected to attenuate prodrug hydrolysis. The deprotected prodrug **5f**, isolated as its triflate salt, showed no growth inhibition, as anticipated, the ammonium salt hindering its diffusion across cellular membrane. Interestingly, compound **20** (0.52 ± 0.03) exhibited approximately the same ratio as prodrug **5e** (0.51), confirming that the position of the amine substituent is not a critical factor.

The alkyne and pyridine prodrugs functionalized with an amino acid residue, either with a protected (**5g**, ratio 0.78, **6g** ratio 0.30) or with a free amine group (**5h**, ratio 0.72, **6h** 0.95), did not exhibit significant inhibitory activity, remaining below that of the reference compound, isoniazid. Both alkyne and pyridine prodrugs of the monophosphate analog of the IspH inhibitor exhibited lower inhibitory activity than the non-substituted prodrug **5a** and the chlorine- or trifluoromethyl-substituted prodrugs **5c** and **5d**, respectively, which is probably due to the donor character of the amino acid alkyl chain [26]. In these molecules, the amine function is located further from the aromatic ring, and thus electronic effects no longer influence the kinetics of inhibitor release inside the cell. These results are interesting and open the way to the synthesis of mutual prodrugs in which the amino acid residue could be used. In addition, we observed a slightly better efficacy of the alkyne prodrug series **5** compared to the pyridine prodrugs **6**, consistent with previous results that demonstrated the higher affinity of the alkyne diphosphate inhibitor for the IspH (LytB) enzyme of *Aquifex aeolicus* (IC_50_ = 450 nM vs. 9.1 mM) [11]. This difference may also be partially explained by the pyridine motif: its nitrogen atom could be protonated in the extracellular medium, potentially reducing membrane permeability and thereby limiting bacterial uptake of the corresponding prodrugs.

When *M. smegmatis* growth inhibition observed at 800 nmol was equal to or greater than that of the reference dose of INH, the corresponding prodrugs were subsequently tested at 32 nmol. Specifically, *cyclo*Sal prodrugs **5a**,**c**,**d** and **6a**,**c** which efficiently inhibited *M. smegmatis* growth—showing greater activity than the INH reference—were included in this subsequent analysis (Figure 1 and Figure 2). At this dose, only the *cyclo*Sal prodrugs **5c** (ratio 1.27) and **6c** (ratio 1.23) exhibited a growth inhibition higher than the INH. Prodrug **5d** (ratio 0.53) inhibited *M. smegmatis* growth but was less effective than INH.

Therefore, based on these findings, *cyclo*Sal prodrugs **5c** and **6c** appear as promising candidates for the development of novel antimycobacterial agents.

#### 3.2.2. II-Bacterial Growth Inhibition Assays in Liquid Media

Liquid-based assays offer a complementary approach to traditional plate-based methods for evaluating inhibitory activity. These tests eliminate the variables of agar matrix and diffusion rate and expose planktonic cells to a uniform concentration of the drug. Therefore, inhibitor concentrations are usually lower than in Petri plates tests.

Thus, we also evaluated the inhibitory activity of *cyclo*Sal prodrugs in liquid culture medium in 96-well microplates against *E. coli*, *K. pneumoniae*, *B. subtilis* and *P. aeruginosa*. Prodrugs concentrations of 100 µM or 2.5 mM were tested. Fosmidomycin was used as the reference antibiotic in these tests at a concentration of 25 µM. All the results are reported in Table 1.

At a concentration of 100 µM, none of the prodrugs exhibited antibacterial activity. However, at 2.5 mM, the unsubstituted prodrug **5a** inhibited the growth of *E. coli*, *K. pneumoniae* and *B. subtilis*, as evidence by the absence of turbidity visible to the naked eye after 24 h of incubation. Furthermore, in the presence of prodrugs **5b**–**d**, **6a** and **6b**,**c**, a cloudiness turbidity of variable intensity was observed, but at a lower cell density than in the growth control where the three bacterial strains were grown without antibiotics. These results indicate that, although turbidity is present, prodrugs **5b**–**d**, **6a** and **6b**,**c** induce a slowdown in bacterial growth compared to the untreated control. Indeed, as the prodrugs are in solution in the extracellular medium, *cyclo*Sal substituted by an electron-withdrawing group **5c**,**d** and **6c** may undergo hydrolysis before entering the cell, whereas in the presence of the methoxy group (**5b** and **6b**), release of the active compound could be too slow, preventing intracellular accumulation of the inhibitor. Furthermore, the difference in activity observed between the unsubstituted prodrugs **5a** and **6a** of the alkyne and pyridine series, respectively, could be explained by the greater affinity of the alkyne diphosphate inhibitor for the enzyme, and to the potential protonation of the pyridine nitrogen in the medium, which could reduce its bioavailability. Regarding *P. aeruginosa*, none of the prodrugs tested showed inhibitory activity against this bacterium.

Since compound **5a** exhibited an inhibitory activity at 2.5 mM but not at 100 µM, we determined its MIC on *E. coli*, *K. pneumoniae* and *B. subtilis*. The growth inhibition test is performed in a 96-well microplate using five concentrations of **5a** ranging from 2.5 mM to 156 µM with a two-fold serial dilution. Total growth inhibition was observed for all three bacteria at the highest concentration (2.5 mM), confirming previous results. However, at concentrations between 1.25 and 0.312 mM, a slowdown in growth was observed as the cell density remained lower than that of the untreated growth control. No growth inhibition was observed at the lowest tested concentration of 156 µM. Thus, prodrug **5a** is only a weak inhibitor, with a MIC of 2.5 mM on the three bacterial strains tested. In order to be able to better evaluate and possibly quantify the observed growth slowdowns in *E. coli*, *K. pneumoniae* and *B. subtilis*, we plan to carry out kinetic monitoring of bacterial growth by measuring optical density over time.

Growth inhibition assays on *M. smegmatis* were also performed in liquid media, but only for prodrugs that were effective at 32 nmol on Petri plates. Accordingly, *cyclo*Sal prodrugs **5a**–**d** and **6a**–**c** were tested over a concentration range of 2.5 mM to 30 µM to determine their MICs (Table 2).

INH was used as the reference antibiotic in these tests at a concentration of 150 µM. Only compounds **5d**, **5c** and **6c** inhibited *M. smegmatis* growth with MIC values of 312 µM, 156µM and 62.5 µM, respectively. This result is consistent with the inhibitory activities observed in the previous agar diffusion assay where these two chlorinated and the fluorinated prodrugs showed the highest activity. Moreover, as previously mentioned, the trifluoromethyl group which is slightly more electron-withdrawing than chlorine may lead to a more rapid hydrolysis of the prodrug before cellular uptake. This could result in lower intracellular concentrations and consequently weaker inhibitory activity. Interestingly, compound **6c** demonstrated a slightly greater activity compared to compound **5c** in these tests. Indeed, the pyridine prodrug **6c** might enter the bacterium more efficiently than the alkyne prodrugs **5c**,**d** due to its better aqueous solubility. This difference in solubility may influence the effective concentration of each compound in aqueous medium conditions, potentially accounting for the reduced inhibitory activity observed with the alkyne-containing derivative.

## 4. Conclusions

To enhance cellular uptake of the IspH inhibitors monophosphate analogs, we implemented a *cyclo*Sal prodrug strategy consisting of masking the phosphate groups of these inhibitors with *cyclo*Sal prodrugs. The fourteen prodrugs **5a**–**h** and **6a**–**d**,**g**,**h,** corresponding to alkyne and pyridine monophosphate inhibitors, respectively, were synthesized and evaluated for antibacterial activity on a panel of model bacteria. Two prodrugs inhibited *M. smegmatis* growth in the agar diffusion assay, with the chlorine-substituted *cyclo*Sal prodrugs **5c** and **6c** demonstrating the strongest activity. Their enhanced activity likely results from faster in vivo hydrolysis allowing intracellular release of the monophosphate, which is then phosphorylated to the active diphosphate inhibitor. In liquid media, only *cyclo*Sal prodrugs with electron-withdrawing substituents **5d**, **5c** and **6c** showed significant *M. smegmatis* growth inhibition, 312 µM, 156 µM and 62.5 µM, respectively, consistent with their strong activity in agar diffusion assays. Moreover, a “mutual” prodrug approach was implemented leading to the *N*-protected derivative of **7**. These findings highlight the *cyclo*Sal prodrug strategy as a promising approach for the in vivo delivery of antibacterial agents. Synthetic efforts are currently underway to expand this strategy to the known IspH inhibitors.

## Figures and Tables

**Table 1:** Antibacterial activity of prodrugs (2.5 mM and 100 µM) against bacterial strains in microplates. Positive control (T+, culture medium), negative control (Fos, fosmidomycin).

	T+	Fos	5a		5b		5c		5d		6a		6b		6c	
		25 µM	2.5 mM	100 µM	2.5 mM	100 µM	2.5 mM	100 µM	2.5 mM	100 µM	2.5 mM	100 µM	2.5 mM	100 µM	2.5 mM	100 µM
*E. coli*	+	−	−	+	(−)	+	(−)	+	(−)	+	(−)	+	(−)	+	(−)	+
*B. subtilis*	+	−	−	+	(−)	+	(−)	+	(−)	+	(−)	+	(−)	+	(−)	+
*K. pneumoniae*	+	−	−	+	(−)	+	(−)	+	(−)	+	(−)	+	(−)	+	(−)	+
*P. aeruginosa*	+	−	+	+	+	+	+	+	+	+	+	+	+	+	+	+

**Table 2:** Minimum Inhibitory Concentrations (MICs) of prodrugs against *M. smegmatis* in microplates.

Prodrug	Tested Range (µM)	*M. smegmatis* MIC
**5a**	(30–2500)	>2500
**5b**	(30–2500)	>2500
**5c**	(30–2500)	156
**5d**	(30–2500)	312
**6a**	(30–2500)	>2500
**6b**	(30–2500)	>2500
**6c**	(30–2500)	62.5

## Data Availability

The original contributions presented in this study are included in the article. Further inquiries can be directed to the corresponding author.

## Supplementary Materials

The following supporting information can be downloaded at https://www.mdpi.com/article/10.3390/microorganisms14010215/s1, Figures S1–S29: NMR spectra of **5a**–**h**, **6a**–**d**,**g**,**h**, **18** and **20**.

## References

[1] Murray C.J.L., Ikuta K.S., Sharara F., Swetschinski L., Robles Aguilar G., Gray A., Han C., Bisignano C., Rao P., Wool E. (2022). Global Burden of Bacterial Antimicrobial Resistance in 2019: A Systematic Analysis. Lancet.

[2] Kavanagh K. (2025). The Rise of ‘Nightmare Bacteria’: Antimicrobial Resistance in Five Charts. Nature.

[3] WHO (2024). Global Tuberculosis Report 2024.

[4] WHO Tuberculosis, Key Facts 2025. https://www.who.int/teams/global-programme-on-tuberculosis-and-lung-health/tb-reports/global-tuberculosis-report-2025.

[5] Heuston S., Begley M., Gahan C.G.M., Hill C. (2012). Isoprenoid Biosynthesis in Bacterial Pathogens. Microbiology.

[6] Allamand A., Piechowiak T., Lièvremont D., Rohmer M., Grosdemange-Billiard C. (2023). The Multifaceted MEP Pathway: Towards New Therapeutic Perspectives. Molecules.

[7] Munier M., Tritsch D., Lièvremont D., Rohmer M., Grosdemange-Billiard C. (2023). New Application of cycloSaligenyl Prodrugs Approach for the Delivery of Fosfoxacin Derivatives in Mycobacteria. Molecules.

[8] Rao G., Oldfield E. (2016). Structure and Function of Four Classes of the 4Fe–4S Protein, IspH. Biochemistry.

[9] Janthawornpong K., Krasutsky S., Chaignon P., Rohmer M., Poulter C.D., Seemann M. (2013). Inhibition of IspH, a [4Fe–4S]^2+^ Enzyme Involved in the Biosynthesis of Isoprenoids via the Methylerythritol Phosphate Pathway. J. Am. Chem. Soc..

[10] Guerra F., Wang K., Li J., Wang W., Liu Y.-L., Amin S., Oldfield E. (2014). Inhibition of the 4Fe–4S Proteins IspG and IspH: An EPR, ENDOR and HYSCORE Investigation. Chem. Sci..

[11] Wang K., Wang W., No J.-H., Zhang Y., Zhang Y., Oldfield E. (2010). Inhibition of the Fe_4_ S_4_ -Cluster-Containing Protein IspH (LytB): Electron Paramagnetic Resonance, Metallacycles, and Mechanisms. J. Am. Chem. Soc..

[12] Pradere U., Garnier-Amblard E.C., Coats S.J., Amblard F., Schinazi R.F. (2014). Synthesis of Nucleoside Phosphate and Phosphonate Prodrugs. Chem. Rev..

[13] Peyrottes S. (2004). SATE Pronucleotide Approaches: An Overview. Mini Rev. Med. Chem..

[14] Cahard D. (2004). Aryloxy Phosphoramidate Triesters as Pro-Tides. Mini Rev. Med. Chem..

[15] Drontle D. (2004). Designing a Pronucleotide Stratagem: Lessons from Amino Acid Phosphoramidates of Anticancer and Antiviral Pyrimidines. MiniRev. Med. Chem..

[16] Munier M., Tritsch D., Lièvremont D., Rohmer M., Grosdemange-Billiard C. (2019). Synthesis and Biological Evaluation of Aryl Phosphoramidate Prodrugs of Fosfoxacin and Its Derivatives. Bioorg. Chem..

[17] Donald P.R., Diacon A.H. (2015). Para-Aminosalicylic Acid: The Return of an Old Friend. Lancet Infect. Dis..

[18] Chakraborty S., Gruber T., Barry C.E., Boshoff H.I., Rhee K.Y. (2013). *Para.*-Aminosalicylic Acid Acts as an Alternative Substrate of Folate Metabolism in *Mycobacterium tuberculosis*. Science.

[19] Ziani-Cherif H., Imachi K., Matsuda T. (1999). Preparation of Aryldiazonium-, Aryldiazo-, and Arylazido-Derivatized Copolymers and Their Surface Photografting. Macromolecules.

[20] Schmidt B., Hölter F., Berger R., Jessel S. (2010). Mizoroki-Heck Reactions with 4-Phenoldiazonium Salts. Adv. Synth. Catal..

[21] Huang M., Song L., Liu B. (2010). Construction of the Cyclophane Core of the Hirsutellones via a RCM Strategy. Org. Lett..

[22] Durcik M., Toplak Ž., Zidar N., Ilaš J., Zega A., Kikelj D., Mašič L.P., Tomašič T. (2020). Efficient Synthesis of Hydroxy-Substituted 2-Aminobenzo[d]Thiazole-6-Carboxylic Acid Derivatives as New Building Blocks in Drug Discovery. ACS Omega.

[23] Gräwert T., Rohdich F., Span I., Bacher A., Eisenreich W., Eppinger J., Groll M. (2009). Structure of Active IspH Enzyme from *Escherichia coli* Provides Mechanistic Insights into Substrate Reduction. Angew. Chem. Int. Ed..

[24] De Voss J.J., Rutter K., Schroeder B.G., Su H., Zhu Y., Barry C.E. (2000). The Salicylate-Derived Mycobactin Siderophores of *Mycobacterium tuberculosis* Are Essential for Growth in Macrophages. Proc. Natl. Acad. Sci. USA.

[25] Meier C., Lorey M., De Clercq E., Balzarini J. (1997). Cyclic Saligenyl Phosphotriesters of 2′,3′-Dideoxy-2′,3′-Didehydrothymidine (d4T)—A New pro-Nucleotide Approach. Bioorg. Med. Chem. Lett..

[26] True J.E., Thomas T.D., Winter R.W., Gard G.L. (2003). Electronegativities from Core-Ionization Energies:  Electronegativities of SF_5_ and CF_3_. Inorg. Chem..

