# Supplementary material for: MEP Pathway: First-Synthesized IspH-Directed Prodrugs with Potent Antimycobacterial Activity
Source: Microorganisms. 2026 Jan 17;14(1):215. doi: 10.3390/microorganisms14010215 (PMC12844193; doi:10.3390/microorganisms14010215)

MEP Pathway: First-Synthesized IspH-Directed Prodrugs with Potent Antimycobacterial Activity

Alizée Allamand, Ludovik Noël-Duchesneau, Cédric Ettelbruck, Edgar De Luna, Didier Lièvremont and Catherine Grosdemange Billiard*

Laboratoire Chimie et Biochimie de Molécules Bioactives – Université de Strasbourg/CNRS, UMR 7177, Institut Le Bel, 4 rue Blaise Pascal, 67081 Strasbourg, France.

Table of contents

^{1}H, ^{13}C, ^{31}P spectra of **5a**	S2-S3
^{1}H, ^{13}C, ^{31}P spectra of **5b**	S3-S4
^{1}H, ^{13}C, ^{31}P spectra of **5c**	S5-S6
^{1}H, ^{13}C, ^{31}P, ^{19}F spectra of **5d**	S6-S8
^{1}H, ^{13}C, ^{31}P spectra of **5e**	S8-S9
^{1}H, ^{13}C, ^{31}P,^{19}F spectra of **5f**	S10-S11
^{1}H, ^{13}C, ^{31}P spectra of **5g**	S12-S13
^{1}H, ^{13}C, ^{31}P,^{19}F spectra of **5h**	S13-S14
^{1}H, ^{13}C, ^{31}P spectra of **6a**	S16-S17
^{1}H, ^{13}C, ^{31}P spectra of **6b**	S17-S18
^{1}H, ^{13}C, ^{31}P spectra of **6c**	S19-S20
^{1}H, ^{13}C, ^{31}P, ^{19}F spectra of **6d**	S20-S22
^{1}H, ^{13}C, ^{31}P spectra of **6g**	S22-S24
^{1}H, ^{13}C, ^{31}P spectra of **6h**	S24-S26
^{1}H, ^{13}C spectra of **18**	S27
^{1}H, ^{13}C, ^{31}P spectra of **20**	S28-S29

Compound 5a

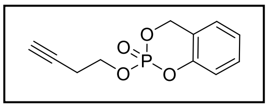

^{1}H NMR spectrum of ***5a*** *(500 MHz, chloroform-d)*

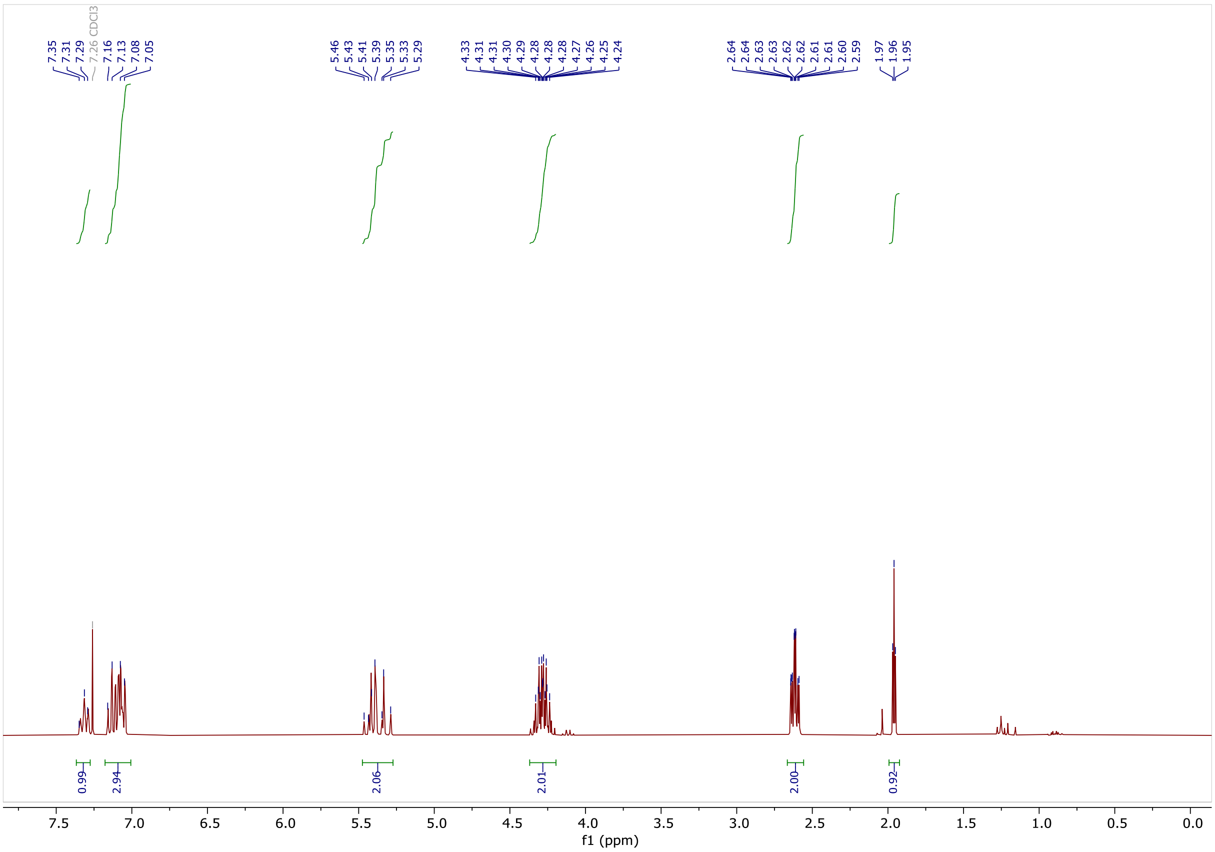

^{13}C NMR spectrum of ***5a*** *(125.8 MHz, chloroform-d)*

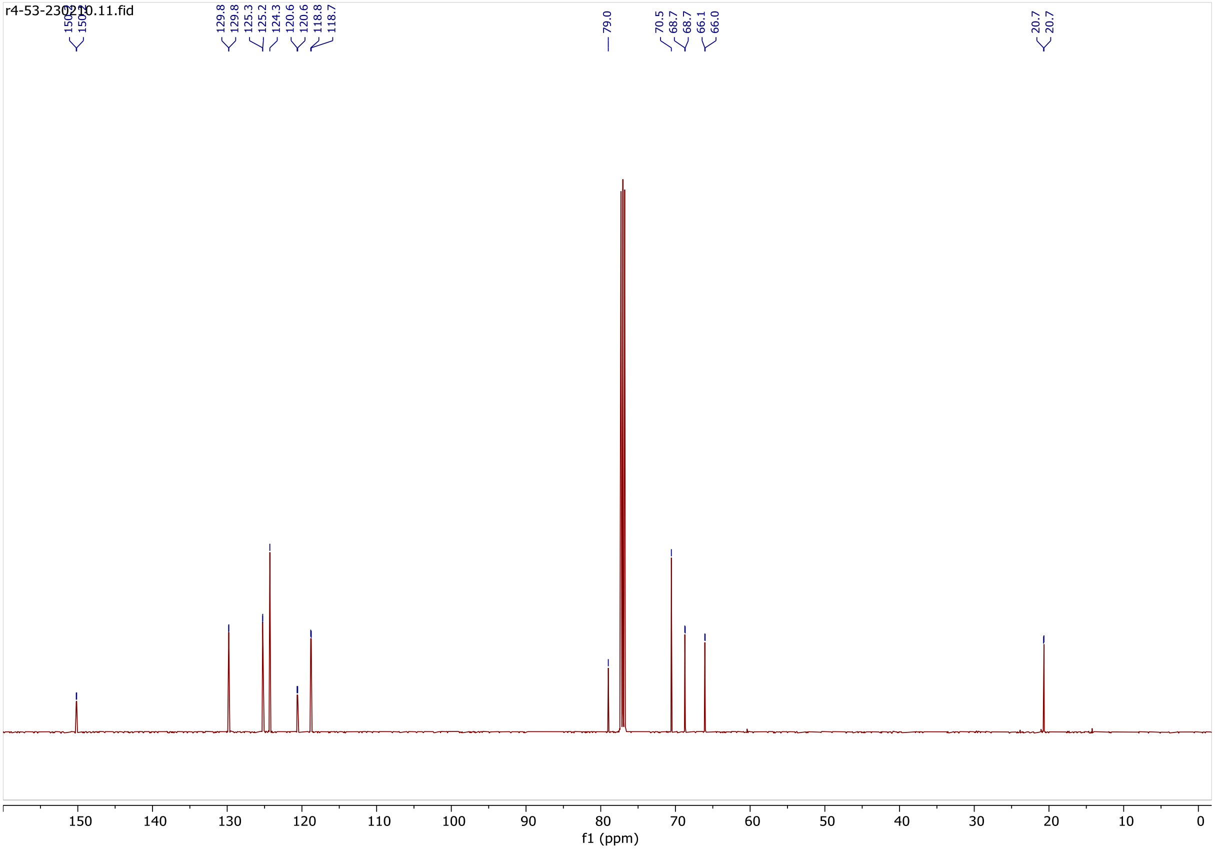

^{31}P NMR spectrum of **5a** (121.5 MHz, chloroform-d)

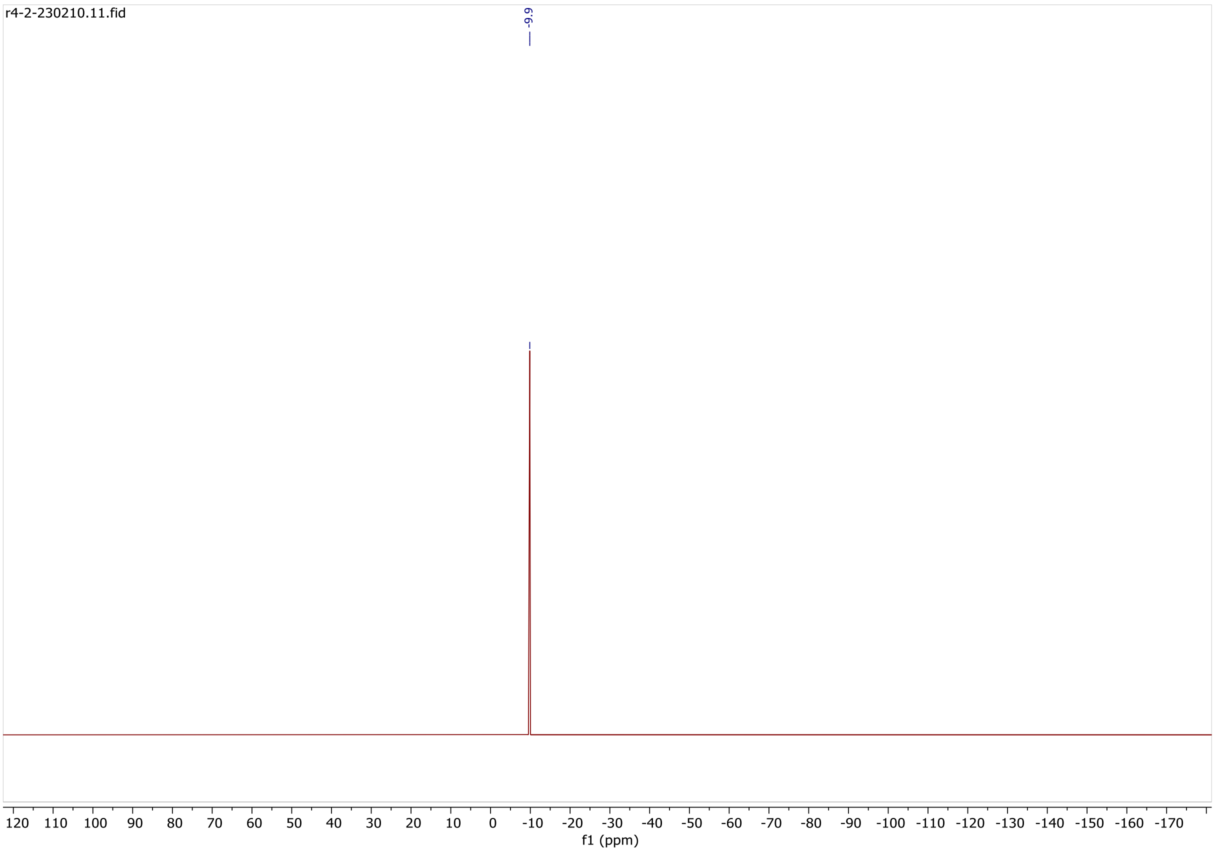

Compound 5b

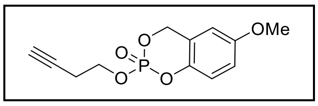

^{1}H NMR spectrum of **5b** (300 MHz, chloroform-d)

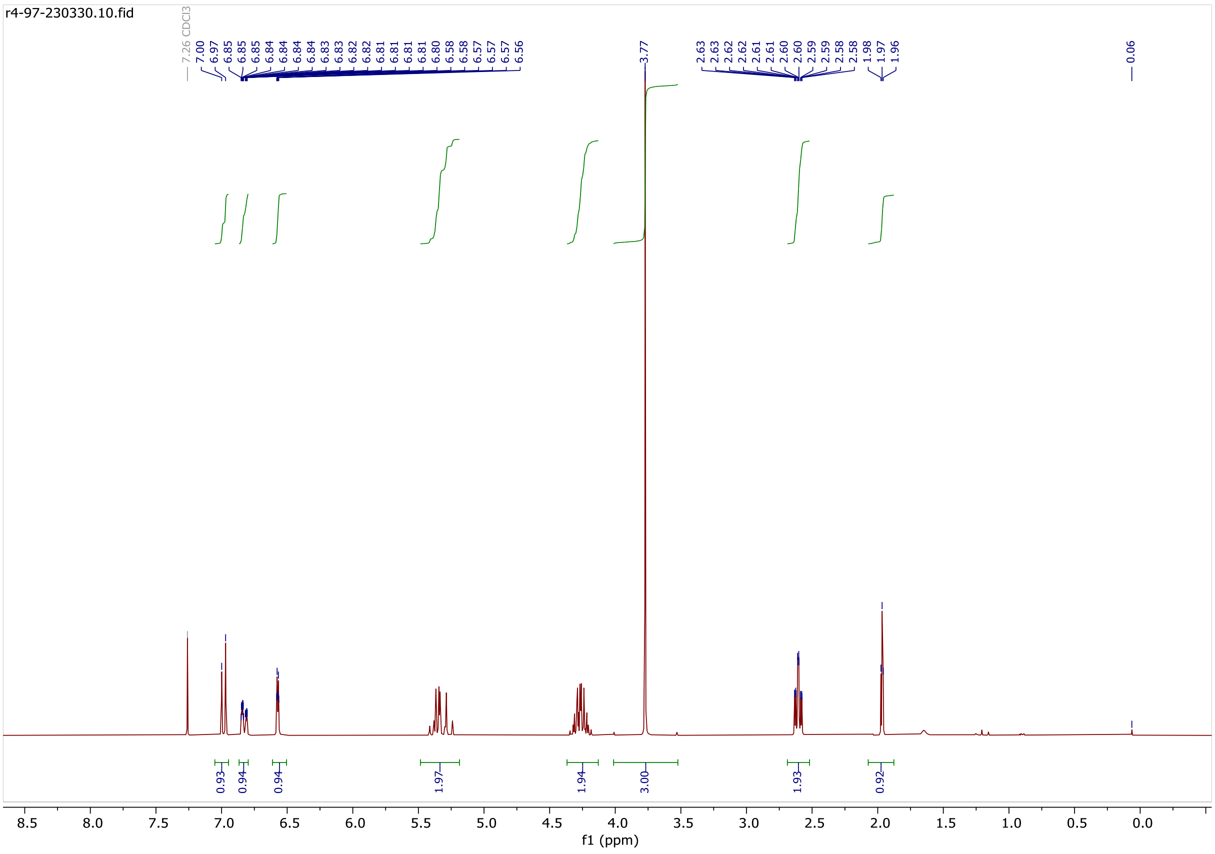

^{13}C NMR spectrum of **5b** (125.8 MHz, chloroform-d)

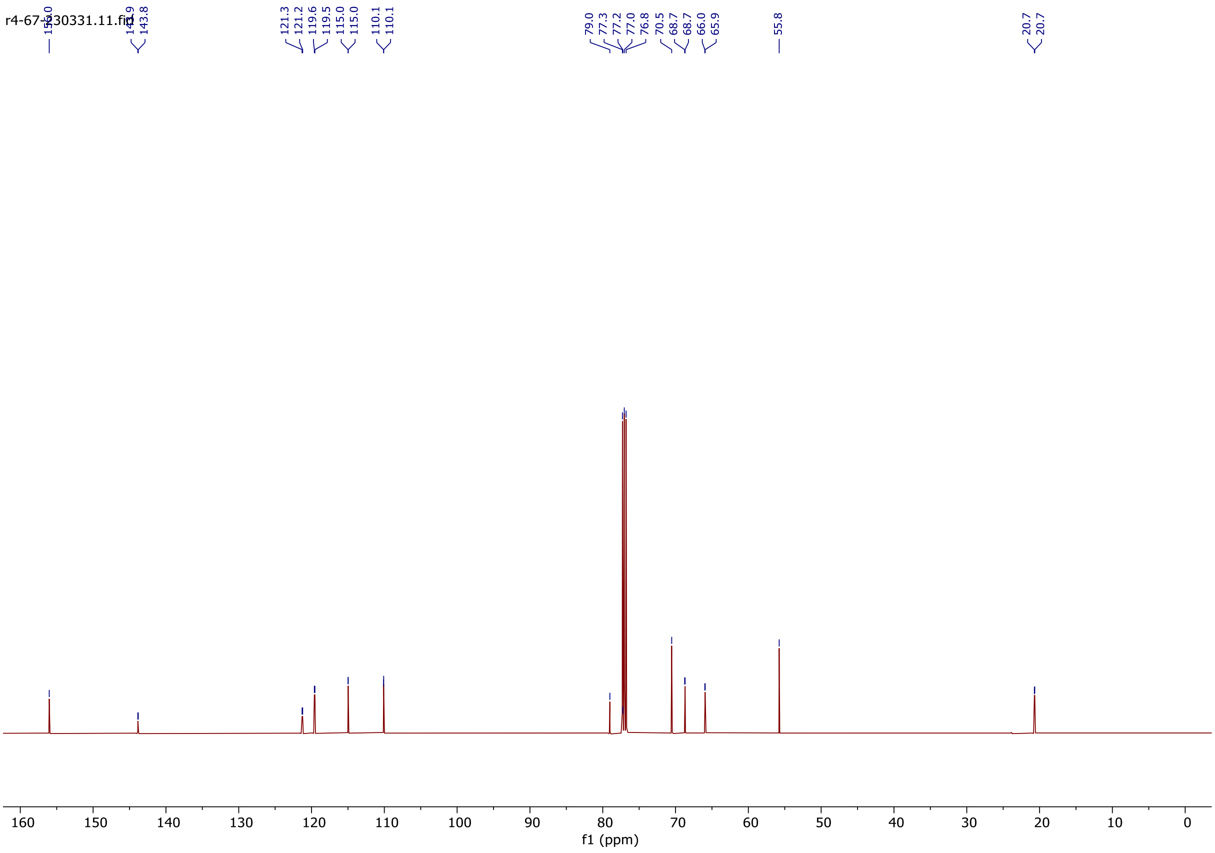

^{31}P NMR spectrum of **5b** (121.5 MHz, chloroform-d)

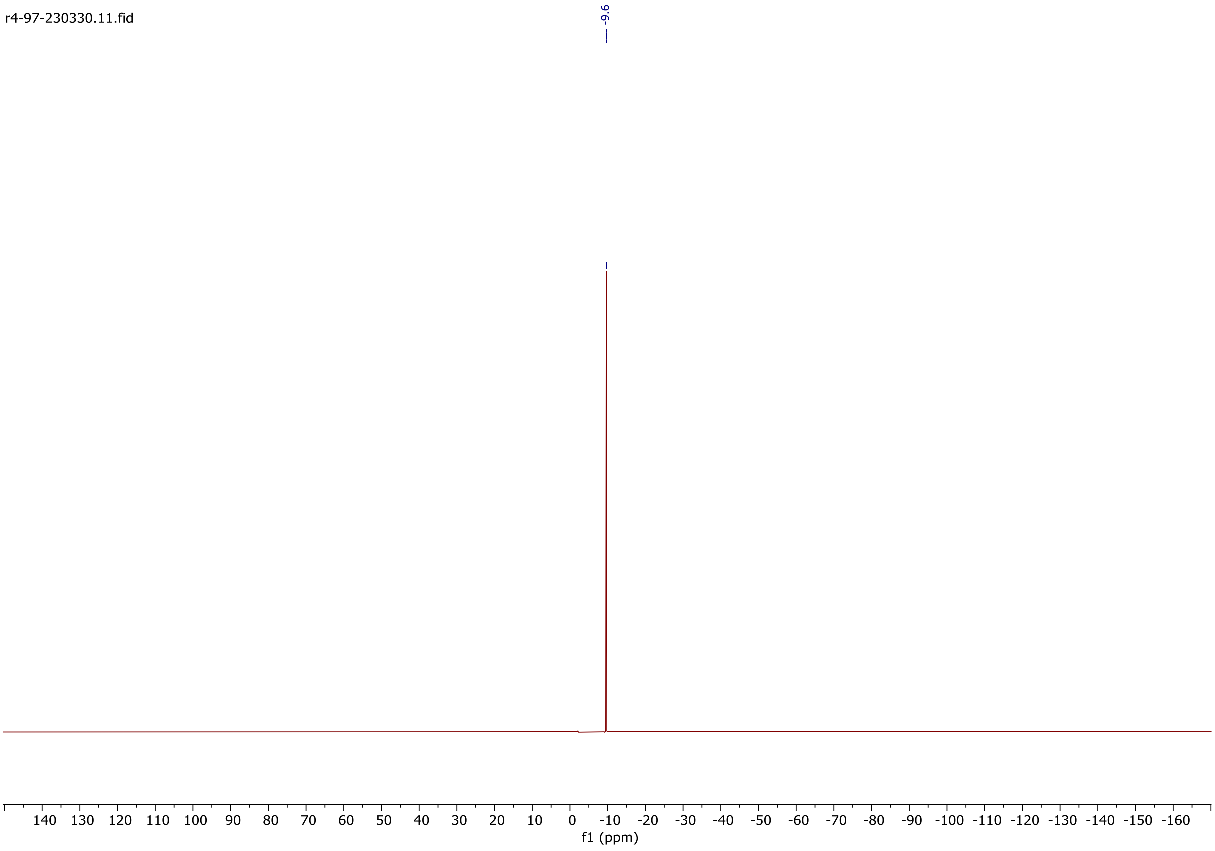

Compound 5c

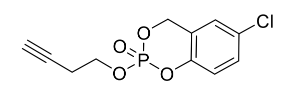

*^{1}H NMR spectrum of **5c** (300 MHz, chloroform-d)*

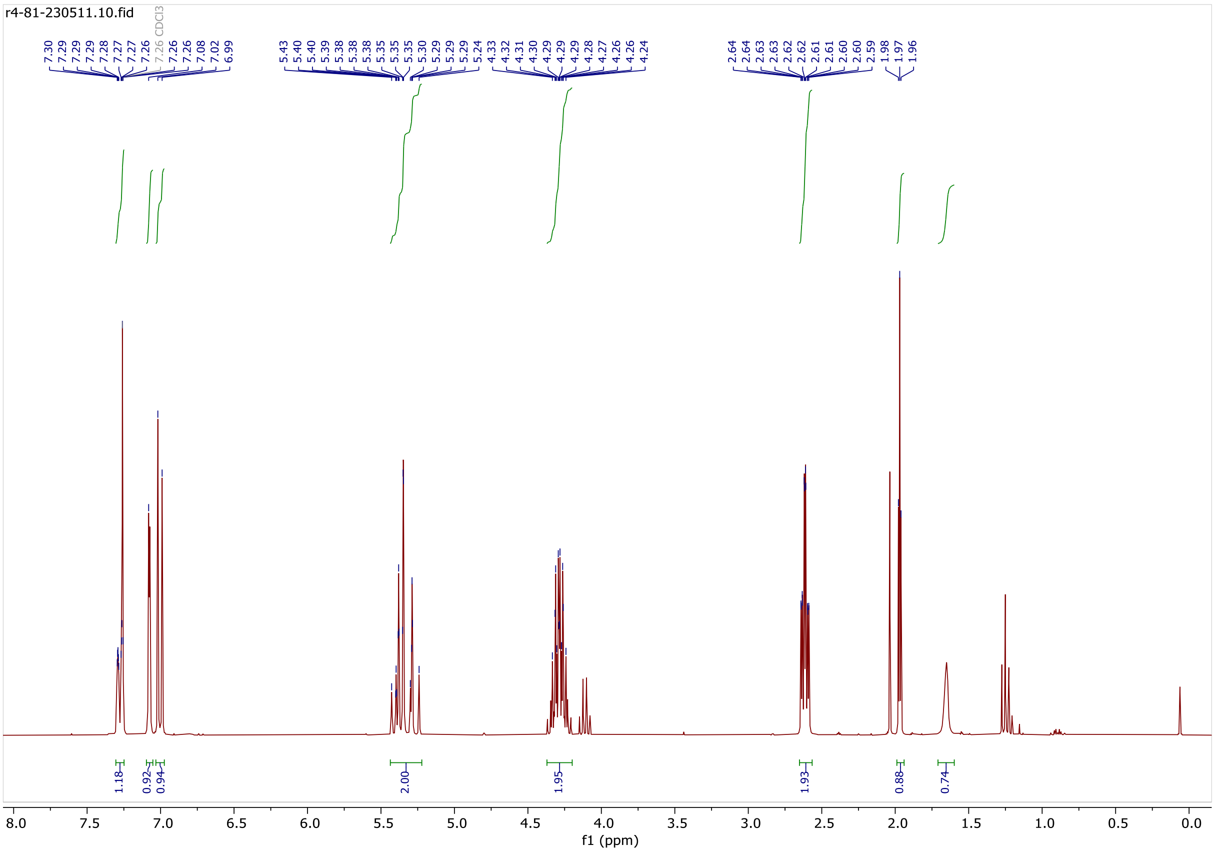

*^{13}C NMR spectrum of **5c** (125.8 MHz, chloroform-d)*

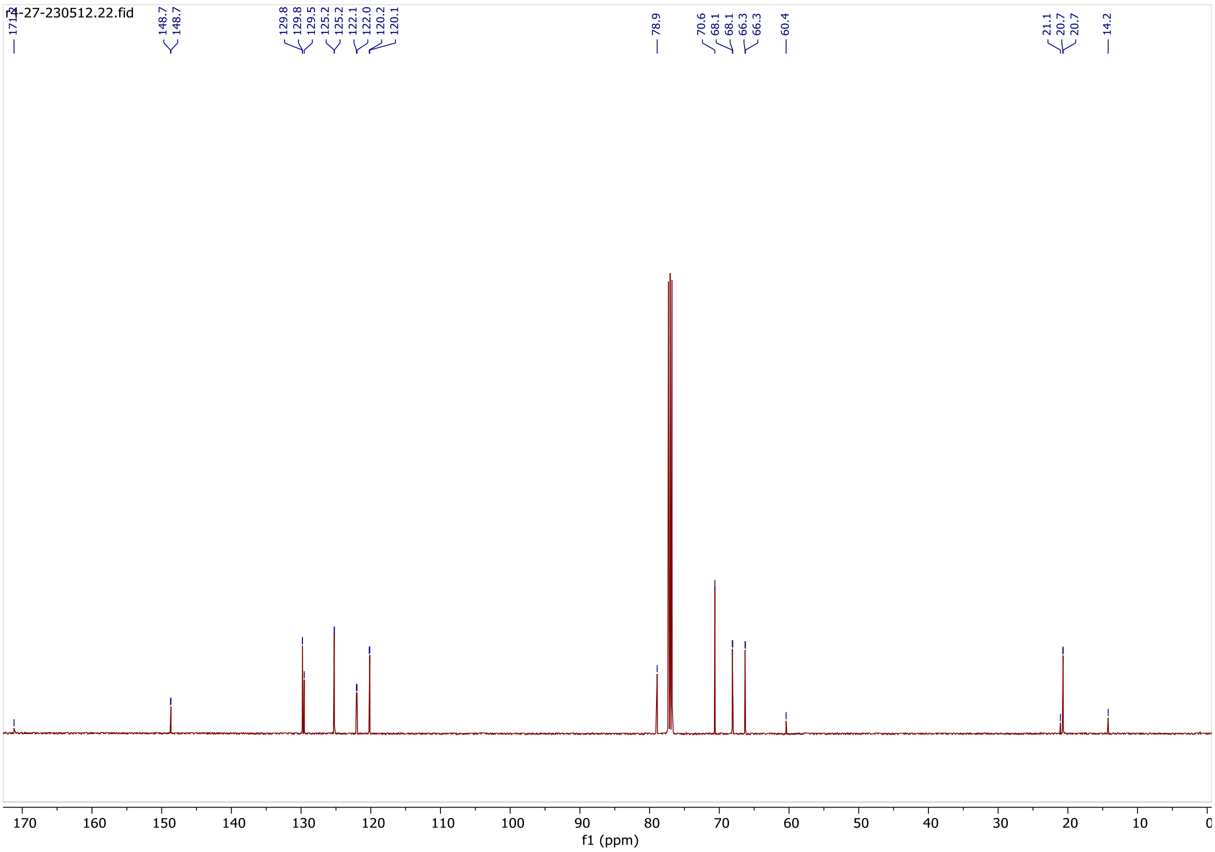

*^{31}P NMR spectrum of **5c** (121.5 MHz, chloroform-d)*

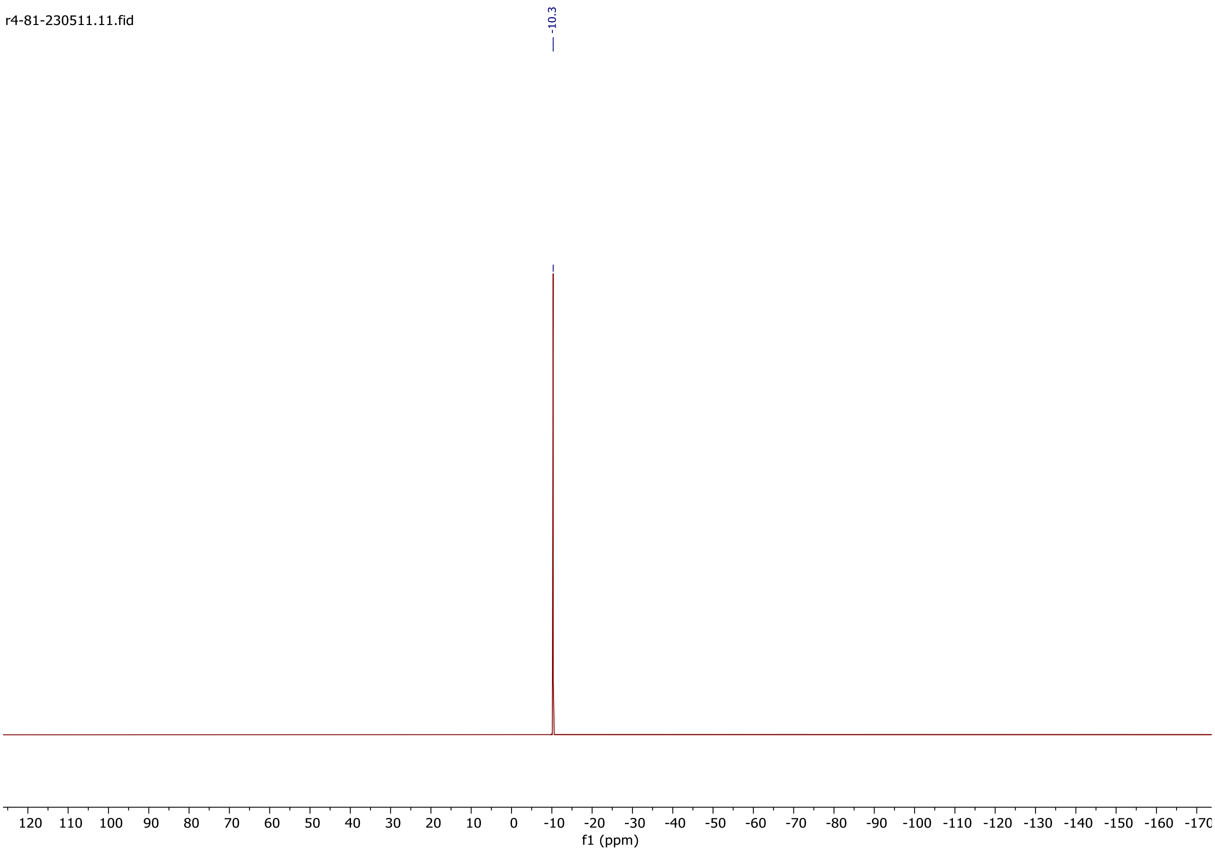

Compound 5d

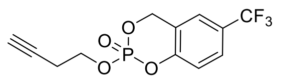

*^{1}H NMR spectrum of **5d** (300 MHz, chloroform-d)*

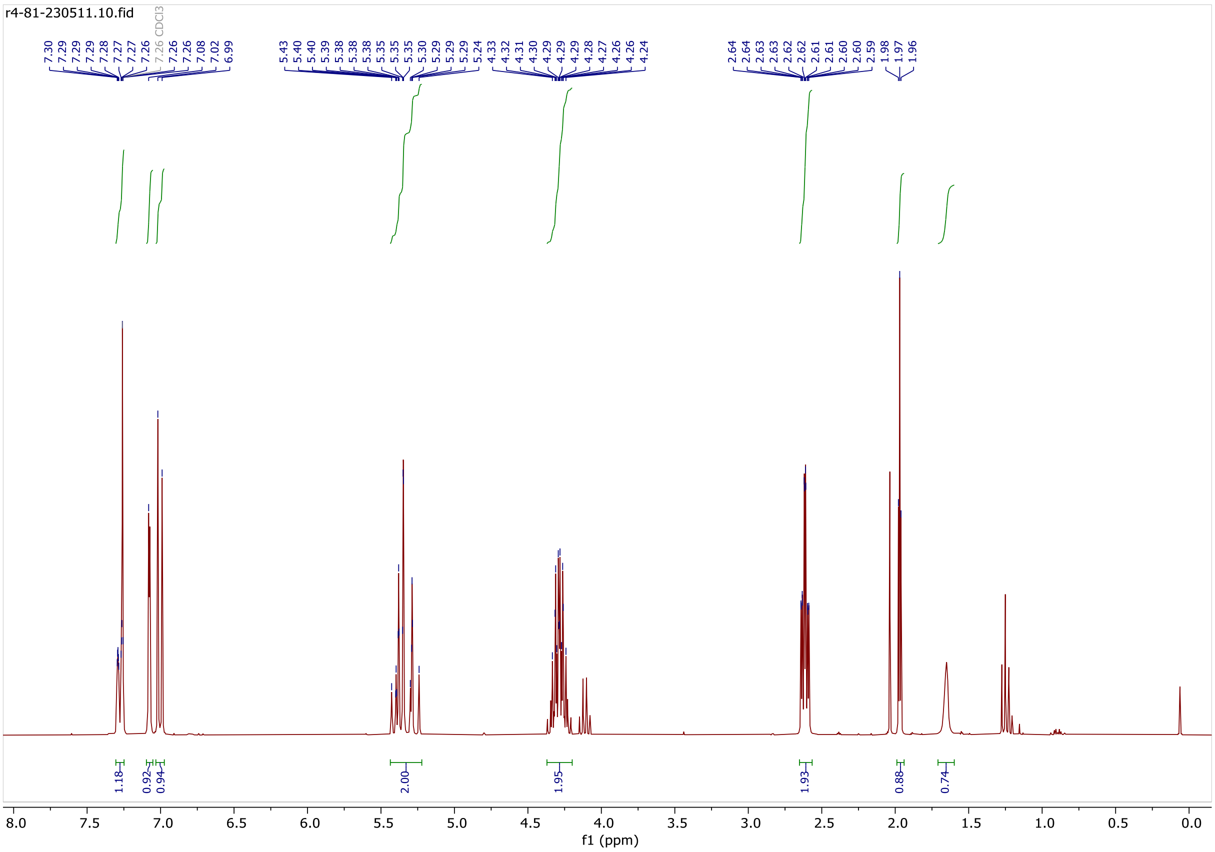

^{13}C NMR spectrum of **5d** (125.8 MHz, chloroform-d)

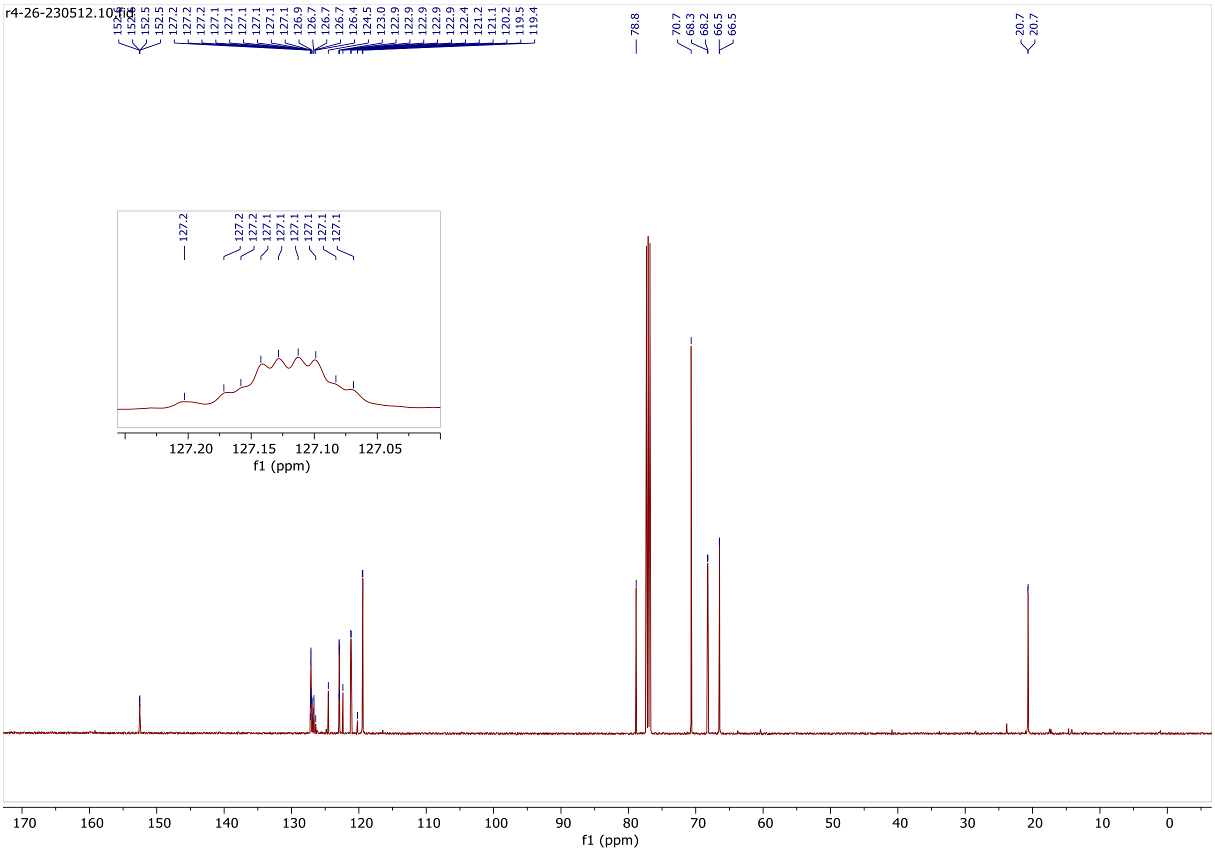

^{31}P NMR spectrum of **5d** (121.5 MHz, chloroform-d)

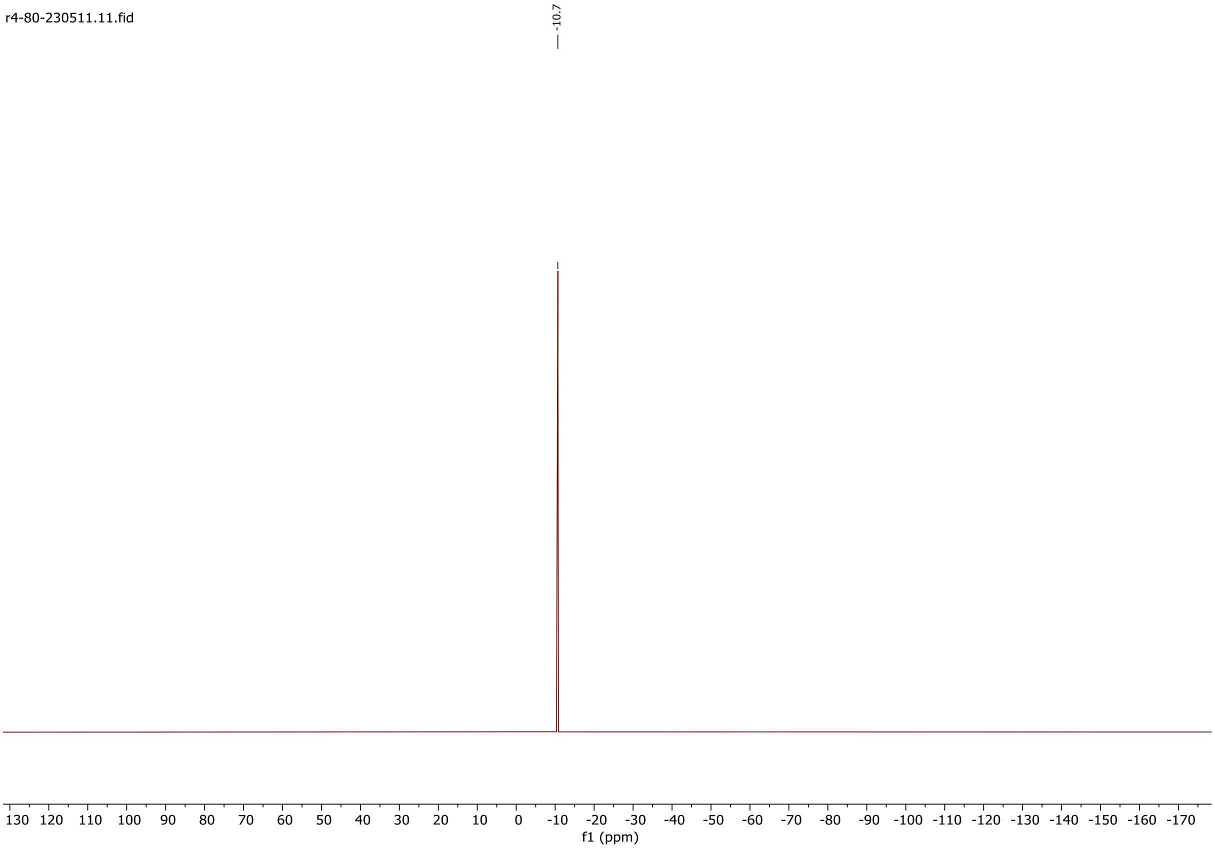

^{19}F *NMR spectrum of* ***5d*** *(282.4 MHz, chloroform-d)*

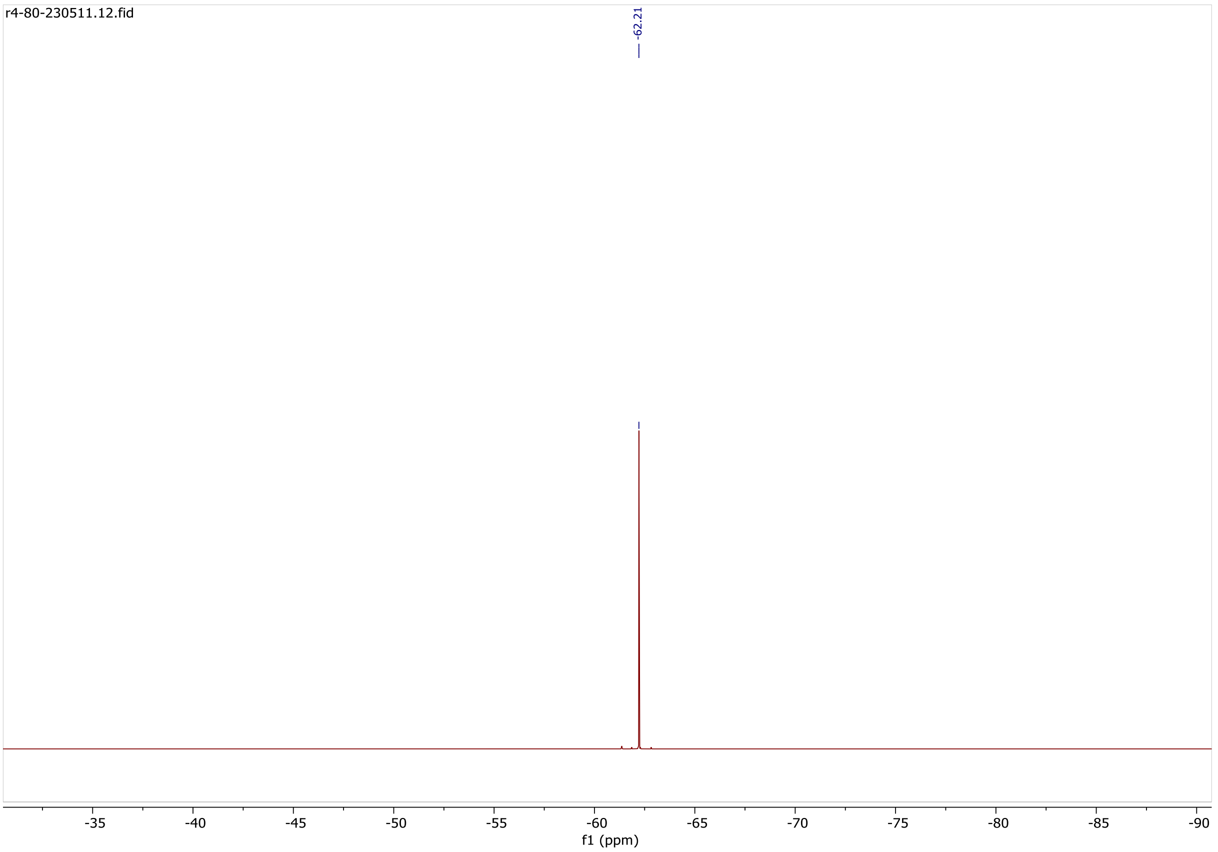

Compound 5e

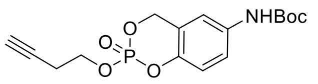

^{1}H *NMR spectrum of* ***5e*** *(500 MHz, chloroform-d)*

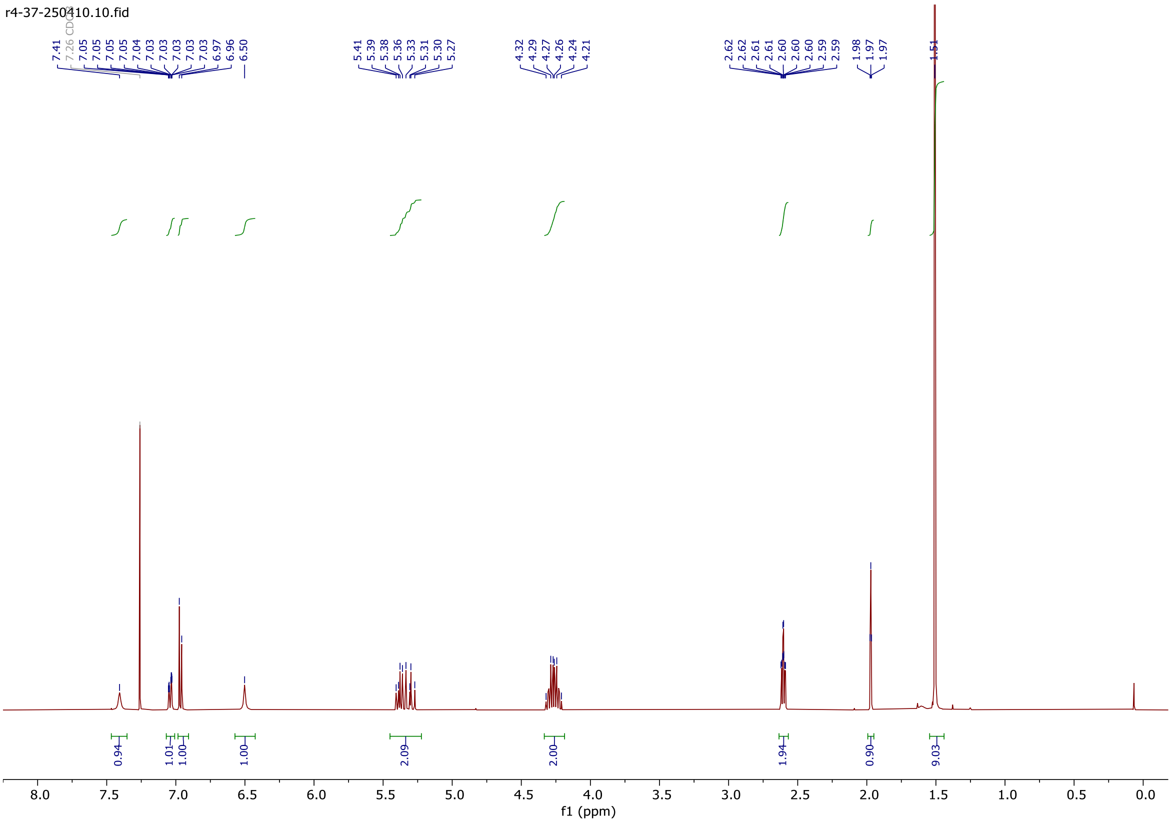

^{13}C NMR spectrum of **5e** (125.8 MHz, chloroform-d)

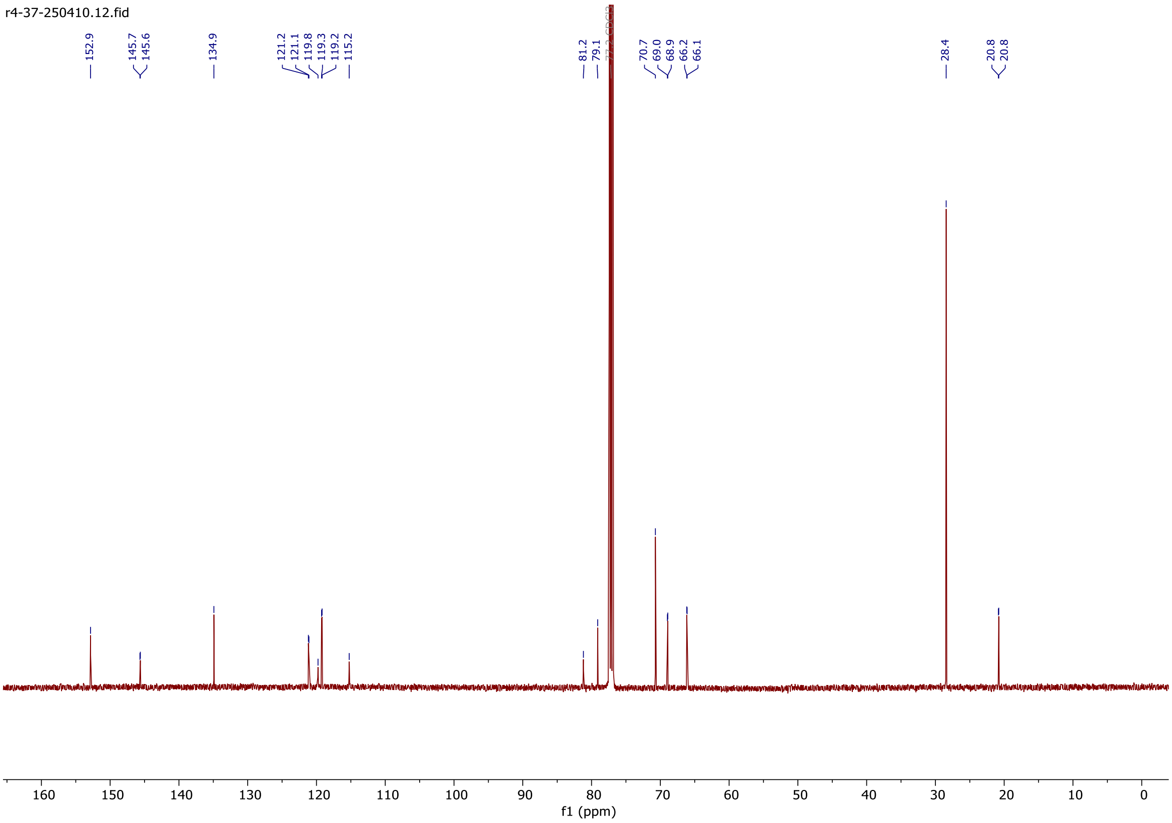

^{31}P NMR spectrum of **5e** (202.4 MHz, chloroform-d)

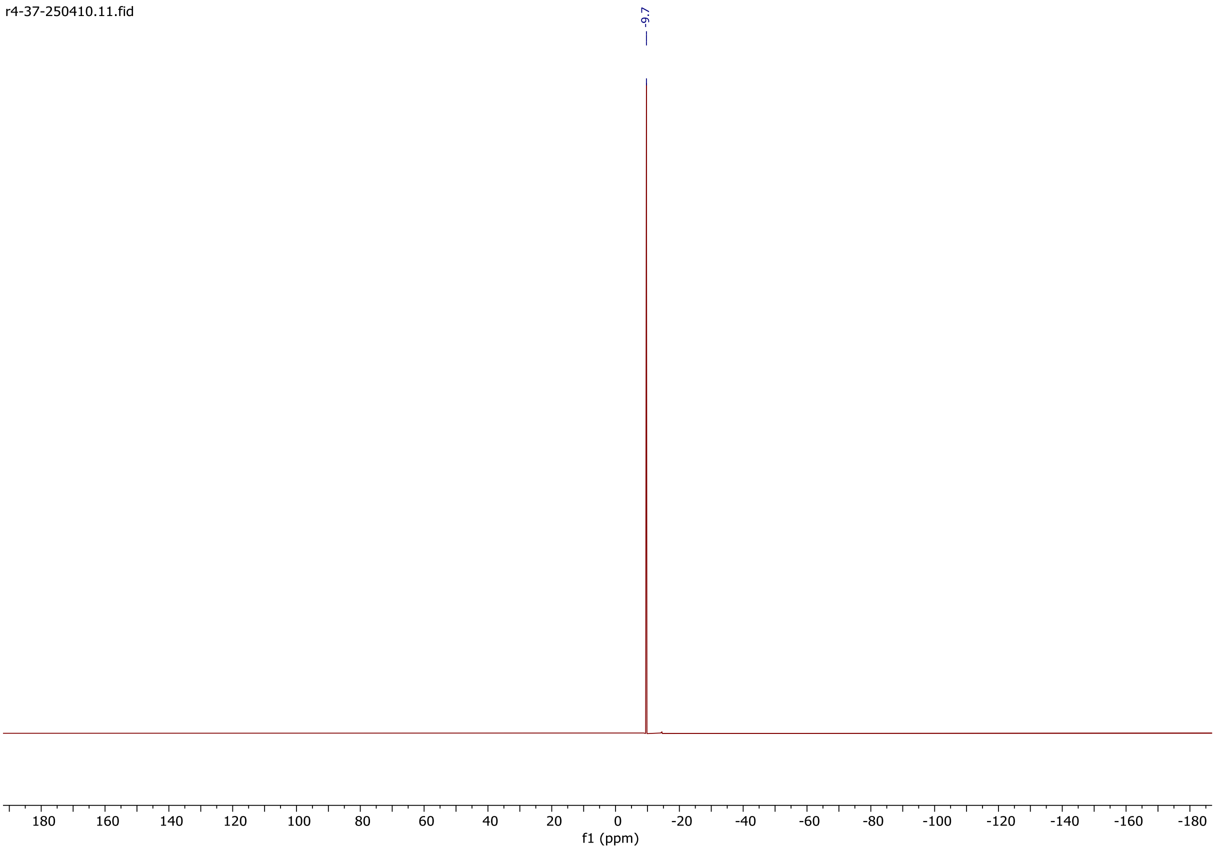

Compound 5f

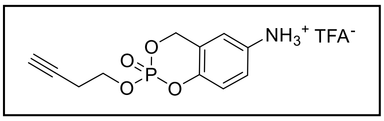

*^{1}H NMR spectrum of **5f** (500 MHz, methanol-d4)*

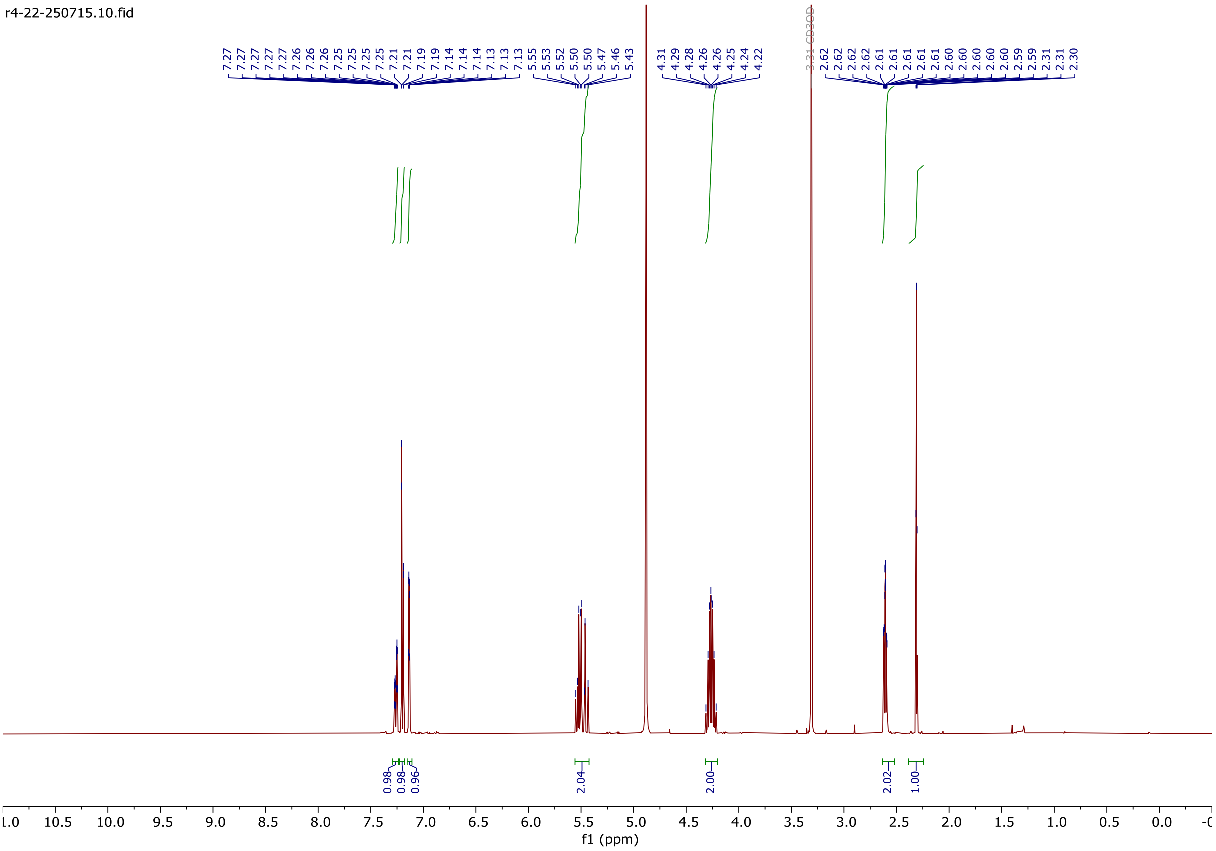

*^{13}C NMR spectra of **5f** (125.8 MHz, methanol-d4)*

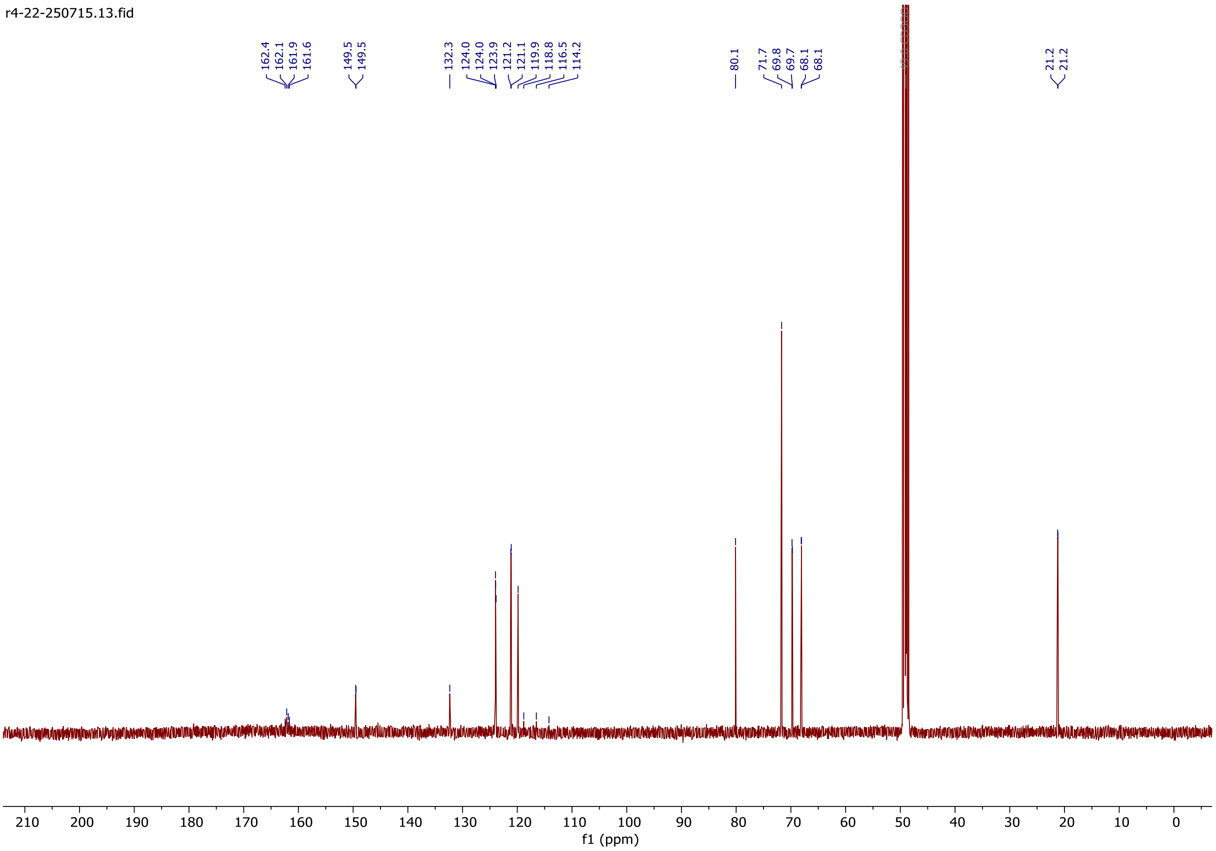

*^{31}P NMR spectrum of **5f** (202.4 MHz, methanol-d4)*

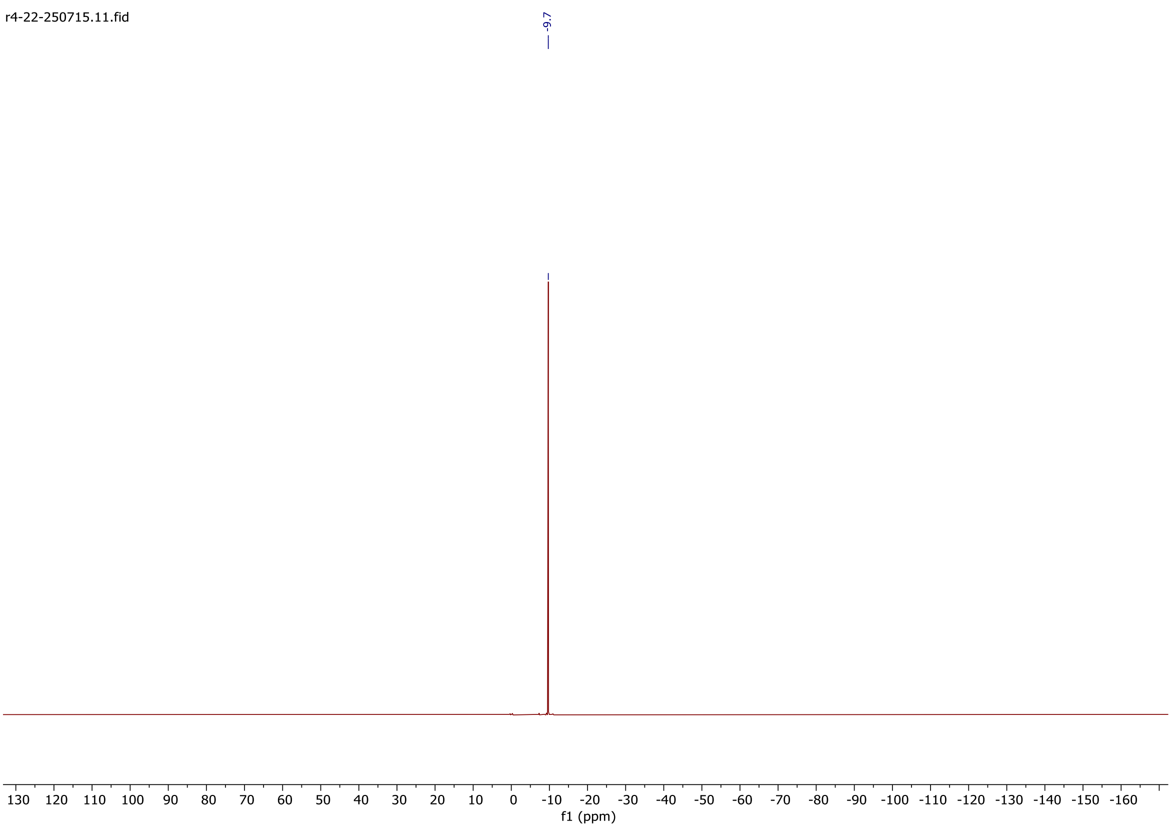

*^{19}F NMR spectra of **5f** (470.5 MHz, methanol-d4)*

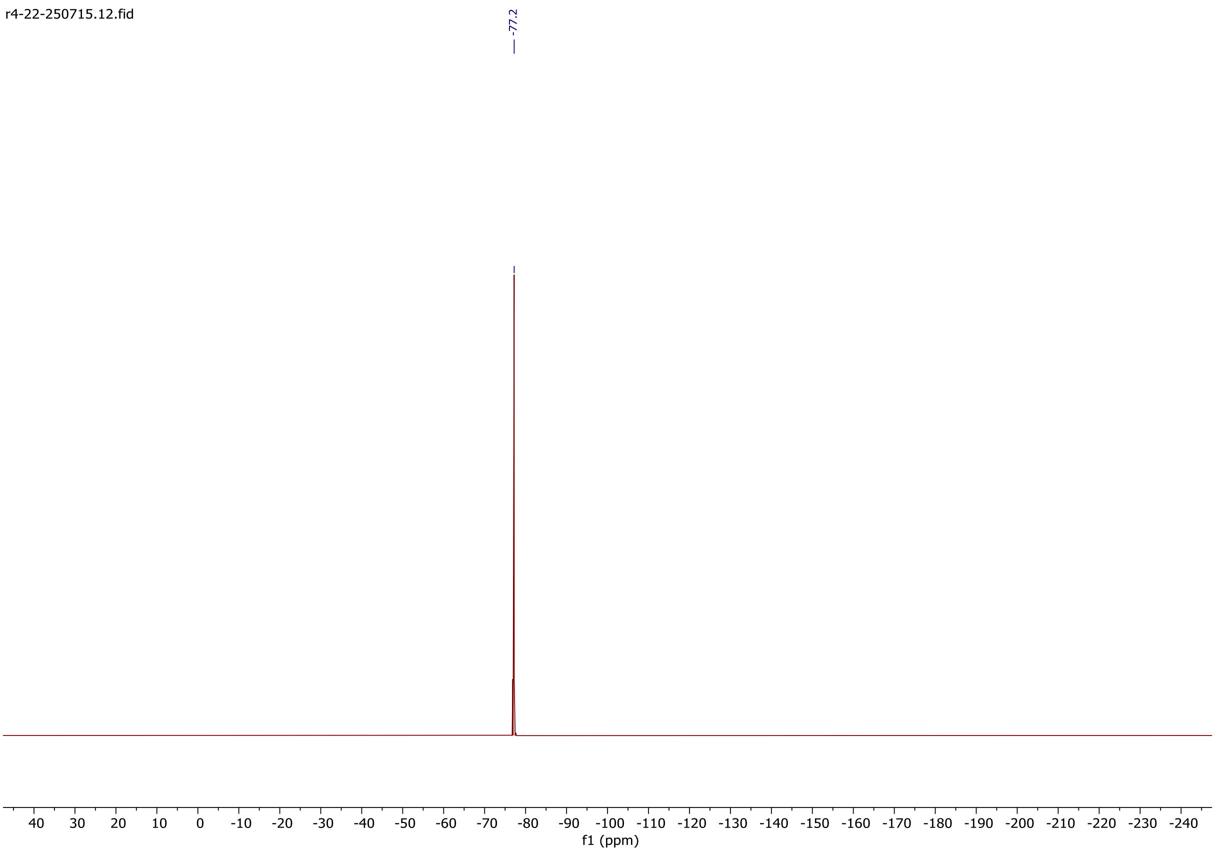

Compound 5g

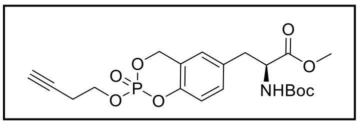

*^{1}H NMR spectrum of **5g** (300 MHz, chloroform-d)*

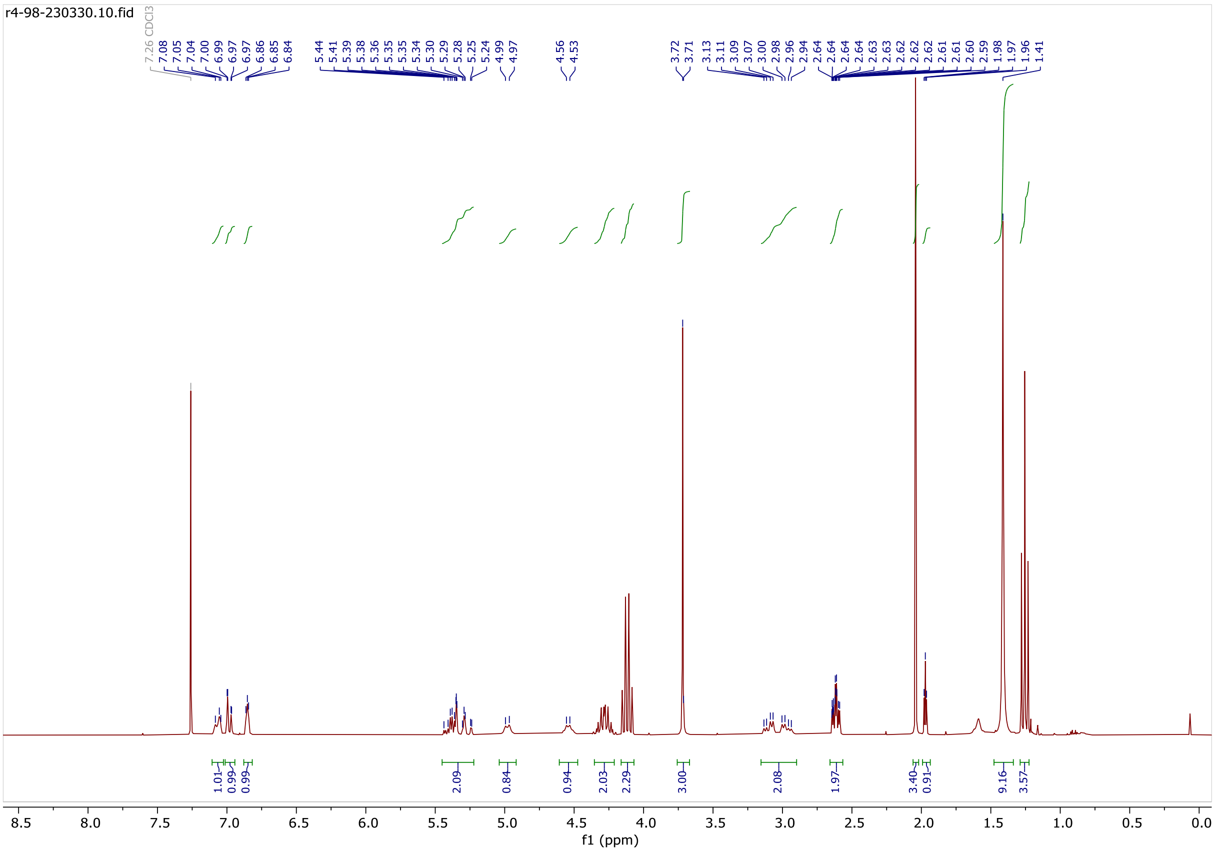

*^{13}C NMR spectrum of **5g** (125.8 MHz, chloroform-d)*

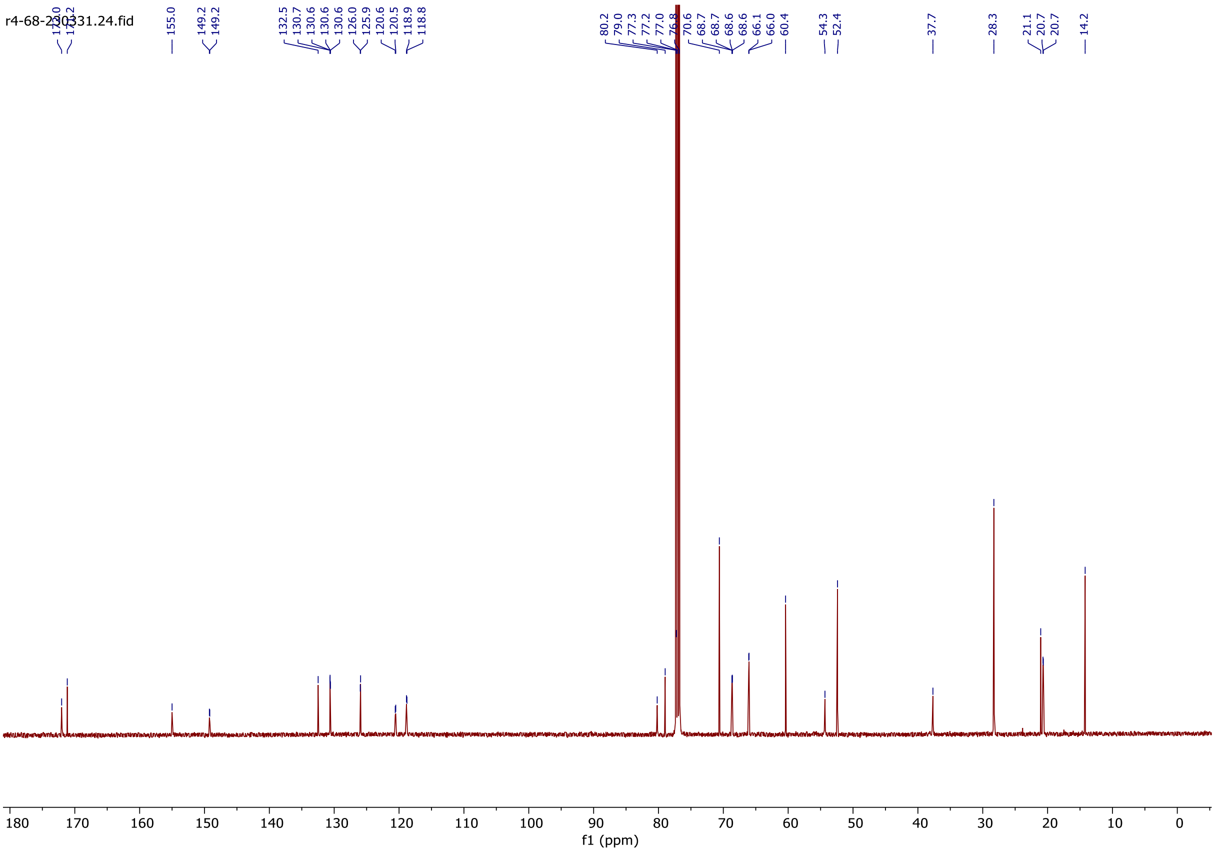

*^{31}P NMR spectrum of **5g** (121.5 MHz, chloroform-d)*

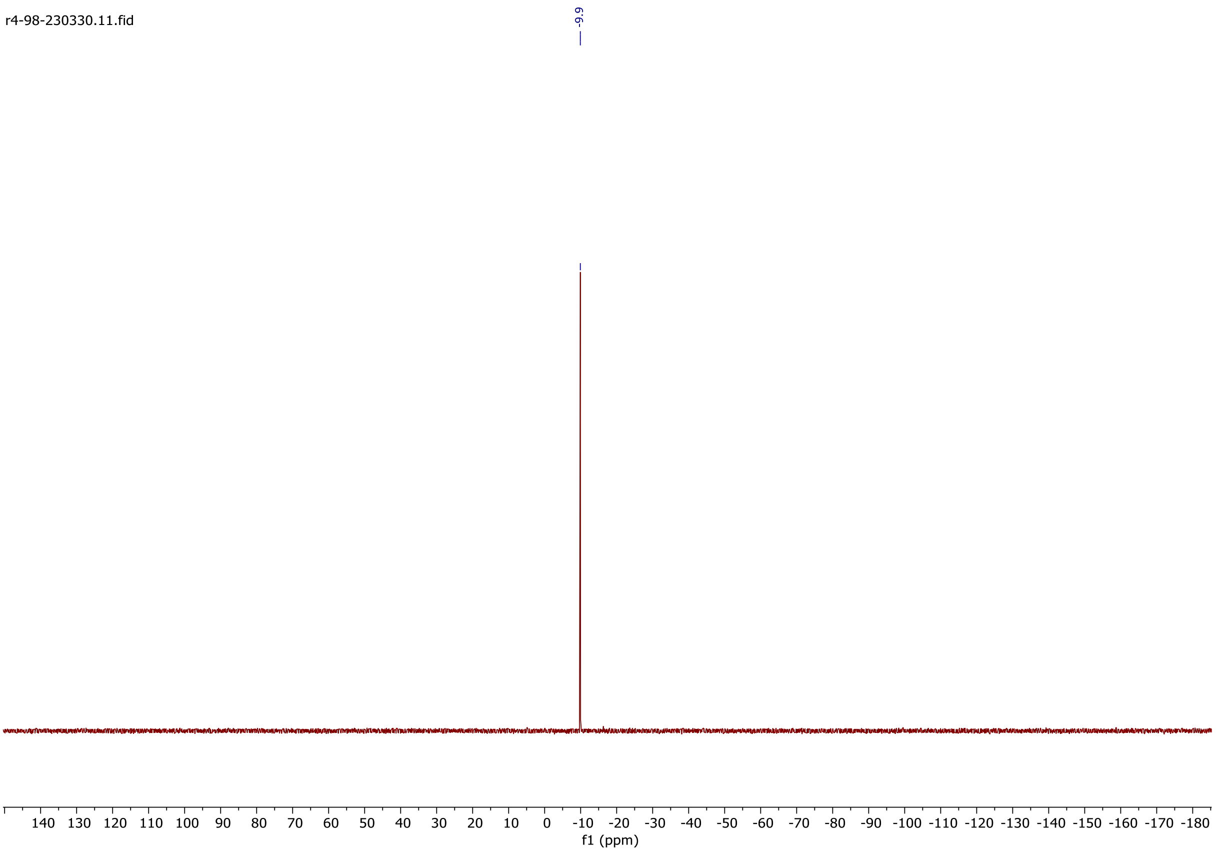

Compound 5h

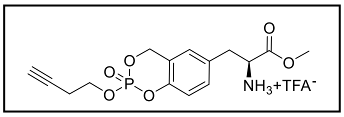

*^{1}H NMR spectrum of **5h** (500 MHz, methanol-d4)*

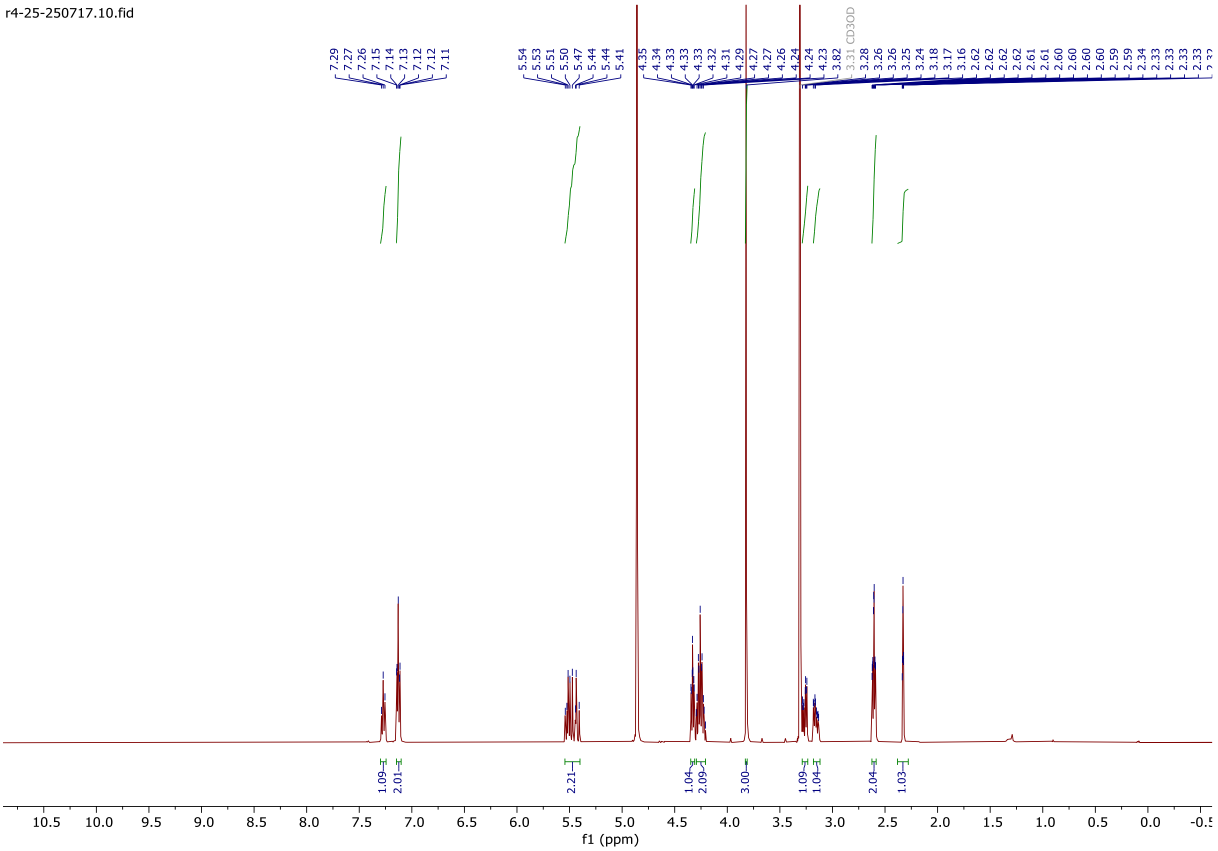

^{31}*P NMR spectrum of* ***5h*** *(121.5 MHz, methanol-d4)*

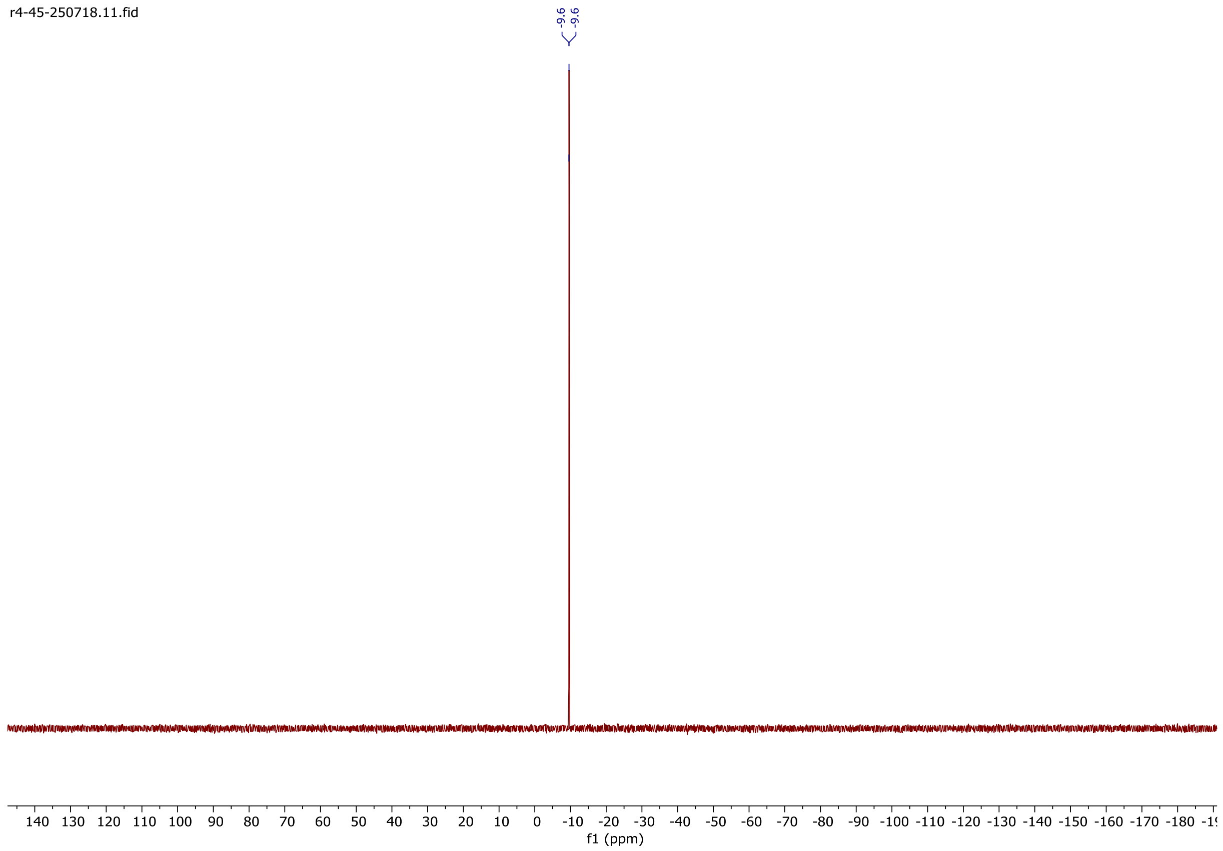

^{19}*F NMR spectrum of* ***5h*** *(282.4 MHz, methanol-d4)*

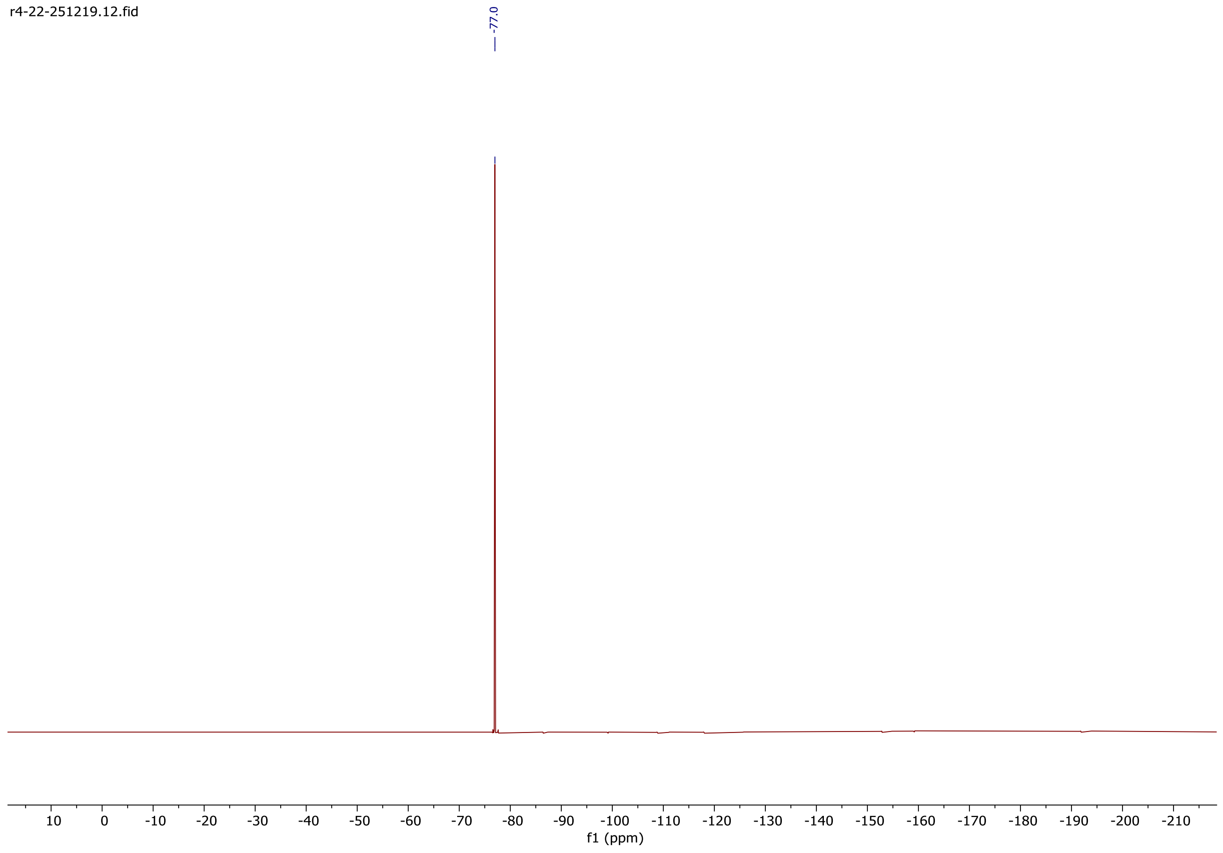

*^{13}C NMR spectra of **5h** (125.8 MHz, methanol-d4)*

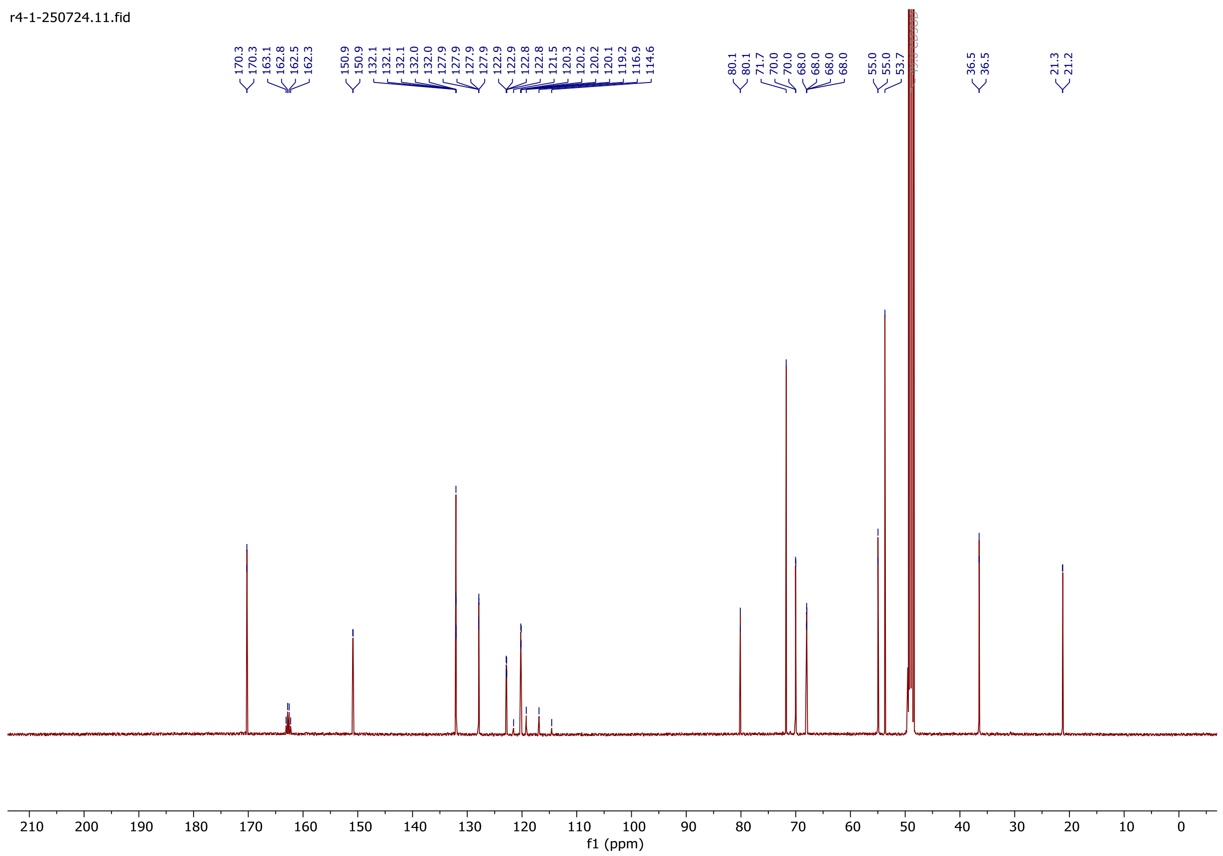

Compound 6a

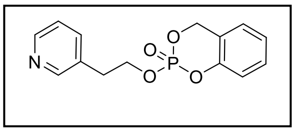

*^{1}H NMR spectrum of **6a** (500 MHz, chloroform-d)*

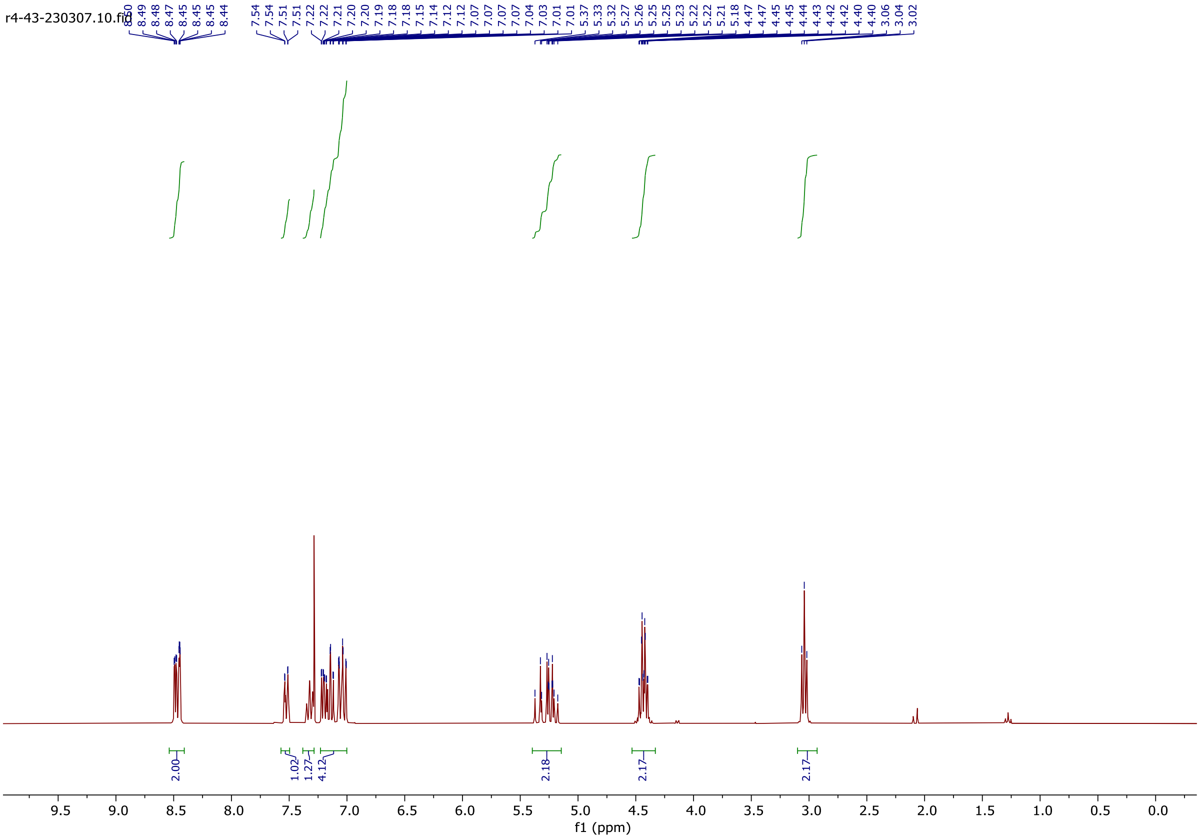

*^{13}C NMR spectra of **6a** (125.8 MHz, chloroform-d)*

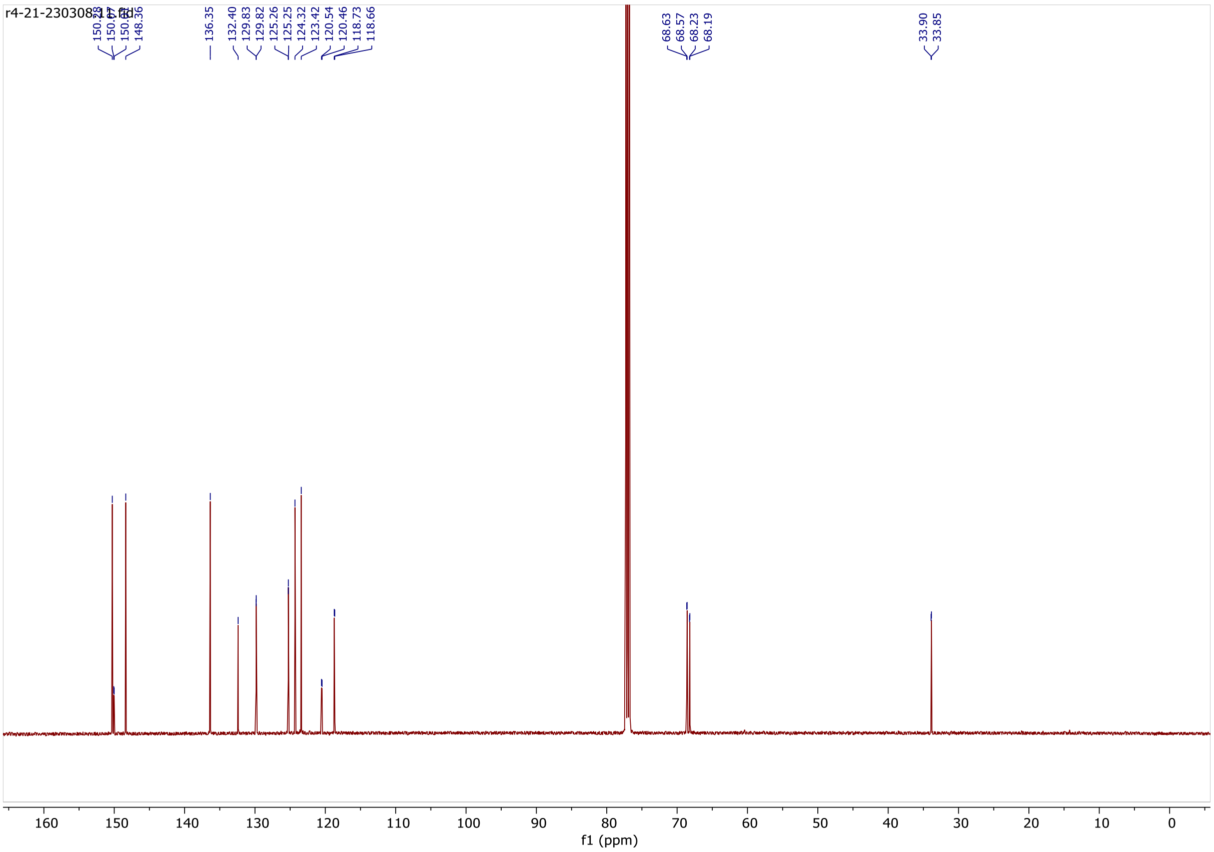

*^{31}P NMR spectrum of **6a** (121.5 MHz, chloroform-d)*

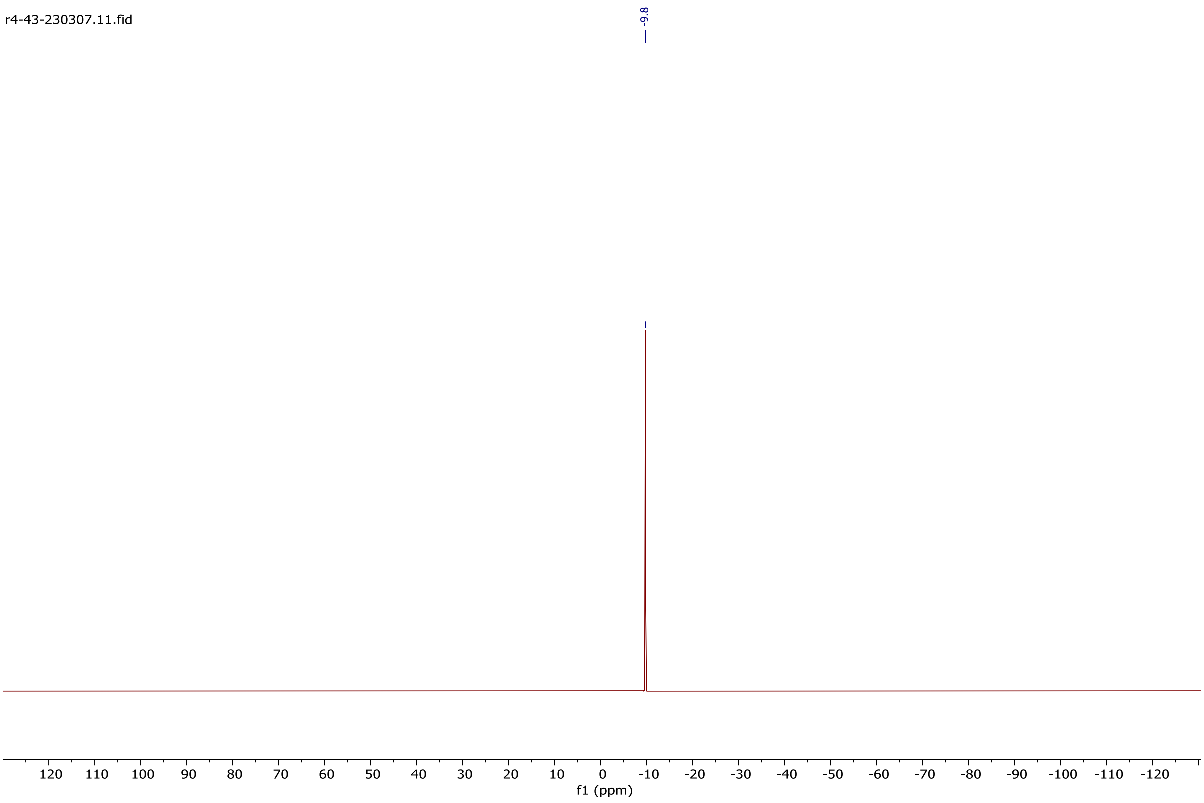

Compound 6b

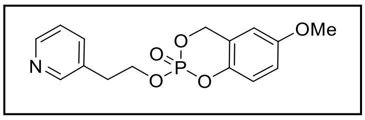

*^{1}H NMR spectrum of **6b** (300 MHz, chloroform-d)*

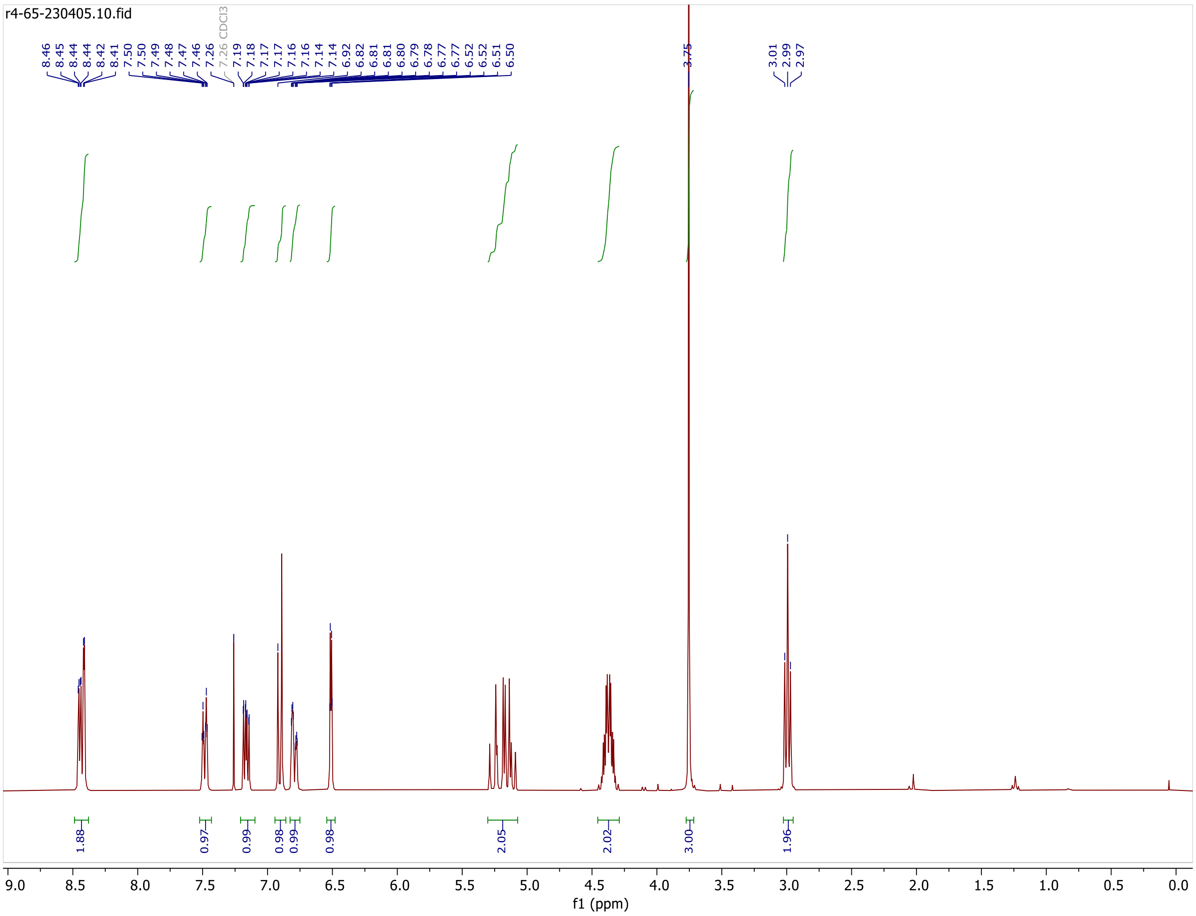

^{13}C NMR spectra of **6b** (125.8 MHz, chloroform-d)

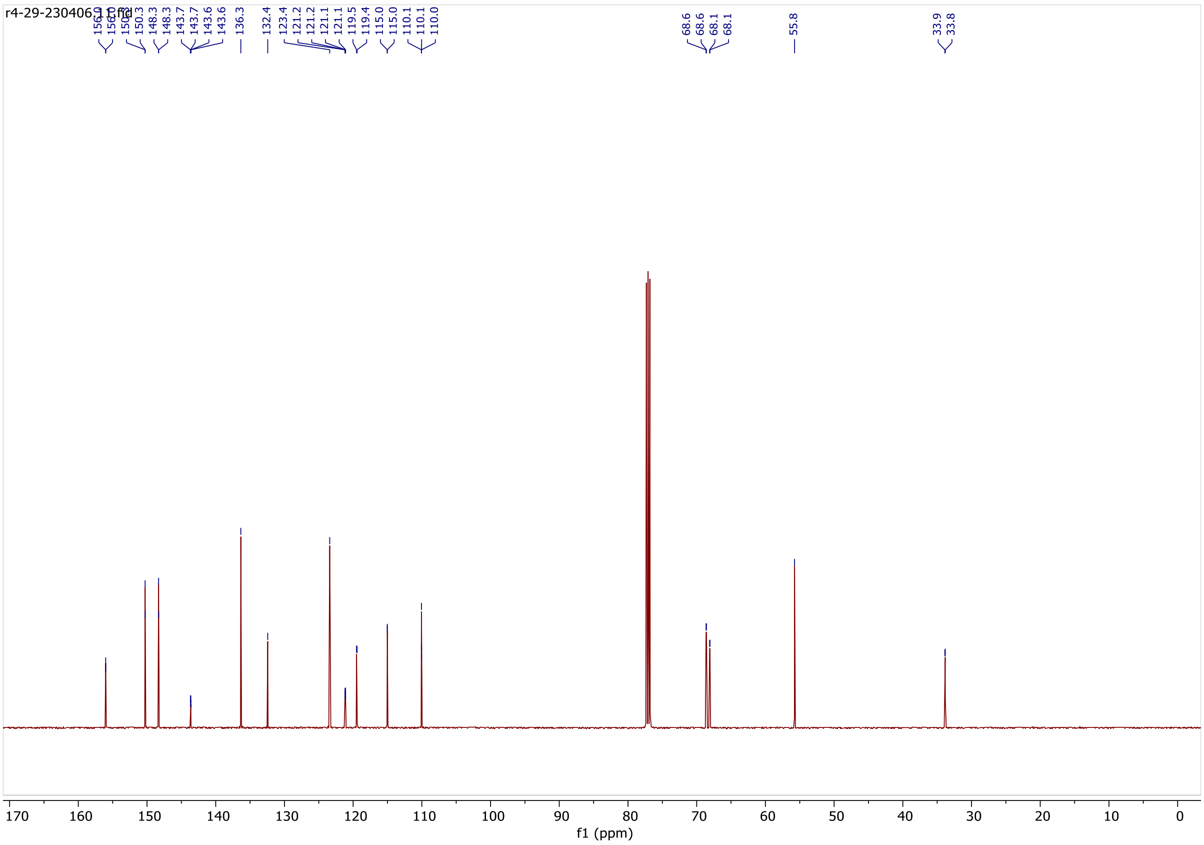

^{31}P NMR spectrum of **6b** (121.5 MHz, chloroform-d)

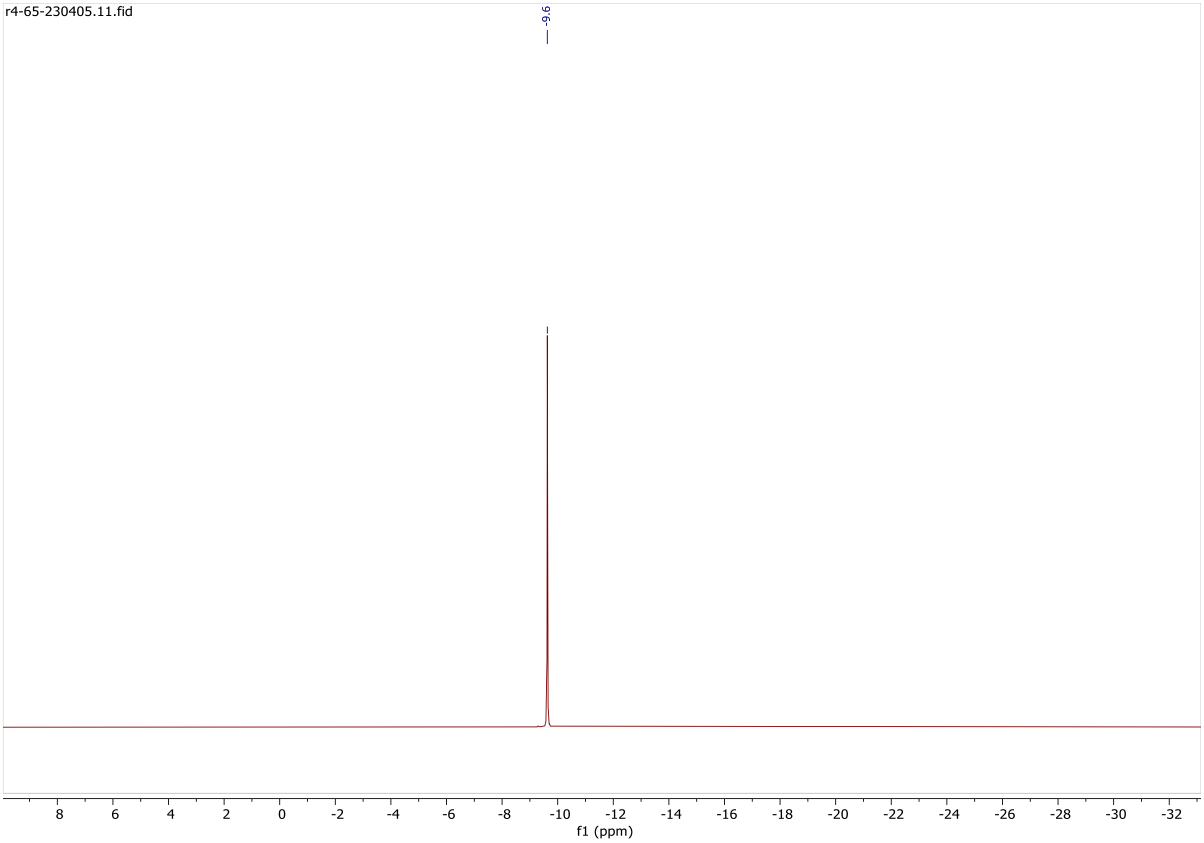

Compound 6c

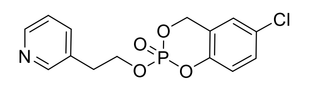

^{1}H NMR spectrum of ***6c*** *(300 MHz, chloroform-d)*

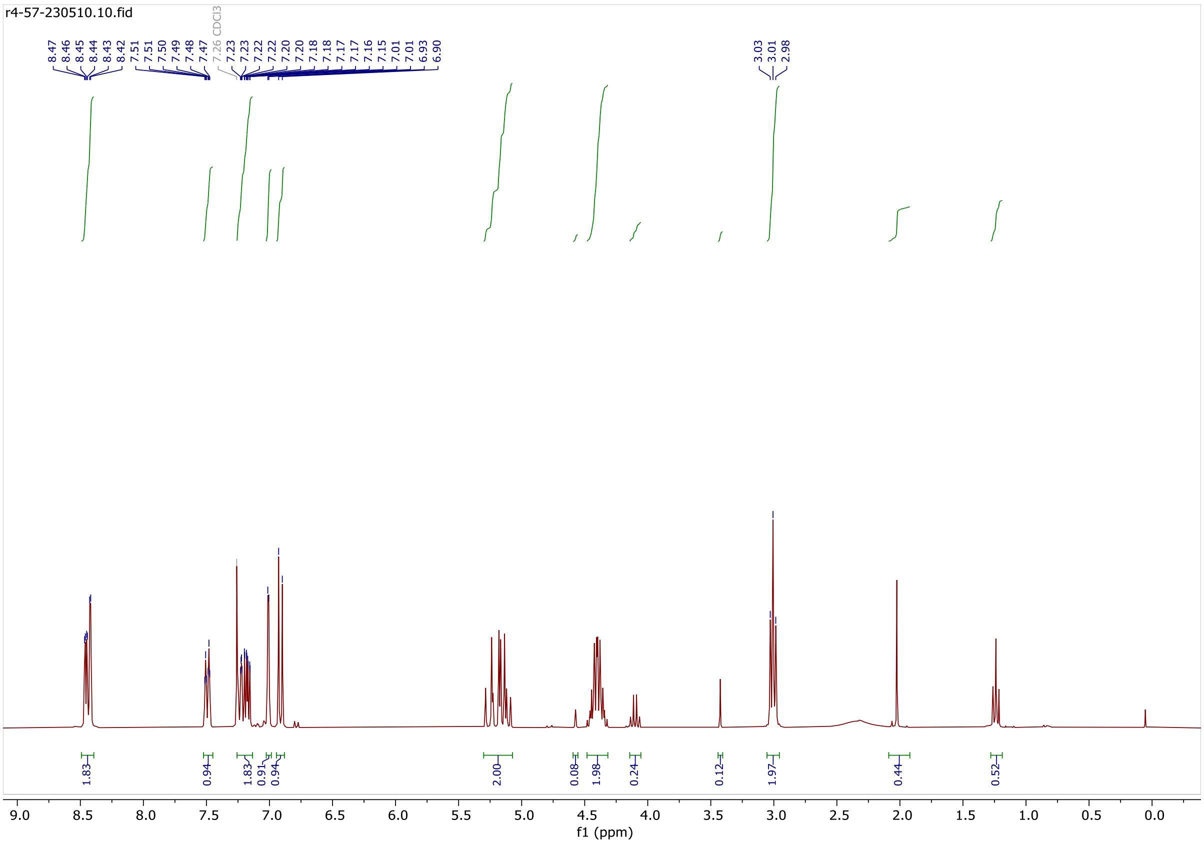

^{13}C NMR spectra of ***6c*** *(125.8 MHz, chloroform-d)*

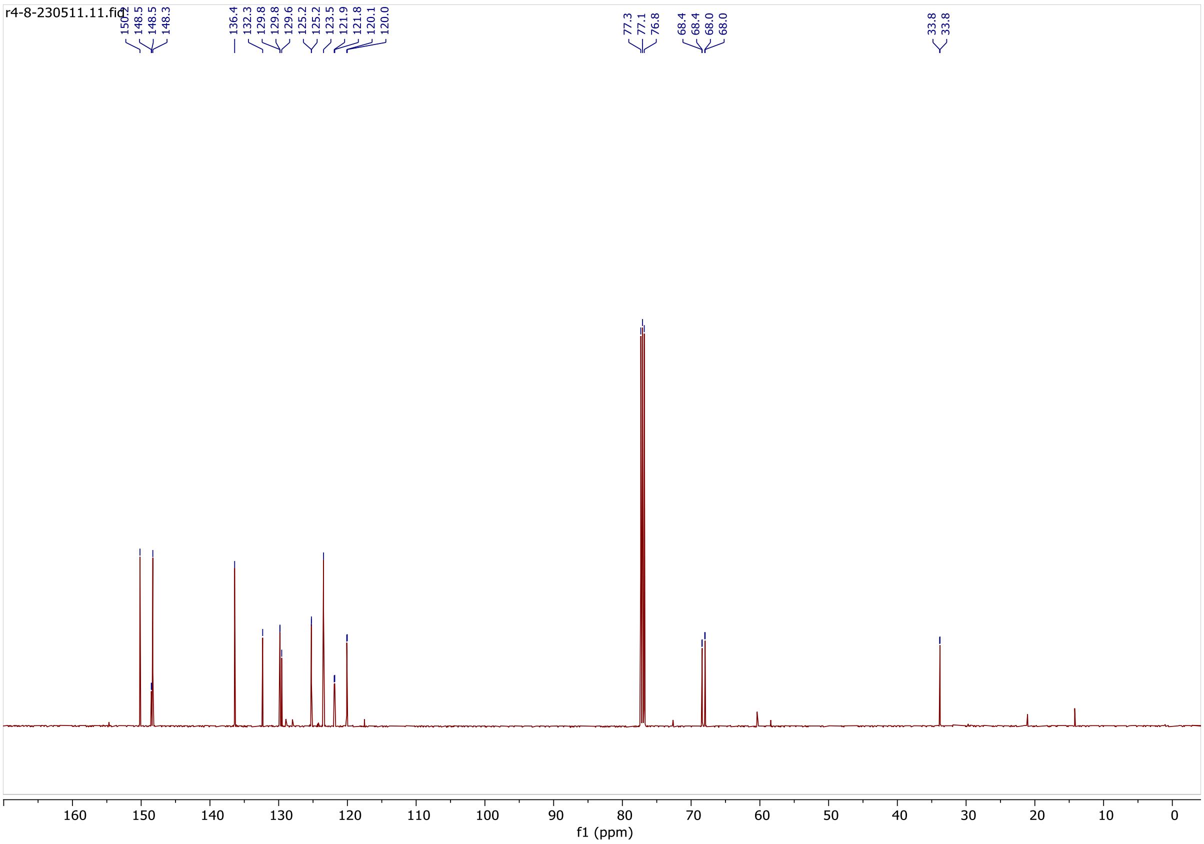

*[31] P NMR spectrum of **6c** (121.5 MHz, chloroform-d)*

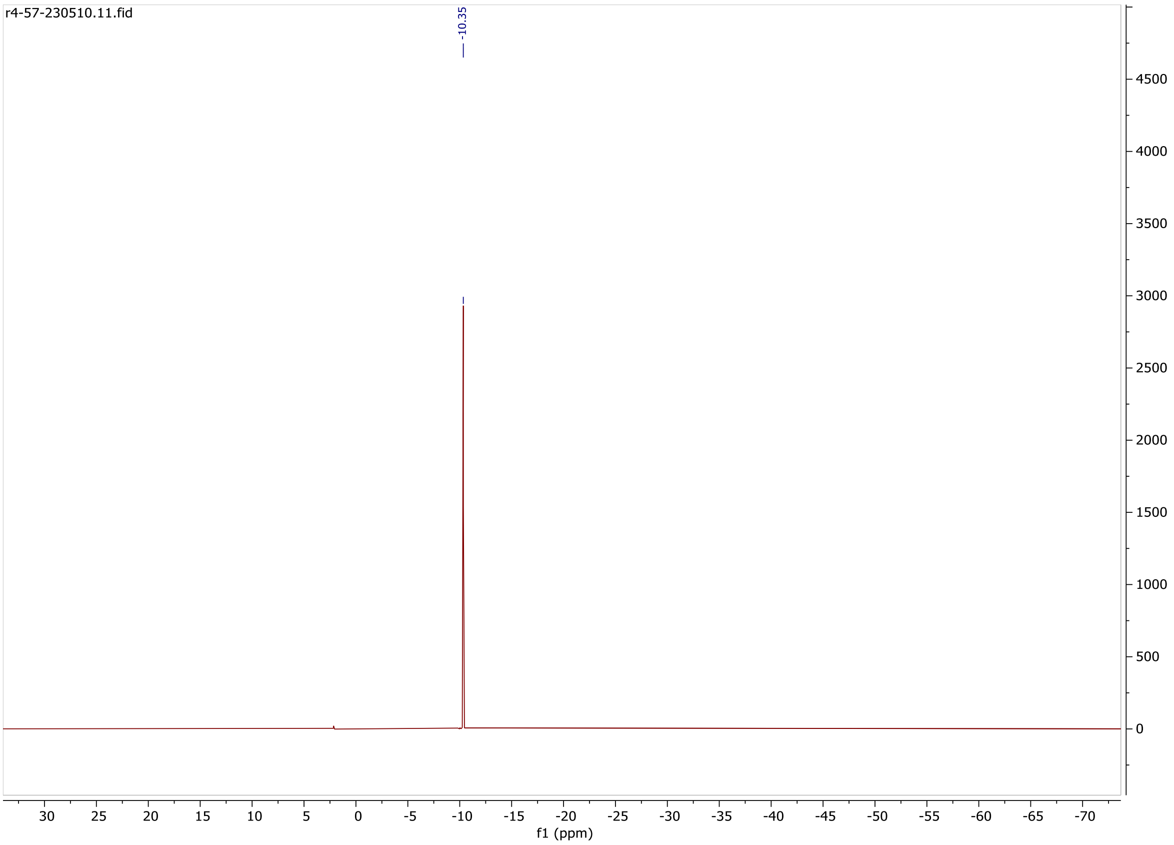

Compound 6d

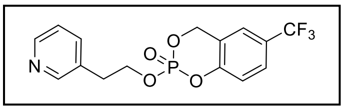

*[1] H NMR spectrum of **6d** (400 MHz, chloroform-d)*

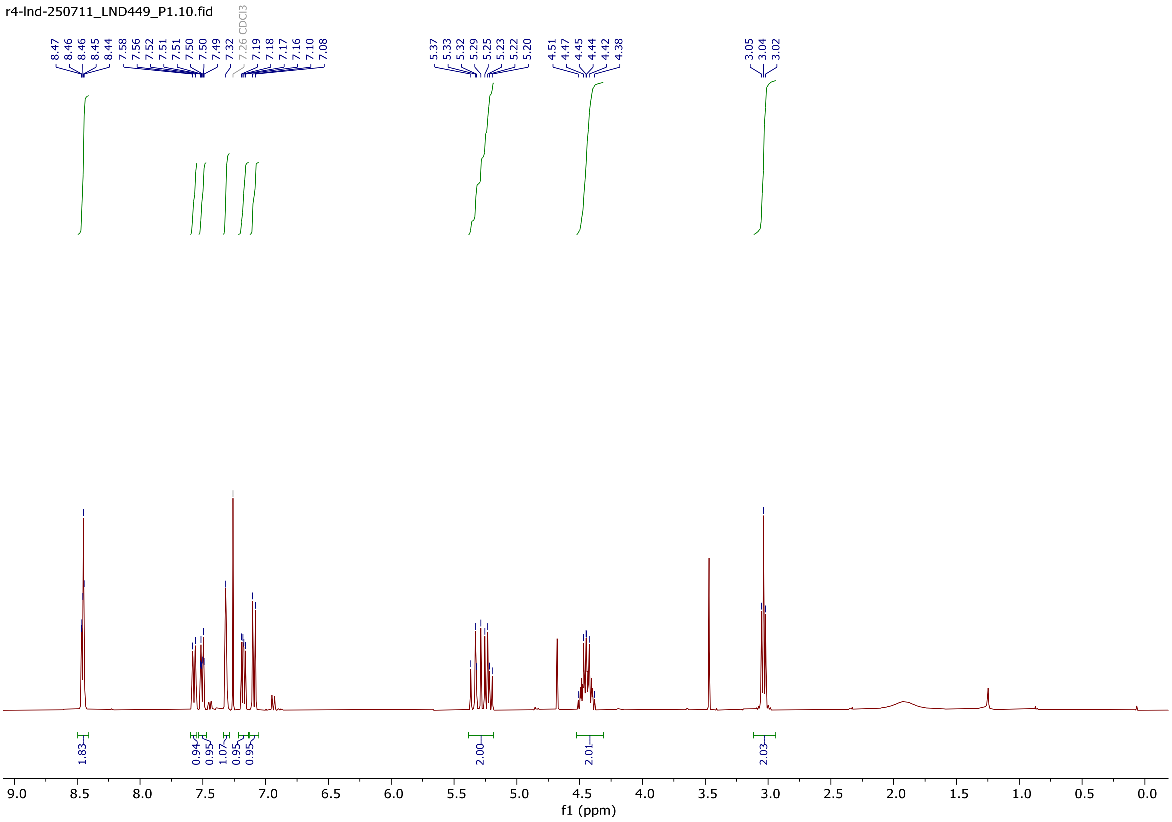

^{13}C NMR spectra of **6d** (125.8 MHz, chloroform-d)

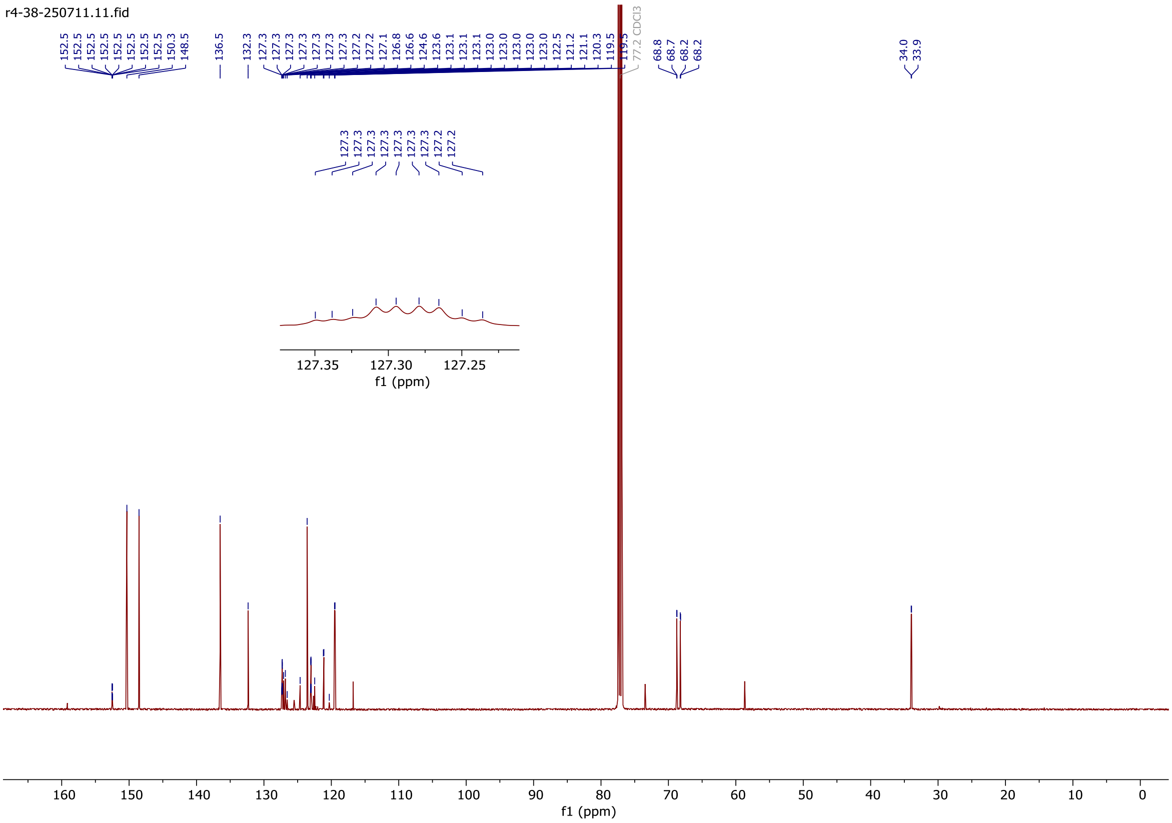

^{31}P NMR spectrum of **6d** (161.9 MHz, chloroform-d)

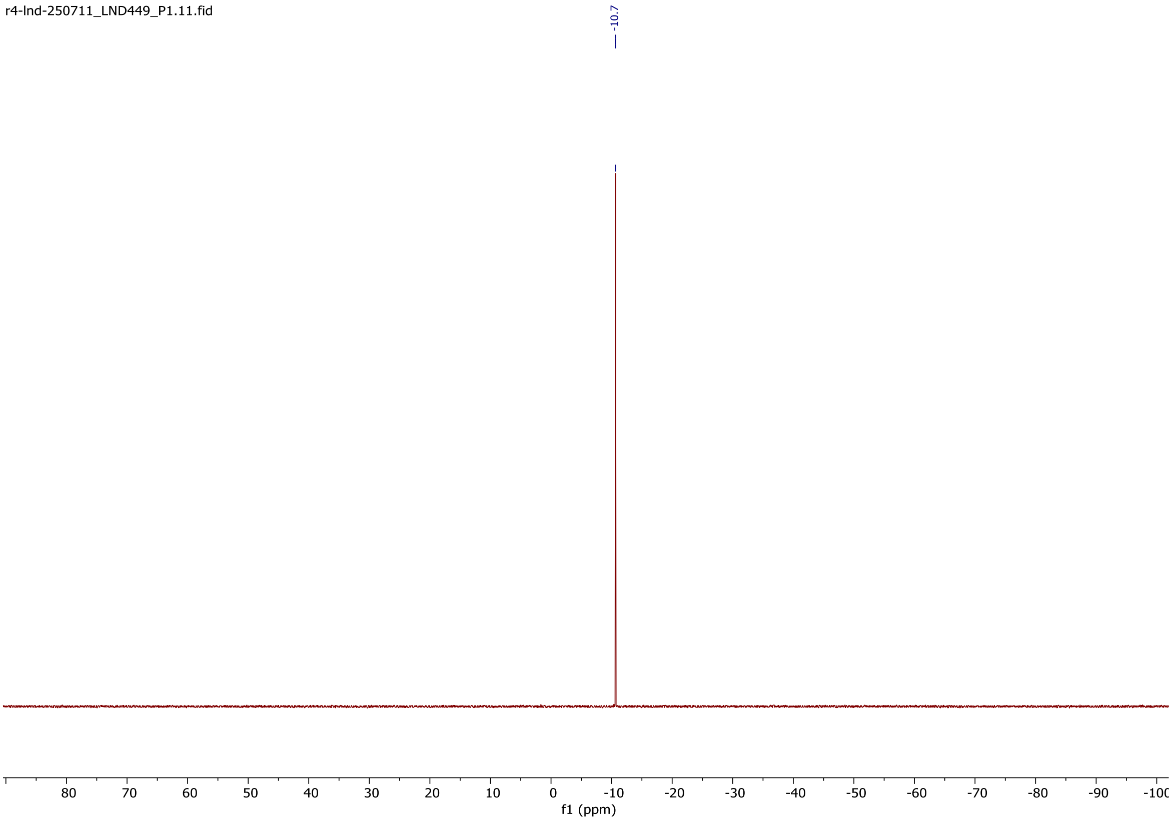

*^{19}F NMR spectrum of **6d** (376.5 MHz, chloroform-d)*

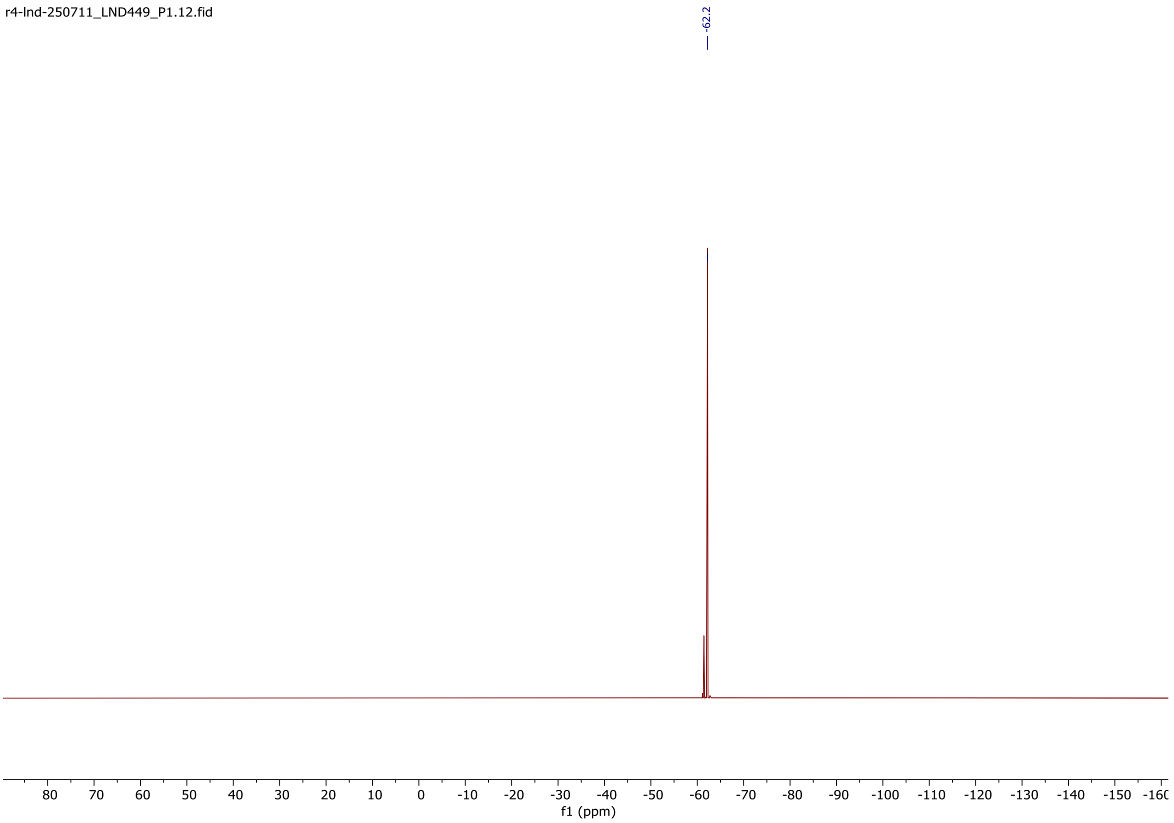

Compound 6g

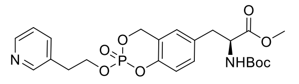

*^{1}H NMR spectrum of **6g** (500 MHz, chloroform-d)*

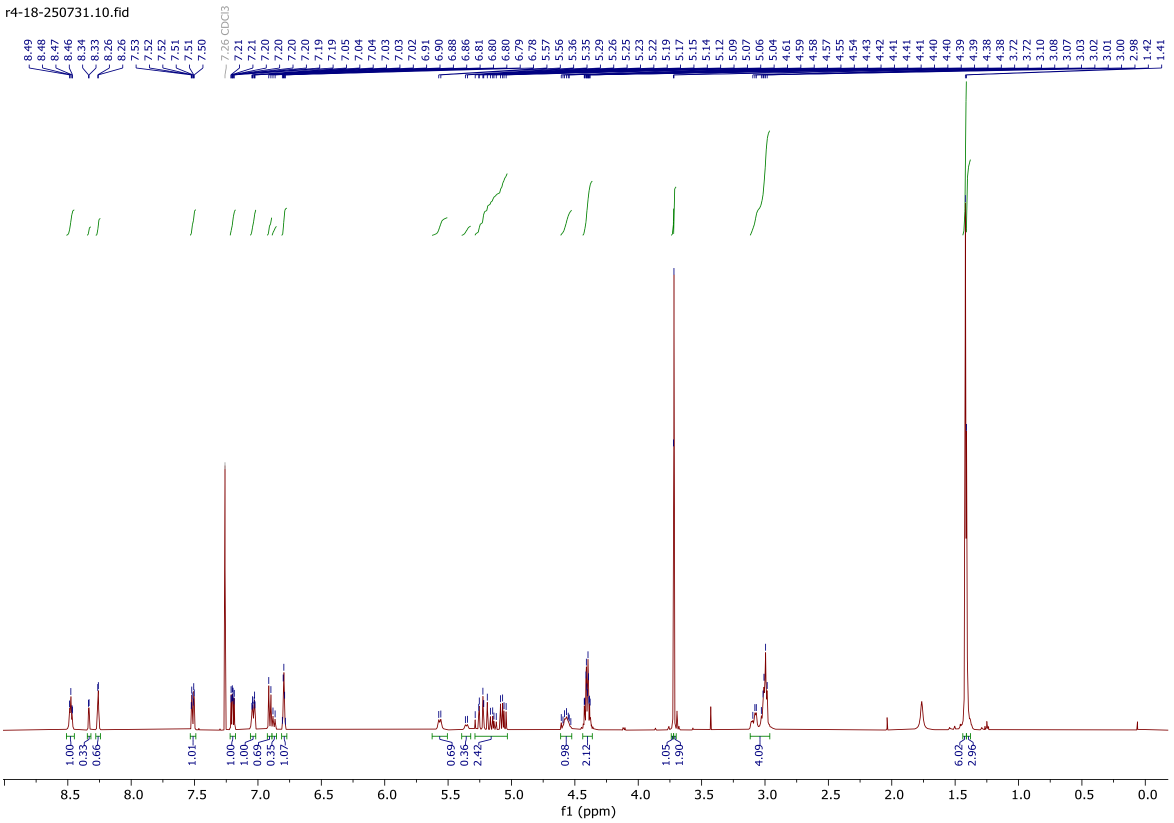

^{13}C NMR spectra of **6g** (125.8 MHz, chloroform-d)

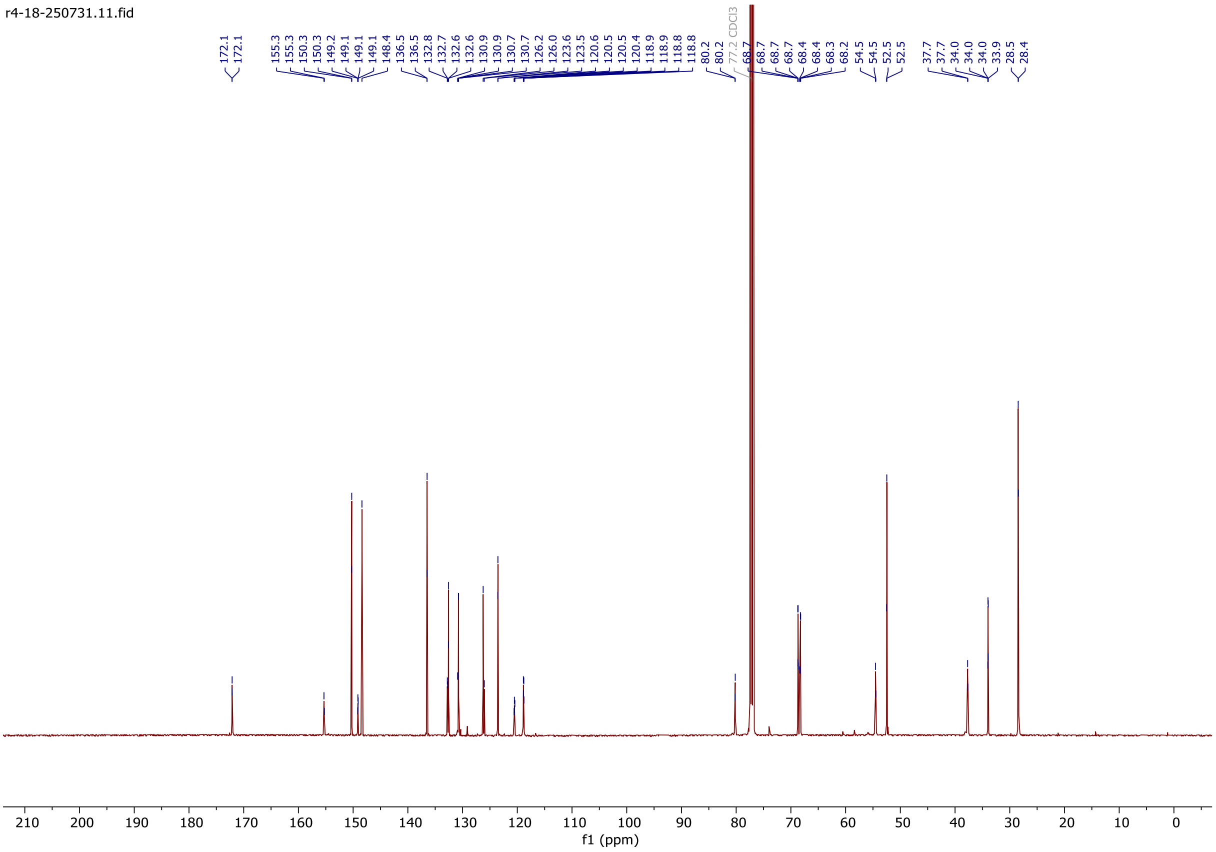

^{31}P NMR spectrum of **6g** (161.9 MHz, chloroform-d)

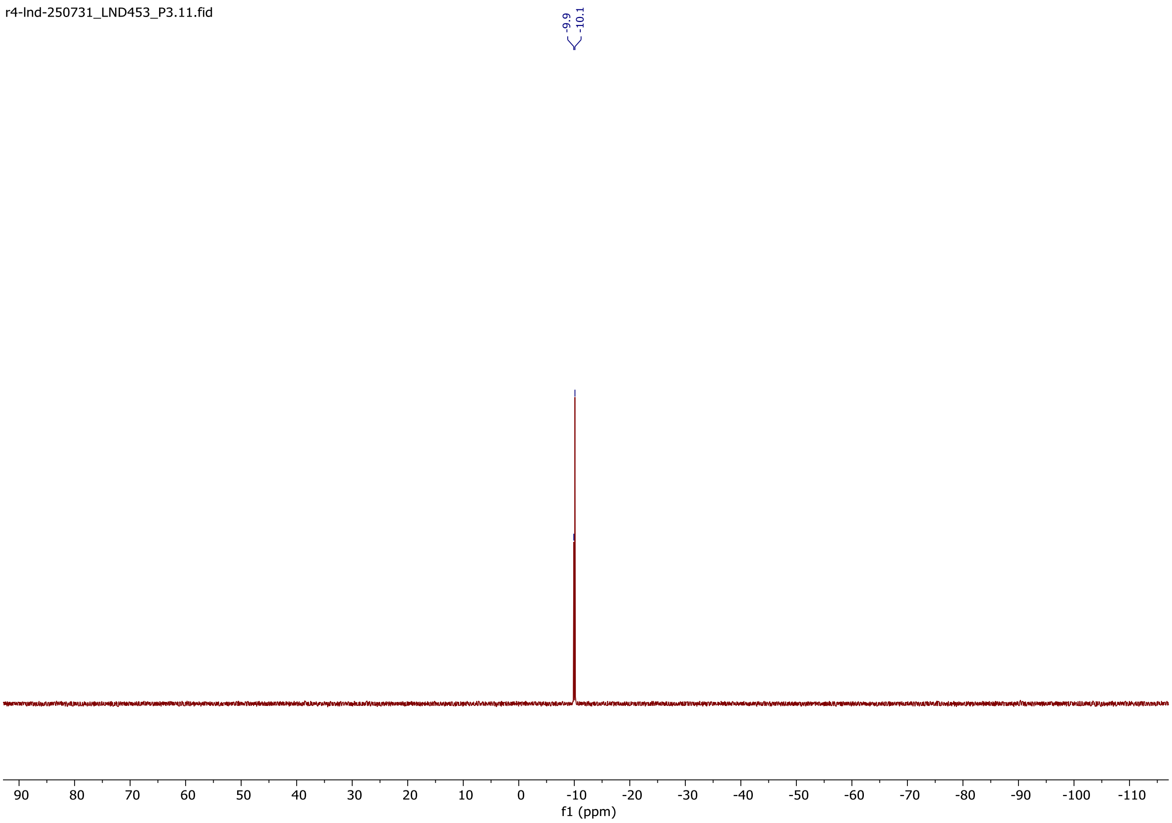

^{13}C NMR spectra of **6g** (125.8 MHz, chloroform-d)

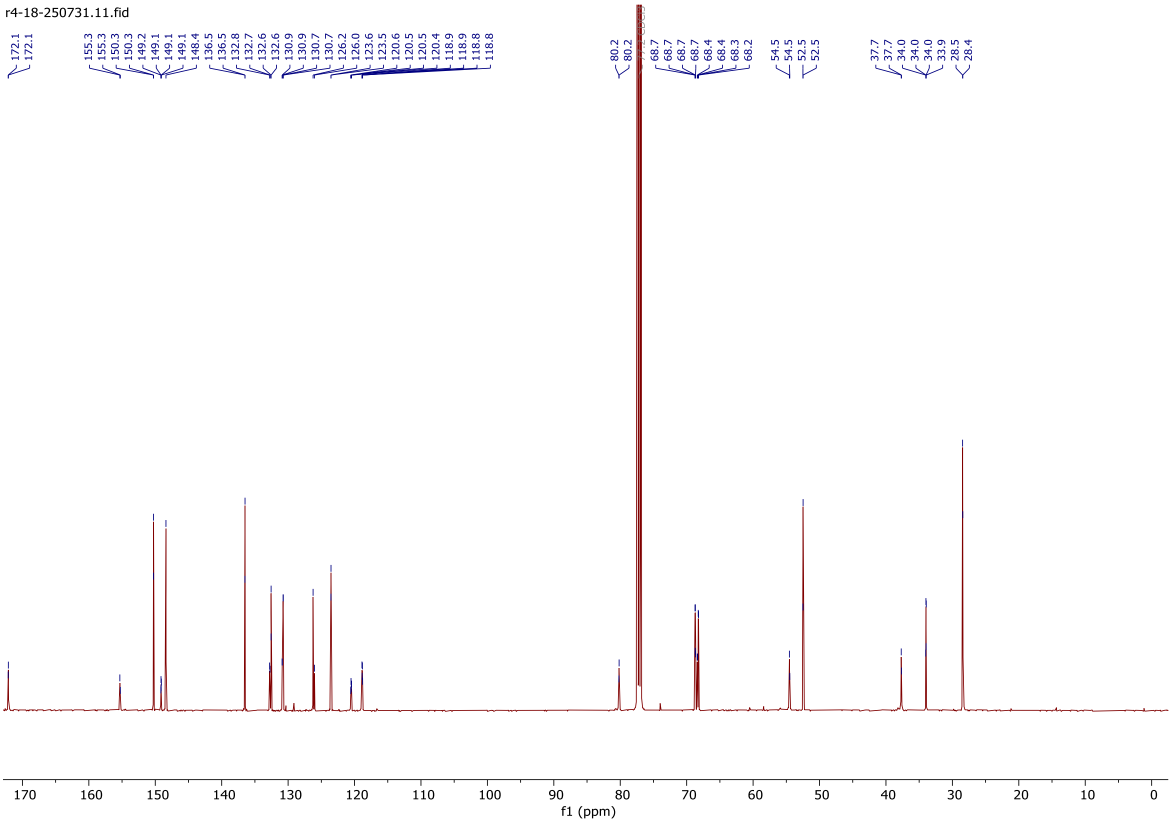

Compound 6h

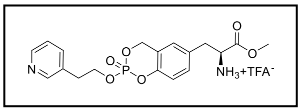

^{1}H NMR spectrum of **6h** (400 MHz, methanol-d4)

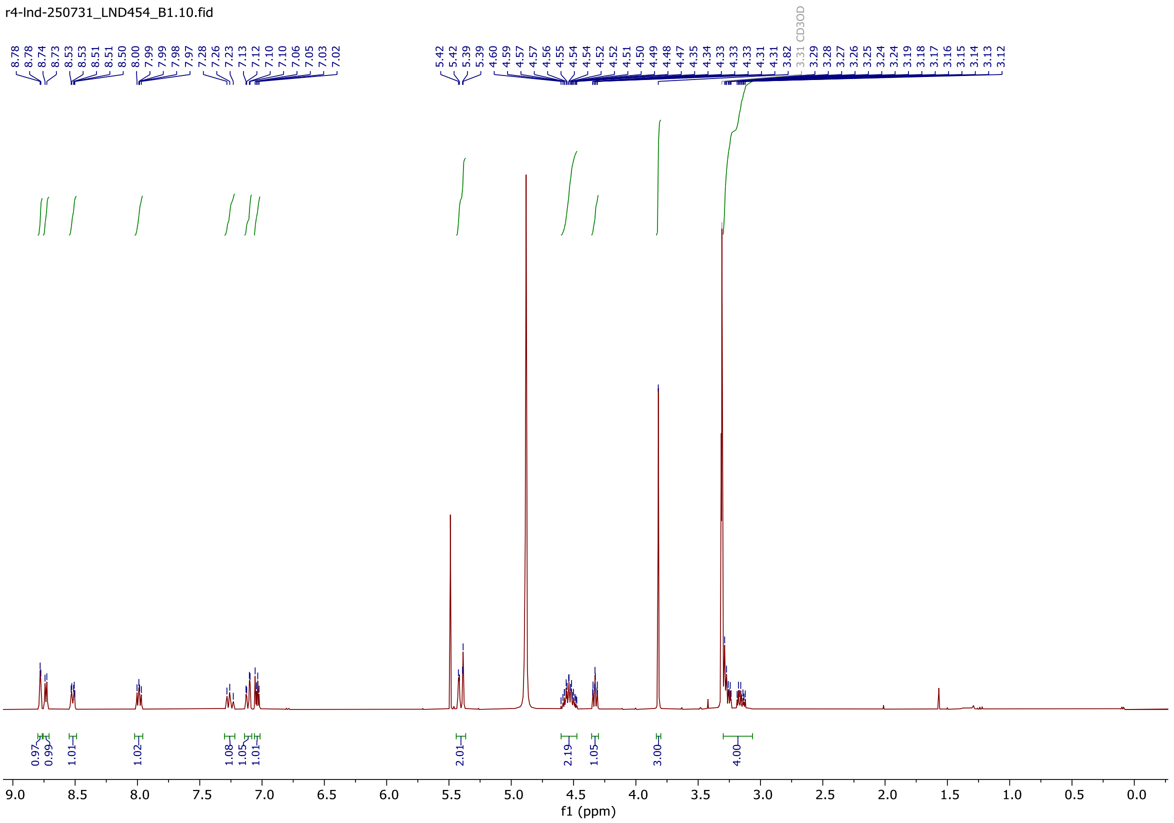

^{31}P NMR spectrum of **6h** (125.8 MHz, methanol-d4)

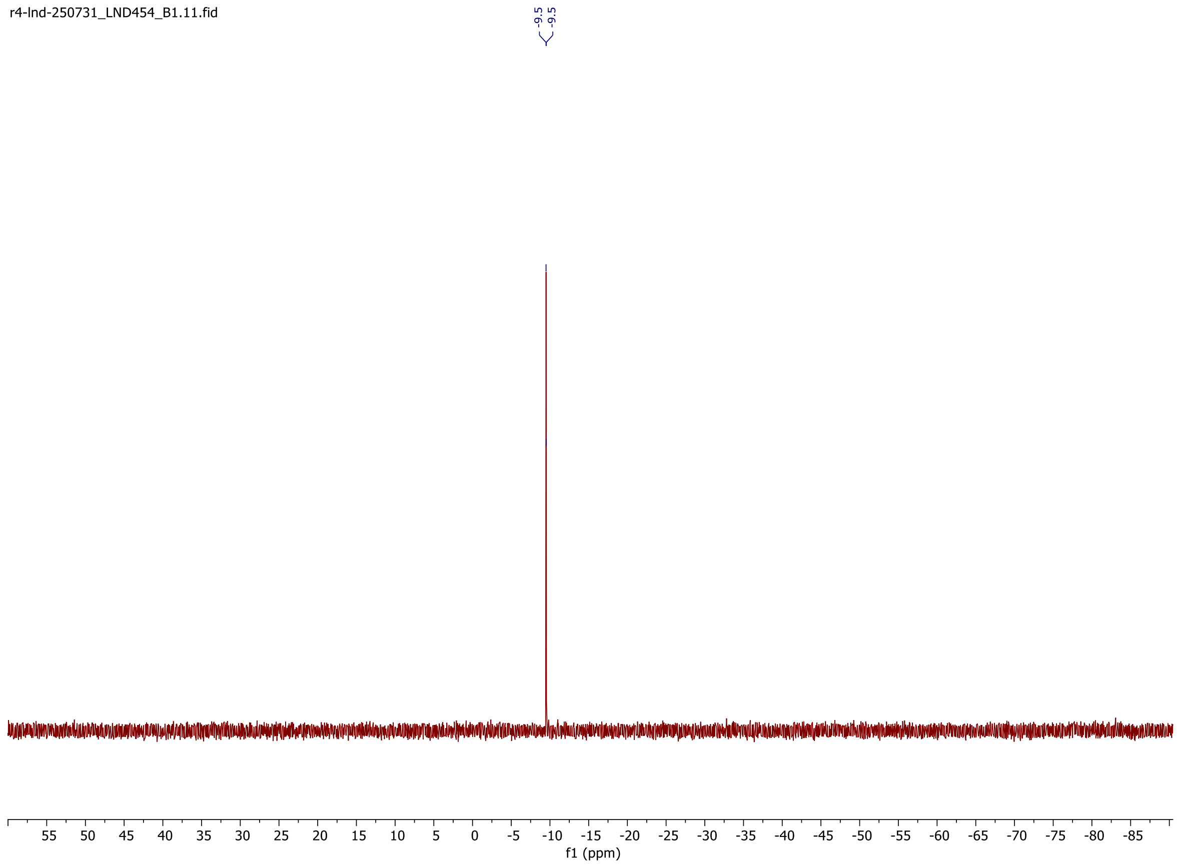

^{13}C NMR spectra of **6h** (161.9 MHz, methanol-d4)

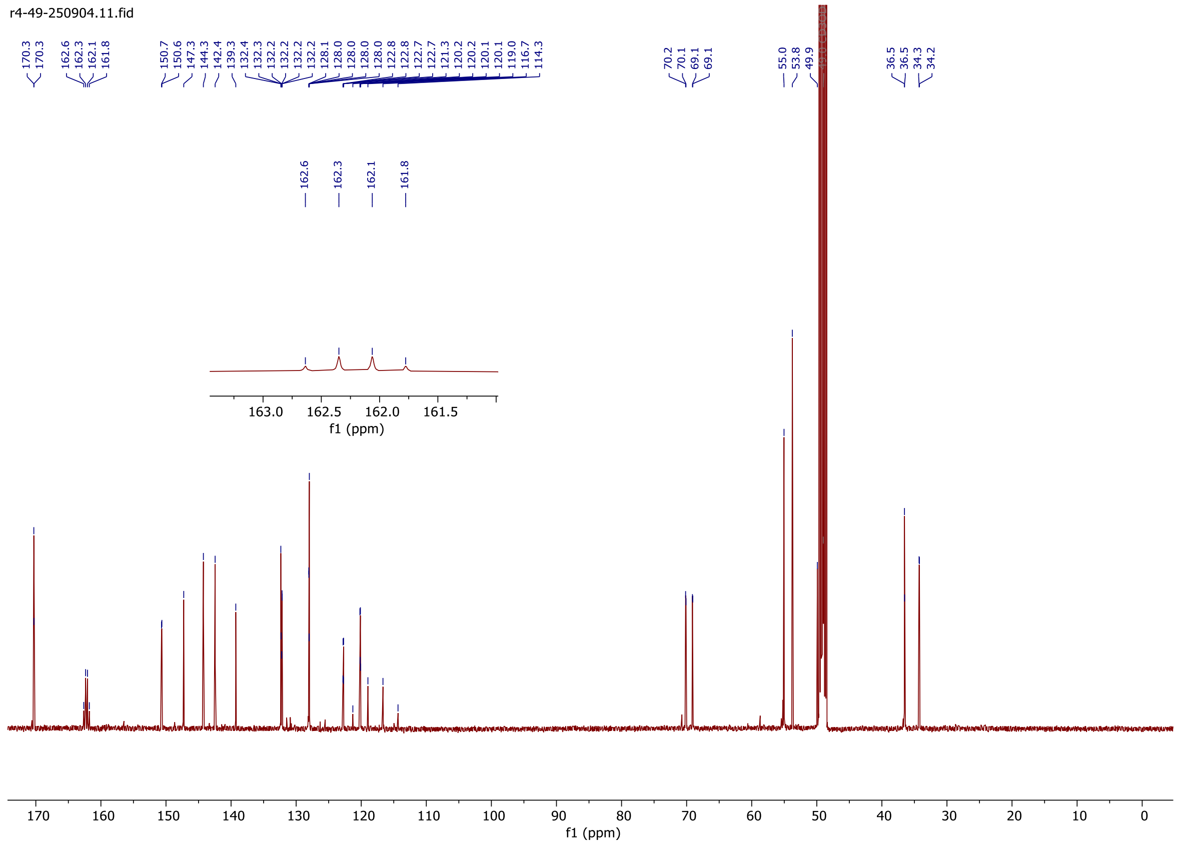

Compound 18

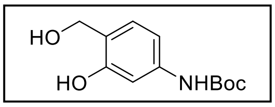

*^{1}H NMR spectra of **18** (500 MHz, chloroform-d)*

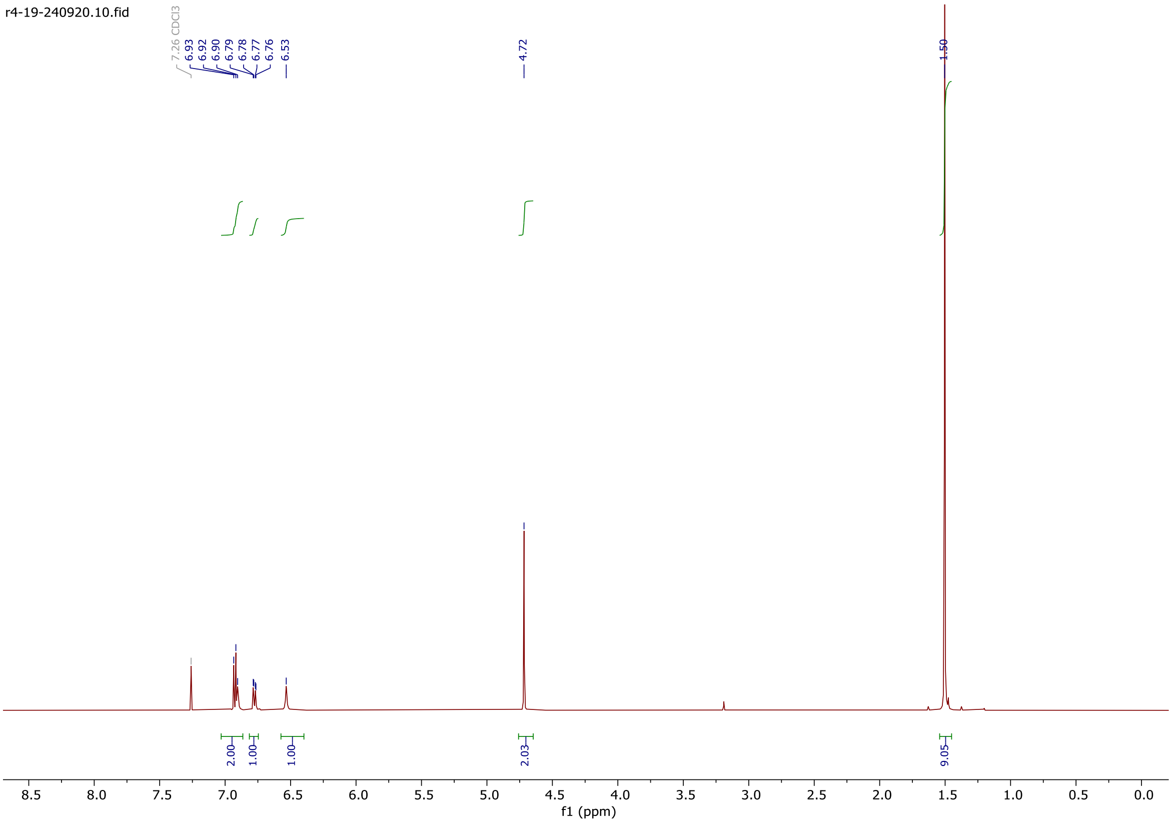

*^{13}C NMR spectra of **18** (125.8 MHz, chloroform-d)*

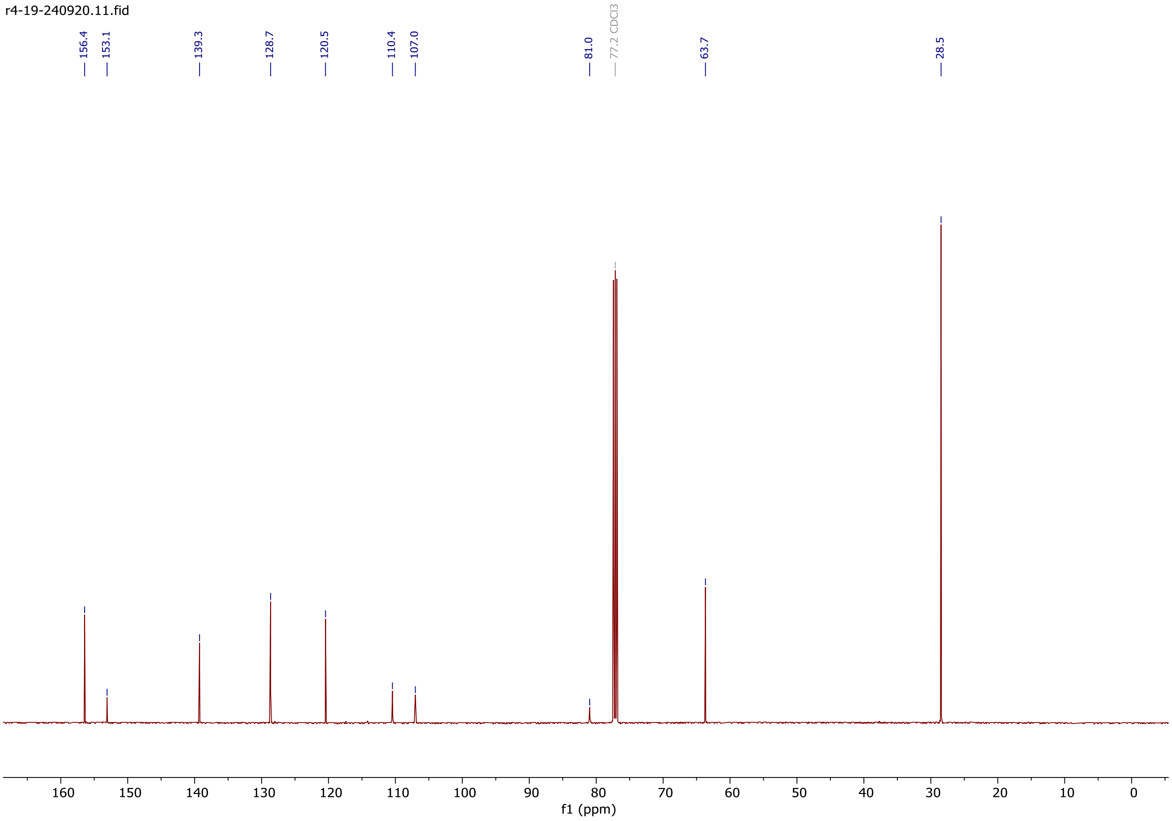

Compound 20

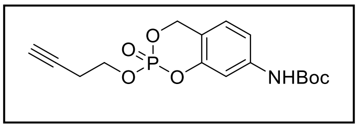

*^{1}H NMR spectrum of **20** (500 MHz, chloroform-d)*

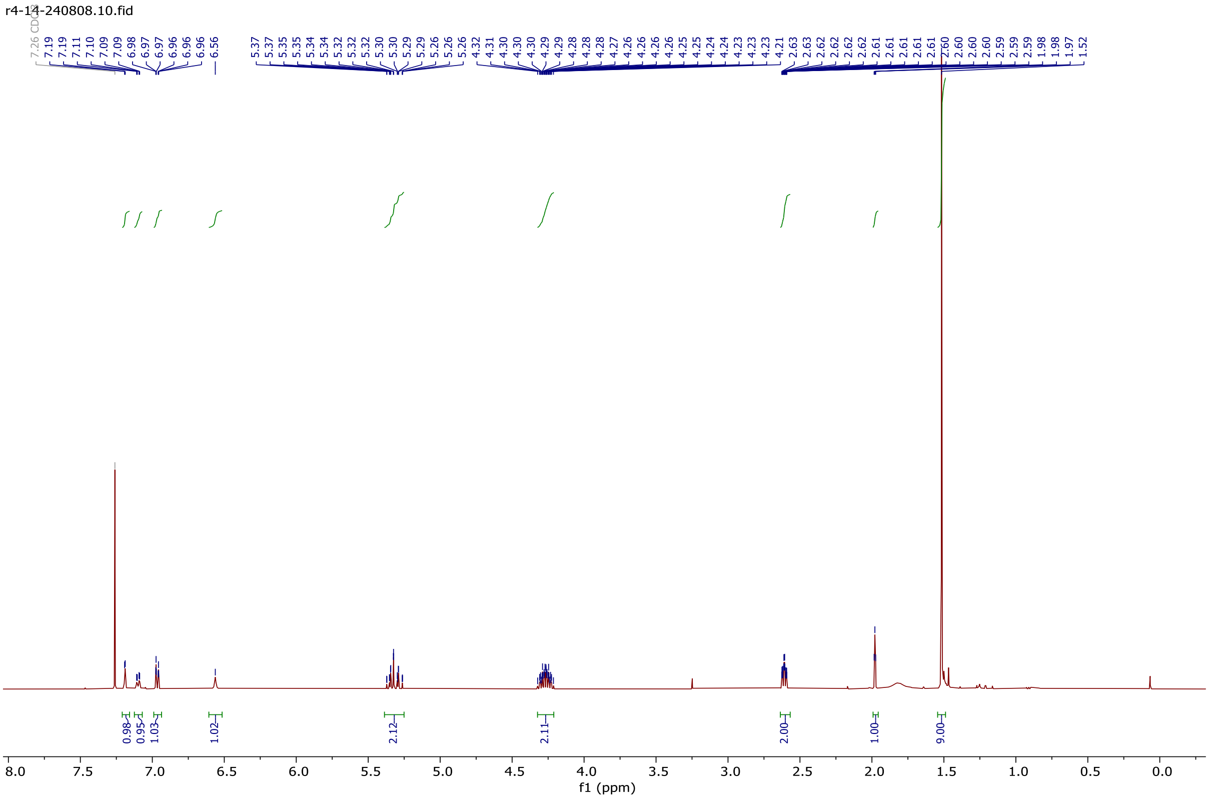

*^{13}C NMR spectra of **20** (125.8 MHz, chloroform-d)*

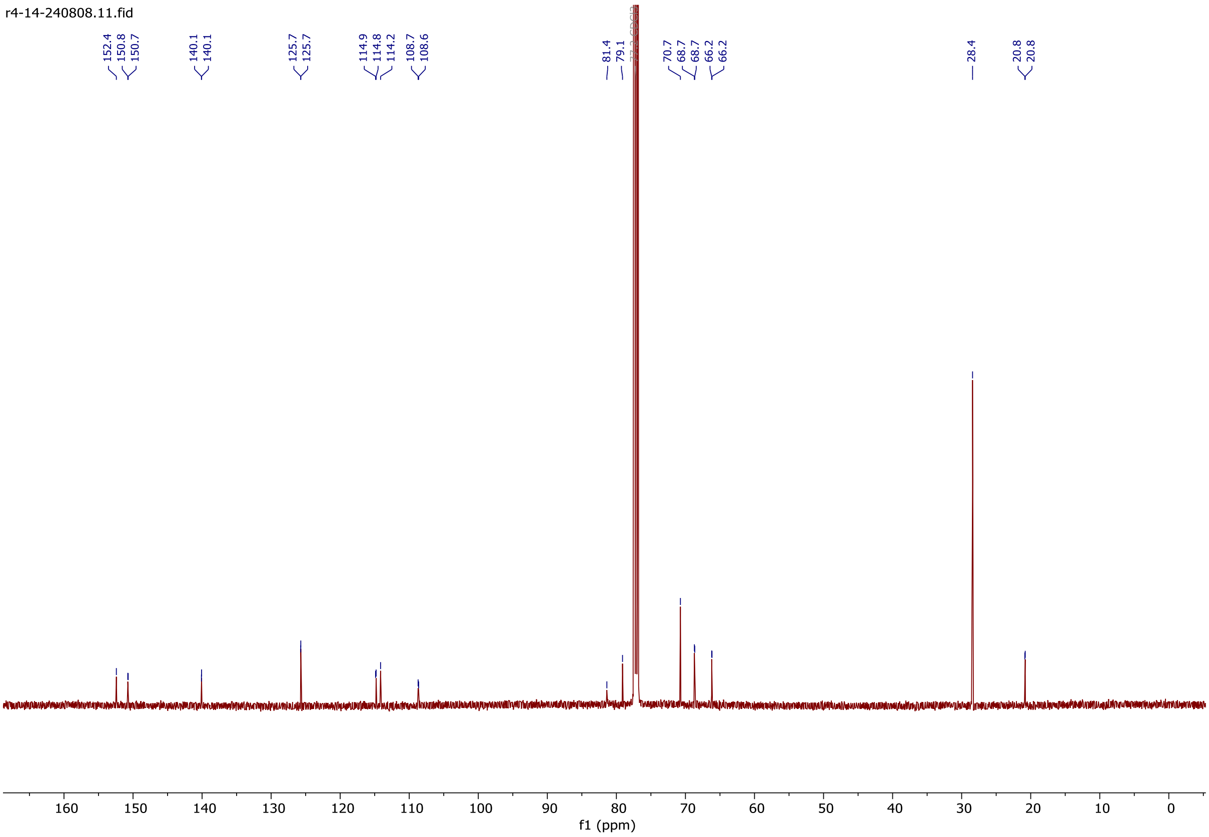

*^{31}P NMR spectrum of **20** (162 MHz, chloroform-d)*

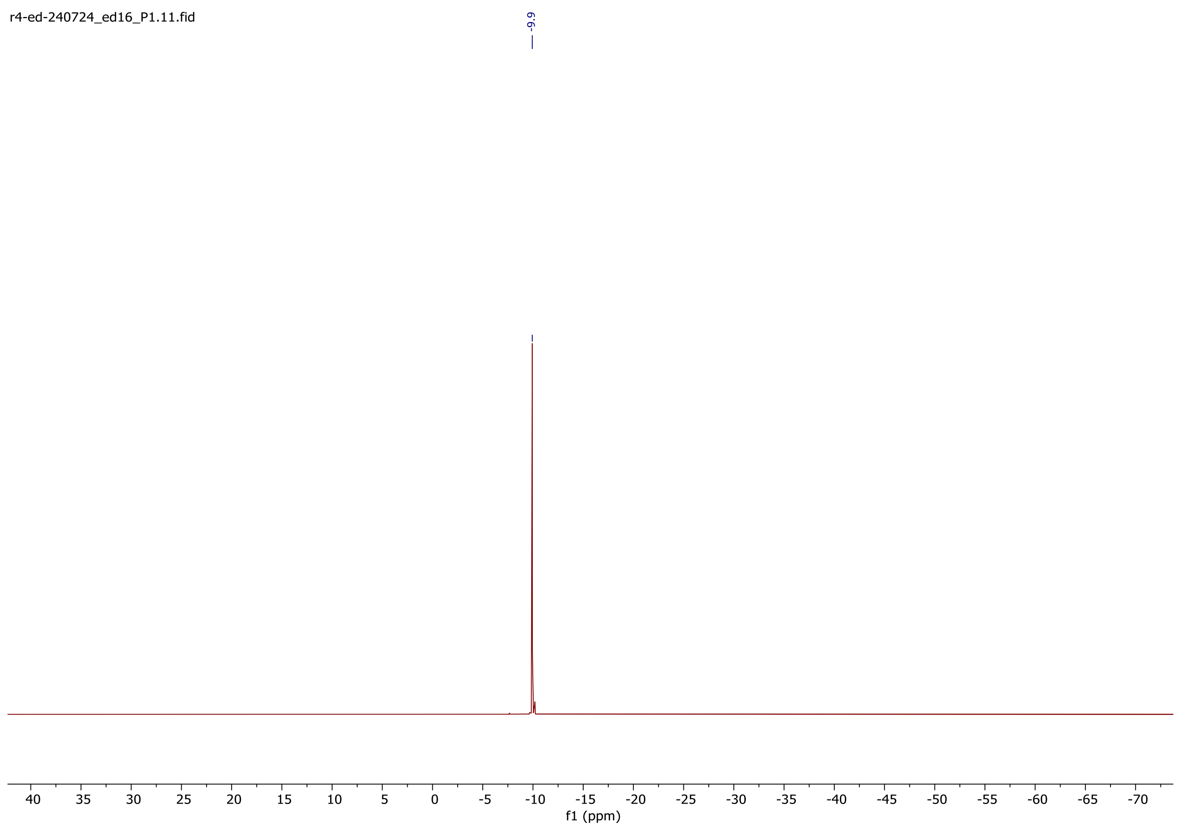

Supplement: Supplementary file 1 [file microorganisms-14-00215-s001.zip › microorganisms-4039316-supplementary.pdf]
